# Molecular Hybrids of Pyazolo[3,4-*b*]pyridine and Triazole: Design, Synthesis and In Vitro Antibacterial Studies

**DOI:** 10.3390/molecules27217647

**Published:** 2022-11-07

**Authors:** Narasimha Rao Bandaru, Parameshwar Makam, Parameswari Akshinthala, Naresh Kumar Katari, Venkanna Banoth, Balakrishna Kolli, Rambabu Gundla

**Affiliations:** 1 Department of Chemistry, GITAM School of Science, GITAM Deemed to be University Hyderabad, Rudraram 502329, India; 2 Department of Chemistry, School of Applied and Life Sciences, Uttaranchal University, Arcadia Grant, Chandanwari, Premnagar, Dehradun 248007, India; 3 Department of Science and Humanities, MLR Institute of Technology, Dundigal, Medchal, Hyderabad, Rudraram 500043, India; 4 Department of Biotechnology, University Post Graduate College of Science and Technology, Jawaharlal Nehru Technological University Hyderabad, Rudraram 500085, India; 5 Department of Chemistry, GITAM School of Science, GITAM Deemed to Be University Visakhapatnam, Visakhapatnam 530045, India

**Keywords:** Pyrazolo[3,4-*b*]pyridine, triazole, in vitro, anti-bacterial, *S. aureus*, *K. pneumoniae*

## Abstract

Antimicrobial resistance is on the rise, and there aren’t enough new treatments to combat it. This might send the modern world back to the pre-antibiotic age. The molecular hybrids of pyrazolo[3,4-*b*]pyridine and triazole have been designed, synthesized, and analyzed for their drug-like molecule nature and in vitro analyses for their inhibition potentials against *S. aureus* and *K. pneumoniae*. The compounds **24** and **27** have been identified as the high potential molecules in this series based on in vitro experiments. Compound **24** has zone of inhibition values of 15 ± 0.82 mm and 14 ± 0.7 mm, whilst compound **27** has zone of inhibition values of 18 ± 0.95 mm and 16 ± 0.82 mm against *S. aureus* and *K. pneumoniae*, respectively. MIC and MIB values for compounds **24** and **27** against *S. aureus* and *K. pneumoniae* are 0.25 and 0.5, respectively.

## 1. Introduction

The emergence of antimicrobial resistance (AMR) bacterial strains, combined with a limited arsenal of viable treatments, threatens to return the modern world to a pre-antibiotic era when simple infections were fatal [1]. A lack of clean water, sanitation, and hygiene (WASH) for both humans and animals, as well as inadequate health-care facilities, a lack of awareness and knowledge, and a lack of legislative enforcement have all contributed to AMR becoming one of the top ten global health threats [2,3]. *Klebsiella pneumonia (K. pneumoniae)* is a bacterium that can cause potentially fatal infections in newborns and patients in intensive care units, as well as pneumonia and bloodstream and gastrointestinal infections, which are frequently brought on by *K. pneumoniae* [4,5]. More than half of the patients treated for *K. pneumoniae* infections in various countries are unable to benefit from carbapenem medications due to the spread of carbapenem antibiotic-resistant strains of *K. pneumoniae* [6]. Another most prevalent organism in hospitals and the general public, *Staphylococcus aureus* (*S. aureus*), has been linked to a variety of clinically significant illnesses, from superficial skin infections to deeply seated invasive infections. *S. aureus* may avoid the effects of antibacterial medications using several methods *Viz.* are alteration of the target protein, improved efflux, lower antibiotic penetration, enzymatic drug degradation, and plasmids. Because bacteria with resistance genes, such as methicillin-resistant *S. aureus* (MRSA), can spread between humans, animals, and the environment, drug-resistant illnesses pose a serious threat to public health yet have few effective therapies [7,8]. Vancomycin is still one of the first-line therapies for MRSA infections, but newly discovered vancomycin-completely resistant MRSA isolates have made the urgent need for the identification of new antibacterial drug classes apparent [9]. Finding medicines with high therapeutic action against both *K. pneumoniae* and *S. aureus* germs is therefore urgently needed [10].

**Figure 1:**
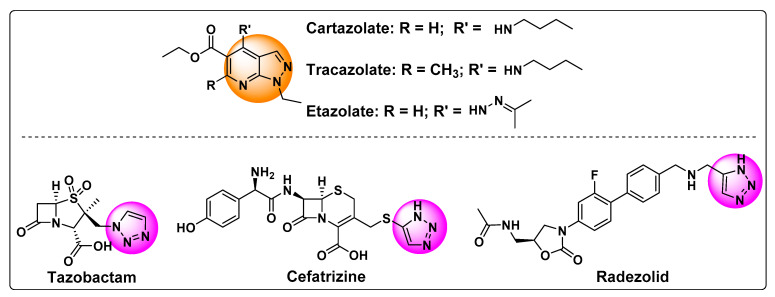
Structures of important pyrazolo[3,4-*b*]pyridine and triazole antibacterial agents.

The synthesis of small compounds with a variety of therapeutic potential has been widely researched using 1*H*-pyrazolo[3,4-*b*]pyridine, one of the most fascinating medicinal chemical scaffolds. Examples include, among others, anti-cancer drugs, anti-diabetic drugs, cardiovascular drugs, enzyme inhibitors, anti-inflammatory drugs, and drugs for the neurological system [11,12]. Similar to this, the 1,2,3-triazole ring is a five-membered heterocyclic motif that can be a bioisostere of amide, ester, carboxylic acid, and other heterocycles. This moiety is easily able to interact with a variety of proteins, enzymes, and receptors in organisms via non-covalent interactions, and numerous structural modifications have resulted in a broad spectrum of activity. With shown anticancer, antibacterial, anti-tubercular, antiviral, antidiabetic, antimalarial, anti-leishmanial, and neuroprotective activities, it is one of the most prevalent frameworks found in bioactive compounds. Some of the pyrazolo[3,4-*b*]pyridine and triazole scaffold containing antibacterial agents have been shown in Figure 1.

## 2. Results and Discussion

### 2.1. Design and Evaluation of Physico-Chemical Properties

**Figure 2:**
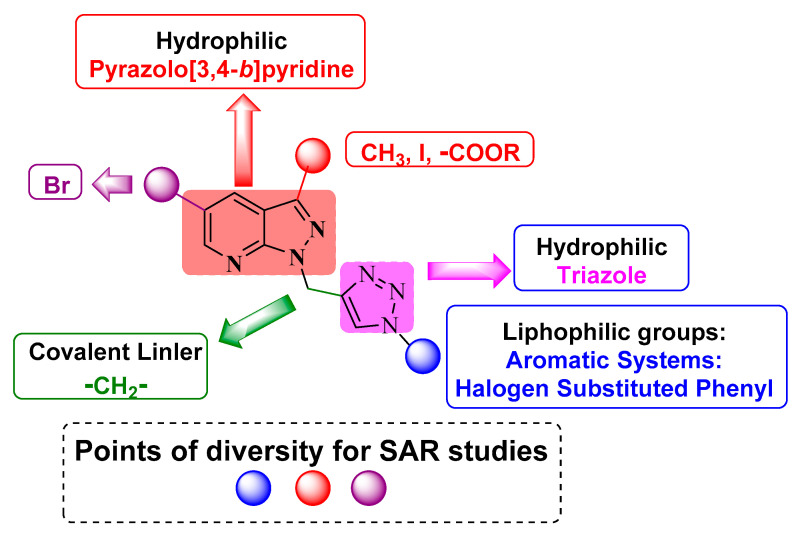
Design of the molecular hybrids of pyazolo[3,4-*b*]pyridine and triazole.

According to the literature, triazole and pyrazolo[3,4-*b*]pyridine scaffolds have been the most extensively studied for their medicinal characteristics among nitrogen-containing heterocycles. To combat drug resistance, there has recently been a surge in interest in the meticulous use of 1,2,3-triazole in the creation of new antibacterial agents [13,14,15,16,17]. Some 1,2,3-triazole-containing hybrids, including cefatrizine, tazobactam, and radezolid, have already been used in clinics or are currently undergoing clinical trials for the treatment of infections brought on by a variety of bacteria, including MRSA [9,13,14,15,16,17]. To create new anti-MRSA candidates, the rational creation of hybrids incorporating 1,2,3-triazoles is an appealing method [18,19]. Therefore, a thoughtful design of molecular hybrids of 1,2,3-triazole derivatives [19] and pyrazolo[3,4-*b*]pyridine [11] may create prospects for the creation of new anti-MRSA drugs. In this study, we continued our attempts to develop anti-infective molecules [20,21,22,23,24] by designing molecular hybrids of pyrazolo[3,4-*b*]pyridine and triazole with structural and functional alterations as shown in Figure 2, synthesizing them, and evaluating how effective they were against the bacteria *K. pneumoniae* and *S. aureus*.

**Figure 3:**
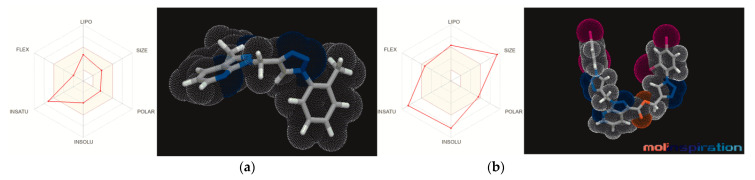
Web and MLP representation of physico-chemical properties of (**a**) Compound **14** and (**b**) Compound **30**.

Enhanced paracellular and transcellular absorption characteristics of compounds with lower molecular weight and lipophilicity are anticipated [16,17,18,19,20,21,22,23,24,25,26,27], as well as a transporter influence on clearance, leading to improved renal excretion [28,29] and mild toxicity [30]. A molecule becomes a drug-like molecule (DLM) when it meets the requirements outlined by the “rule of 5” (Ro5). When a molecule meets the Ro5 requirements, it has a molecular mass of less than 500, a log*P* of less than 5, and less than 5 and 10 donors and acceptors of hydrogen bonds, respectively [31,32,33]. According to the Veber rule, oral bioavailability is greater for molecules with rotatable bonds, polar surface area, and H-bond donors and acceptors that are, respectively, equal to or less than 10, 140, and 12 [34]. In addition, Leeson and colleagues found that approved molecules between 1983 and 2002 contain 16% to 23% more molecular mass, rings, rotatable bonds, and hydrogen bonding groups [35]. Additionally, these traits aid in the creation of molecules with enhanced absorption, distribution, metabolism, excretion, and toxicity (ADMET) [36,37]. Due to this, physicochemical characteristics and how precisely they are regulated now play a crucial role in deciding whether a molecule may be used as a therapeutic candidate and how well it performs throughout key stages of drug development. In light of the aforementioned elements, pyazolo[3,4-*b*]pyridine and triazole molecular hybrids with various structural and functional alterations have been created with and without obeying. The Web and Molecular Lipophilicity Potential (MLP) representations compound **14** and **30** are shown in Figure 3.

**Figure 4:**
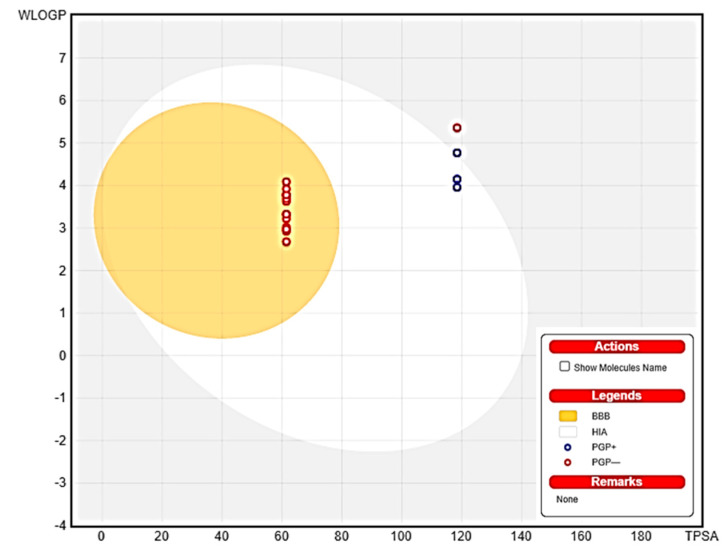
Boiled egg model for molecular hybrids of pyazolo[3,4-*b*]pyridine and triazole.

With compound **14** and compound **30**, respectively, the suggested compounds have molecular weights that range from 304.35 to 798.16. Additionally, it is discovered that the molecular weights of compounds **28**, **26**, **31**, and **29** are, respectively, 574.42, 562.53, 546.37, and 513.46, which do not obey the Ro5. With compound **17** being the lowest and compound **30** being the highest, the iLog*P* values varied from 2.7 to 4.71. There are no hydrogen bond donors and a range of 4 to 10 hydrogen bond acceptors (HA) (HD). Except for molecules **28** to **31**, all compounds have three rotatable bonds. Like this, all the compounds have PTSA values of 61.42, whereas molecules **28** to **31** have values of 118.43. A variety of molecules with a variety of physicochemical characteristics have been produced consequently, and they have been evaluated to see whether they agree with the theories put forward by Lipinski, Veber, and Leeson and the results have been summarized in Table 1 and Table 2. Additionally, it has been expected that certain pharmacokinetic behaviours will occur, including GIA and BBB (brain-blood barrier). By calculating the lipophilicity (WLOG*P* versus TPSA), the Brain Or IntestinaL EstimateD Permeation method (BOILEDEgg) has been visually shown [38]. It is projected that all molecules that fall inside the white ellipse and none that fall inside the yellow ellipse would have superior GIA but poorer BBB properties. Since none of the molecules is in the grey region, they cannot pass through the BBB or the GIA. All substances whose efflux activity in the Central Nervous System (CNS) was predicted by the P-glycoprotein (PGP). The Figure 4 represents the BOILEDEgg model of the designed and synthesized molecules.

### 2.2. Chemistry

**Scheme 1:**
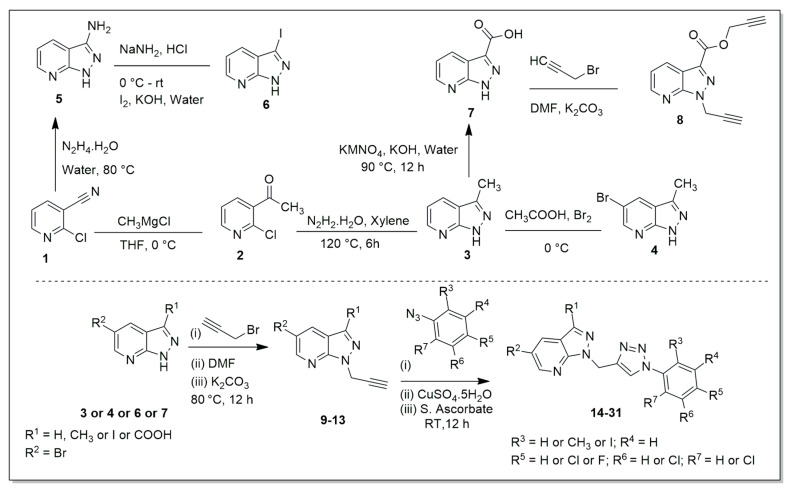
Synthesis of molecular hybrids of pyazolo[3,4-*b*]pyridine and triazole.

The synthesis was initiated by reacting the 2-chloronicotinonitrile, 1 with methyl magnesium chloride at 0 °C for about 3 h, followed by the acidic workup yielding compound **2** with 60% yield [39]. It was further treated with hydrazine hydrate at 120 °C in the presence of xylene for 6 h followed by cooling the reaction mass to 0 °C obtained compound **3** in 95% yield [40]. Compound **3** was subjected to bromination in the presence of a mixture of acetic acid and bromine at 0–5 °C. Upon completion of the reaction, the solid obtained is filtered to get compound **4** in a 73% yield [41]. Following the method described in the literature with minor modifications, the amine substituted pyazolo[3,4-*b*]pyridine, **5** has been achieved by treating compound **4** with hydrazine hydrate with 90% yield and which has subsequently undergone nucleophilic substitution with the Iodine to generate compound **6** with 93% yield [42,43]. The compound **3** oxidised to generate the corresponding acid **7** with 80% yield by following the method described in the literature with minor modification as shown in Scheme 1.

As shown in sheme 1, the next step involves the alkylation of compounds **3**, **4**, **6** and **7** with 3-bromoprop-1-yne using potassium carbonate as base and DMF as a solvent and stirring the reaction at 80 °C for 12 h to get compounds, 9–13 in 75–95% yield by using the method described in the literature with minor modification [44,45]. The final step of the synthesis involves the application of click chemistry on compounds 9–13, which has been carried out by heating with the corresponding azide for one hour. After the reaction is complete, water is added [44,45]. The precipitated solid is filtered to get crude compounds, **14** to **31**, which were further purified by column chromatography to obtain the spectrally pure desired compounds of this study. All the synthetic pathways are outlined ins 1 and the structures of all the synthesised molecular hybrids of pyazolo[3,4-*b*]pyridine and triazole have been represented in Table 1. Please see experimental and supporting information for structural and spectral characterization.

### 2.3. In Vitro Anti-Bacterial Studies

**Figure 5:**
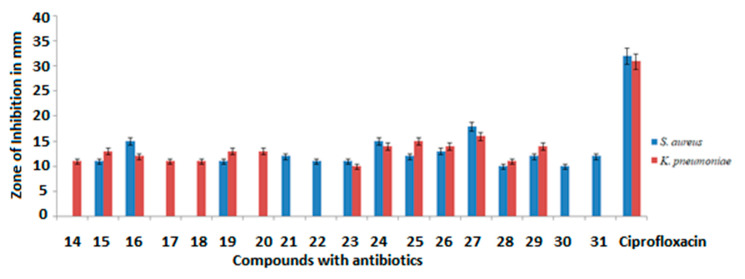
Zone of inhibition of pyazolo[3,4-*b*]pyridine and triazole molecular hybrids.

All the synthesized compounds, **14** to **31** were classified into four categories and subjected to their in vitro antibacterial activity against activity against Gram-positive *S. aureus* and Gram-negative *K, pneumoniae* bacterial strains by using standard in vitro methods, i.e., zone of inhibition, MIC, and MBC. The class I molecules (**14** to **18**) contains a methyl group substitution at the 3rd position of pyazolo[3,4-*b*]pyridine moiety and with variations of functional group substitutions on the phenyl system of phenyl-1*H*-1,2,3-triazole. All the molecules were found to be in an almost equipotent zone of inhibition, MIC and MBC with 11, 0.25, and 0.5 respectively against both *K. pneumonia* and *S. aureus*. The class II molecules (18 to 22) contain a bromo and methyl substitutions at the 5th and 3rd positions of pyazolo[3,4-*b*]pyridine moiety and with a similar variation of functional group substitutions on the phenyl system of phenyl-1*H*-1,2,3-triazole as that of group I molecules. Surprisingly like group I molecules, all the molecules were found to be almost equipotent with a zone of inhibition, MIC and MBC values of 11, 0.25, and 0.5 respectively against both *K. pneumonia* and *S. aureus*. The zone of inhibition of pyazolo[3,4-*b*]pyridine and triazole molecular hybrids are summarized and graphically represented in Figure 5.

**Figure 6:**
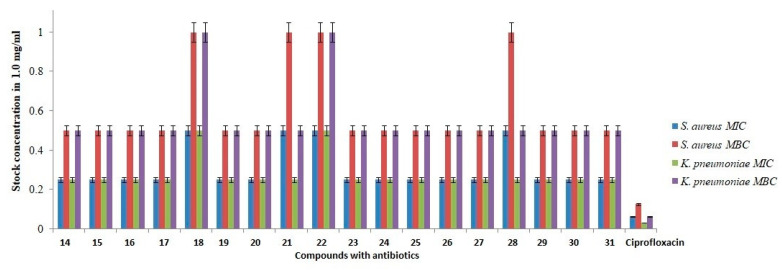
MIC and MBC of pyazolo[3,4-*b*]pyridine and triazole molecular hybrids.

The interesting results are found in the group III molecules, where iodo substitutions at 3rd positions of pyazolo[3,4-*b*]pyridine moiety have been introduced and with similar variations of functional group substitutions on phenyl system of phenyl-1*H*-1,2,3-triazole. The **24**, which has the methyl group substitution at the 2nd position of the phenyl ring has exhibited the zone of inhibition values of 15 ± 0.82 and 14 ± 0.75 against *S. aureus* and *K. pneumonia* respectively. The MIC and MIB values of the molecule, **24** has found to be 0.25 and 0.5 against *S. aureus* and *K. pneumonia* respectively. With these results, molecule **24** was found to be the second-best potent molecule after molecule **27**. The best potent molecule of this study is **27**, which has the structural modification of chloro substitution at the 3rd position of the phenyl ring. With the zone of inhibition values of 18 ± 0.95 and 16 ± 0.82 against *S. aureus* and *K. pneumonia* respectively the molecule **27** stand out to be the best potent molecule in this group. Similarly, The MIC and MIB values of the molecule, **27** has found to be 0.25 and 0.5 against *S. aureus* and *K. pneumonia* respectively. The group-IV molecules contain two substituted phenyl-1*H*-1,2,3-triazole attachments at 1st and 3rd position with alkyl and ester functional group linkers. For the group IV molecules, all the molecules are found to be almost equipotent with zone of inhibition, MIC and MBC values of 11, 0.25, and 0.5, respectively, against both *K. pneumonia* and *S. aureus*. The summary of MIC and MBC all the of pyazolo[3,4-*b*]pyridine and triazole molecular hybrids have been graphically represented in Figure 6.

### 2.4. Materials and Methods

#### 2.4.1. Chemistry

Oven-dried glassware was used to carry out all the reactions and the progression of reactions was monitored by thin-layer chromatography (TLC). VEEGO VMP-DS melting point apparatus was used to determine melting points. Melting points were uncorrected and determined in open-end capillaries. Nicolet-6700 spectrometer was used to record IR spectra using KBr. ^1^H, and ^13^C NMR spectra were recorded on Bruker Advance 400 spectrometer using DMSO-*d*_6_ and CCl_4_ in a 1:1 ratio as solvent (See Supplementary Materials). The chemical shift value (*δ*) is expressed in parts per million units and is measured relative to SiMe_4_ (*δ* = 0.00) as the internal standard. Coupling constants (*J*) are measured in Hz. Multiplicities are expressed as s (singlet), d (doublet), t (triplet), q (quartet), m (multiplet) or broad (br). HPLC analyses were carried out by using the SCL-10ATVP SHIMADZU instrument.

**General procedure for the synthesis of compounds 14 to 31:** To a solution of alkyne derivatives (0.14 mmol) dissolved in THF, CuSO_4_.5H_2_O (0.014 mmol) added in portion wise followed by a catalytic amount of sodium ascorbate. After stirring for 12 h at room temperature, the reaction mixture was diluted with H_2_O (10 mL) and extracted with EtOAc. The combined organic phases afforded, after usual workup, and the crude was purified using column chromatography.

*3-Methyl-1-((1-(o-tolyl)-1H-1,2,3-triazol-4-yl)methyl)-1H-pyazolo[3,4-b]pyridine***(14)**: Cream colour solid; Yield 82%; IR (KBr) cm^−1^: 3138, 3105, 2921, 2904, 2877, 2842, 1591, 1562, 1502, 1383, 1359, 1329, 1255, 1176, 1102,1040, 778, 768, 685; ^1^H NMR (400 MHz, CDCl_3_ + SiMe_4_) *δ*_H_ 8.57 (d, *J* = 2.1 Hz, 1H), 8.13 (d, *J* = 2.1 Hz, 1H), 7.91 (s, 1H), 7.71 (t, *J* = 2.0 Hz, 1H), 7.59 (dt, *J* = 7.7, 1.8 Hz, 1H), 7.47–7.35 (m, 2H), 5.83 (s, 2H), 2.55 (s, 3H); ^13^C NMR (101 MHz, CDCl_3_ + SiMe_4_) *δ*_C_ 149.6, 149.2, 143.4, 136.3, 133.6, 131.4, 131.3, 129.8, 126.7, 125.9, 123.9, 116.7, 111.8, 77.2, 42.3, 17.8, 12.4; HPLC = 98.36%, Rf: 0.6 (50% EtOAc:Hexane).

*1-((1-(2-Chloro-6-methylphenyl)-1H-1,2,3-triazol-4-yl)methyl)-3-methyl-1H-pyazolo[3,4-b]pyridine***(15)**: Brown colour solid; Yield 80%; m.p. 65–68 °C; IR (KBr) cm^−1^: 3138, 3105, 2921, 2904, 2877, 2842, 1591, 1562, 1502, 1383, 1359, 1329, 1255, 1176, 1102, 1040, 778, 768, 685; ^1^H NMR (400 MHz, CDCl_3_ + SiMe_4_) *δ*_H_ 8.55 (d, *J* = 2.1 Hz, 1H), 8.12 (d, *J* = 2.2 Hz, 1H), 7.67 (s, 1H), 7.39–7.30 (m, 2H), 7.28–7.21 (m, 2H), 5.87 (s, 2H), 2.54 (s, 3H), 2.02 (s, 3H); 13C NMR (101 MHz, CDCl_3_ + SiMe_4_) *δ*_C_ 149.6, 149.2, 143.4, 140.8, 138.1, 131.7, 131.3, 130.9, 129.3, 127.7, 124.7, 116.7, 111.8, 42.4, 17.8, 12.4; MS (ESI) 339.2 (M + H^+^), HPLC = 96.63%, Rf: 0.4 (50% EtOAc:Hexane).

*1-((1-(4-Chloro-2-iodophenyl)-1H-1,2,3-triazol-4-yl)methyl)-3-methyl-1H-pyazolo[3,4-b]pyridine***(16)**: White colour solid; Yield 82%; m.p. 140–142 °C; IR (KBr) cm^−1^: 3142, 3086, 2987, 2950, 1598, 1577, 1488, 1379, 1358, 1343, 1266, 1242, 1142, 1040, 870-850, 772, 696, 600, 500; ^1^H NMR (400 MHz, CDCl_3_ + SiMe_4_) *δ*_H_ 8.56 (dd, *J* = 4.6, 1.5 Hz, 1H), 8.04–7.93 (m, 2H), 7.79 (s, 1H), 7.44 (dd, *J* = 8.5, 2.3 Hz, 1H), 7.26 (s, 3H), 7.11 (dd, *J* = 8.0, 4.6 Hz, 1H), 5.90 (s, 2H), 2.58 (s, 3H); ^13^C NMR (101 MHz, CDCl_3_ + SiMe_4_) *δ*_C_ 150.8, 148.8, 144.0, 141.5, 139.4, 138.6, 136.5, 129.4, 129.2, 128.2, 124.4, 116.1, 115.3, 94.0, 42.0, 12.4; MS (ESI) 451.0 (M + H^+^), HPLC = 99.35%, Rf: 0.4 (50% EtOAc:Hexane).

*1-((1-(4-Fluorophenyl)-1H-1,2,3-triazol-4-yl)methyl)-3-methyl-1H-pyazolo[3,4-b]pyridine***(17)**: Off-white colour solid; Yield 81%; m.p. 134–135 °C; IR (KBr) cm^−1^: 3132, 3005, 2921, 2894, 2877, 2852, 1737, 1600, 1575, 1503, 1385, 1362, 1340, 1270, 1175, 1140, 1044, 770, 701; ^1^H NMR (400 MHz, CDCl_3_ + SiMe_4_) *δ*_H_ 8.56 (dd, *J* = 4.6, 1.5 Hz, 1H), 8.01 (dd, *J* = 8.0, 1.5 Hz, 1H), 7.85 (s, 1H), 7.64 (dd, *J* = 9.0, 4.6 Hz, 2H), 7.20–7.10 (m, 3H), 5.88 (s, 2H), 2.58 (s, 3H); ^13^C NMR (101 MHz, CDCl_3_ + SiMe_4_) *δ*_C_ 148.9, 144.9, 141.5, 129.4, 122.5, 122.4, 120.7, 116.6, 116.4, 116.2, 115.2, 42.0, 12.4; MS (ESI) 309.1 (M + H^+^), HPLC = 98.80%, Rf: 0.5 (50% EtOAc:Hexane).

*1-((1-(3-Chlorophenyl)-1H-1,2,3-triazol-4-yl)methyl)-3-methyl-1H-pyazolo[3,4-b]pyridine***(18)**: White colour solid; Yield 83%; m.p. 91–92 °C; IR (KBr) cm^−1^; ^1^H NMR (400 MHz, CDCl_3_ + SiMe_4_) *δ*_H_ 8.56 (d, *J* = 4.6 Hz, 1H), 8.01 (d, *J* = 8.0 Hz, 1H), 7.89 (s, 1H), 7.71 (s, 1H), 7.58 (d, *J* = 7.7 Hz, 1H), 7.41 (dd, *J* = 13.9, 6.1 Hz, 2H), 7.12 (dd, *J* = 8.0, 4.6 Hz, 1H), 5.88 (s, 2H), 2.59 (s, 3H); ^13^C NMR (101 MHz, CDCl_3_ + SiMe_4_) *δ*_C_ 148.9, 145.0, 141.6, 137.7, 135.3, 130.6, 129.4, 128.6, 120.6, 120.5, 118.4, 116.2, 15.2, 41.9, 12.4; MS (ESI) 325.1 (M + H^+^), HPLC = 99.92%, Rf: 0.4 (50% EtOAc:Hexane).

*5-Bromo-1-((1-(2-chloro-6-methylphenyl)-1H-1,2,3-triazol-4-yl)methyl)-3-methyl-1H-pyazolo[3,4-b]pyridine***(19):** White colour solid; Yield 85%; m.p. 140–142 °C; IR (KBr) cm^−1^: 3138, 3105, 2921, 2904, 2877, 2842, 1591, 1562, 1502, 1383, 1359, 1329, 1255, 1176, 1102,1040, 778, 768, 685; ^1^H NMR (400 MHz, CDCl_3_ + SiMe_4_) *δ*_H_ 8.55 (d, *J* = 2.1 Hz, 1H), 8.12 (d, *J* = 2.2 Hz, 1H), 7.67 (s, 1H), 7.39–7.30 (m, 2H), 7.28–7.21 (m, 2H), 5.87 (s, 2H), 2.54 (s, 3H), 2.02 (s, 3H); ^13^C NMR (101 MHz, CDCl_3_ + SiMe_4_) *δ*_C_ 149.6, 149.2, 143.4, 140.8, 138.1, 131.7, 131.3, 130.9, 129.3, 127.7, 124.7, 116.7, 111.8, 42.4, 17.8, 12.4; MS (ESI) 419.0 (M + H^+^), HPLC = 97.05%, Rf: 0.4 (50% EtOAc:Hexane).

*5-Bromo-1-((1-(4-fluorophenyl)-1H-1,2,3-triazol-4-yl)methyl)-3-methyl-1H-pyazolo[3,4-b]pyridine***(20)**: Light Yellow colour solid; Yield 84%; m.p. 140–141 °C; IR (KBr) cm^−1^: 3476, 3430, 3253, 3131, 3082, 2923, 2853, 1667, 1592, 1516, 1455, 1370, 1328, 1255, 1176, 1101, 1060, 775, 681; ^1^H NMR (400 MHz, CDCl_3_ + SiMe_4_) *δ*_H_ 8.57 (d, *J* = 2.1 Hz, 1H), 8.12 (d, *J* = 2.1 Hz, 1H), 7.87 (s, 1H), 7.67–7.63 (m, 2H), 7.21–7.15 (m, 2H), 5.83 (s, 2H), 2.54 (s, 3H); ^13^C NMR (101 MHz, CDCl_3_ + SiMe_4_) *δ*_C_ 149.7, 149.2, 144.4, 140.9, 133.1, 131.3, 122.5, 122.4, 120.8, 116.7, 116.4, 111.8, 42.2, 12.4; MS (ESI) 389.0 (M + H^+^), HPLC = 98.72%, Rf: 0.4 (50% EtOAc:Hexane).

*5-Bromo-1-((1-(4-chlorophenyl)-1H-1,2,3-triazol-4-yl)methyl)-3-methyl-1H-pyazolo[3,4-b]pyridine***(21)**: White colour solid; Yield 86%; m.p. 147–148 °C; IR (KBr) cm^−1^: 3150, 3139, 3083, 3066, 3052, 2922, 2873, 1591, 1566, 1487, 1462, 1384, 1328, 1236, 1184, 1096, 1076, 770, 671; ^1^H NMR (400 MHz, CDCl_3_ + SiMe_4_) *δ*_H_ 8.57 (d, *J* = 2.1 Hz, 1H), 8.13 (d, *J* = 2.1 Hz, 1H), 7.89 (s, 1H), 7.65–7.61 (m, 2H), 7.48–7.44 (m, 2H), 5.83 (s, 2H), 2.54 (s, 3H); ^13^C NMR (101 MHz, CDCl_3_ + SiMe_4_) *δ*_C_ 149.7, 144.5, 141.0, 135.3, 134.5, 131.3, 129.8, 121.6, 120.5, 116.7, 111.8, 42.1, 12.4; MS (ESI) 405.0 (M + H^+^), HPLC = 98.03%, Rf: 0.4 (50% EtOAc:Hexane).

*5-Bromo-1-((1-(3-chlorophenyl)-1H-1,2,3-triazol-4-yl)methyl)-3-methyl-1H-pyazolo[3,4-b]pyridine***(22)**: White colour solid; Yield 82%; m.p. 130–131 °C; IR (KBr) cm^−1^: 3146, 3139, 3083, 3066, 3052, 2922, 2873, 1590, 1560, 1499, 1460, 1377, 1332, 1253, 1227, 1114, 1076, 767, 715; ^1^H NMR (400 MHz, CDCl_3_ + SiMe_4_) *δ*_H_ 8.56 (d, *J* = 4.6 Hz, 1H), 8.01 (d, *J* = 8.0 Hz, 1H), 7.89 (s, 1H), 7.71 (s, 1H), 7.58 (d, *J* = 7.7 Hz, 1H), 7.41 (dd, *J* = 13.9, 6.1 Hz, 2H), 7.12 (dd, *J* = 8.0, 4.6 Hz, 1H), 5.88 (s, 2H), 2.59 (s, 3H); ^13^C NMR (101 MHz, CDCl_3_ + SiMe_4_) *δ*_C_ 148.9, 145.0, 141.6, 137.7, 135.3, 130.6, 129.4, 128.6, 120.6, 120.5, 118.4, 116.2, 115.7, 41.6, 12.3; MS (ESI) 403.67 (M + H^+^), HPLC = 98.19%, Rf: 0.4 (50% EtOAc:Hexane).

*1-((1-(2-Chloro-6-methylphenyl)-1H-1,2,3-triazol-4-yl)methyl)-3-iodo-1H-pyazolo[3,4-b]pyridine***(23)**: White colour solid; Yield 85%; m.p. 159–160 °C; IR (KBr) cm^−1^: 3146, 3139, 3083, 3066, 3052, 2922, 2873, 1595, 1571, 1487, 1454, 1389, 1317, 1271, 1235, 1159, 1081, 1037, 767, 715; ^1^H NMR (400 MHz, CDCl_3_ + SiMe_4_) *δ*_H_ 8.61 (dd, *J* = 4.5, 1.5 Hz, 1H), 7.82 (dd, *J* = 8.1, 1.5 Hz, 1H), 7.72 (s, 1H), 7.39–7.31 (m, 2H), 7.26–7.19 (m, 2H), 5.99 (d, *J* = 0.7 Hz, 2H), 2.03 (s, 3H); ^13^C NMR (101 MHz, CDCl_3_ + SiMe_4_) *δ*_C_ 150.4, 150.2, 143.1, 138.1, 134, 131.7, 130.9, 130.5, 129.3, 127.7, 124.9, 117.8, 90.5, 42.9, 17.8, MS (ESI) 450.9 (M + H^+^); HPLC = 97.33%, Rf: 0.3 (50% EtOAc:Hexane).

*3-Iodo-1-((1-(o-tolyl)-1H-1,2,3-triazol-4-yl)methyl)-1H-pyazolo[3,4-b]pyridine***(24):** White colour solid; Yield 82%; m.p. 132–133 °C; IR (KBr) cm^−1^: 3150, 3101, 3045, 2965, 2921, 2822, 2349, 1596, 1568, 1498, 1457, 1391, 1315, 1285, 1227, 1167, 1127, 792, 712; ^1^H NMR (400 MHz, CDCl_3_ + SiMe_4_) *δ*_H_ 8.78–8.49 (m, 1H), 8.10–7.65 (m, 2H), 7.56–7.08 (m, 5H), 5.97 (s, 2H), 2.18 (s, 3H); ^13^C NMR (101 MHz, CDCl_3_ + SiMe_4_) *δ*_C_ 150.2, 143.1, 136.3, 133.6, 131.4, 130.5, 129.8, 126.7, 125.9, 124.2, 120.5, 117.8, 42.8, 17.8; MS (ESI) 417.1 (M + H^+^), HPLC = 98.67%, *R*f: 0.5 (50% EtOAc:Hexane).

*1-((1-(4-Fluorophenyl)-1H-1,2,3-triazol-4-yl)methyl)-3-iodo-1H-pyazolo[3,4-b]pyridine***(25)**: Off-white colour solid; Yield 85%; m.p. 145–146 °C; IR (KBr) cm^−1^: 3732, 3147, 3088, 2922, 2851, 1885, 1738, 1597, 1566, 1514, 1446, 1412, 1316, 1295, 1233, 1152, 1126, 829, 762, 712; ^1^H NMR (400 MHz, CDCl_3_ + SiMe_4_) *δ*_H_ 8.62 (dd, *J* = 4.6, 1.6 Hz, 1H), 7.93 (s, 1H), 7.82 (dd, *J* = 8.1, 1.5 Hz, 1H), 7.69–7.63 (m, 2H), 7.24–7.16 (m, 3H), 5.96–5.93 (m, 2H); ^13^C NMR (101 MHz, CDCl_3_ + SiMe_4_) *δ*_C_ 150.3, 144.1, 130.6, 122.6, 122.5, 121.1, 120.5, 117.9, 116.7, 116.5, 90.7, 42.7; MS (ESI) 421.1 (M + H^+^), HPLC = 98.30%, *R*f: 0.5 (50% EtOAc:Hexane).

*1-((1-(4-Chloro-2-iodophenyl)-1H-1,2,3-triazol-4-yl)methyl)-3-iodo-1H-pyazolo[3,4-b]pyridine***(26)**: White colour solid; Yield 81%; m.p. 163–164 °C; IR (KBr) cm^−1^: 3142, 3085, 3058, 2920, 2851, 1882, 1732, 1595, 1563, 1484, 1443, 1384, 1321, 1302, 1244, 1172,1121, 814, 795, 737; ^1^H NMR (400 MHz, CDCl_3_ + SiMe_4_) *δ*_H_ 8.62 (dd, *J* = 4.5, 1.6 Hz, 1H), 7.97 (d, *J* = 2.2 Hz, 1H), 7.88 (s, 1H), 7.82 (dd, *J* = 8.0, 1.6 Hz, 1H), 7.46 (dd, *J* = 8.5, 2.3 Hz, 1H), 7.33 (d, *J* = 8.4 Hz, 1H), 7.21 (dd, *J* = 8.1, 4.5 Hz, 1H), 5.97 (s, 2H); ^13^C NMR (101 MHz, CDCl_3_ + SiMe_4_) *δ*_C_ 150.3, 150.2, 143.3, 139.5, 138.5, 136.6, 130.6, 129.3, 128.3, 124.7, 120.5, 117.8, 94.0, 42.7; MS (ESI) 562.9 (M + H^+^), HPLC = 98.59%, *R*f: 0.4 (50% EtOAc:Hexane).

*1-((1-(3-Chlorophenyl)-1H-1,2,3-triazol-4-yl)methyl)-3-iodo-1H-pyazolo[3,4-b]pyridine***(27)**: White colour solid; Yield 86%; m.p. 140–142 °C; ^1^H NMR (400 MHz, CDCl_3_ + SiMe_4_) *δ*_H_ 8.63 (d, *J* = 5.4 Hz, 1H), 7.96 (s, 1H), 7.86–7.79 (m, 1H), 7.73 (s, 1H), 7.59 (d, *J* = 7.3 Hz, 1H), 7.42 (dd, *J* = 13.6, 5.9 Hz, 2H), 7.22 (dd, *J* = 8.1, 4.6 Hz, 1H), 5.95 (s, 2H); ^13^C NMR (101 MHz, CDCl_3_ + SiMe_4_) *δ*_C_ 150.3, 144.3, 137.7, 135.4, 130.7, 130.6, 128.8, 120.8, 120.7, 120.6, 118.5, 117.9, 90.7, 42.7, HPLC = 97.00%, *R*f: 0.5 (50% EtOAc:Hexane).

*(1-(2-Chloro-5-methylphenyl)-1H-1,2,3-triazol-4-yl)methyl1-((1-(2-chloro-6-methylphenyl) -1H-1,2,3-triazol-4-yl)methyl)-1H-pyazolo[3,4-b]pyridine-3-carboxylate***(28)**: White colour solid; Yield 84%; m.p. 112–113 °C; IR (KBr) cm^−1^: 3138, 2955, 2923, 2853, 1736, 1713, 1597, 1573, 1487, 1460, 1371, 1266, 1225, 1150, 1120, 866, 771, 721; ^1^H NMR (400 MHz, CDCl_3_ + SiMe_4_) *δ*_H_ 8.66 (d, *J* = 5.1 Hz, 1H), 8.52 (d, *J* = 8.5 Hz, 1H), 7.92 (s, 1H), 7.77 (s, 1H), 7.45–7.31 (m, 5H), 7.26 (q, *J* = 7.6, 6.8 Hz, 4H), 6.09 (s, 2H), 5.73 (s, 2H), 2.08 (s, 2H), 2.01 (s, 3H); ^13^C NMR (101 MHz, CDCl_3_ + SiMe_4_) *δ*_C_ 161.6, 149.8, 142.4, 142.4, 138.0, 138.0, 133.9, 131.6, 131.4, 131.0, 130.9, 129.4, 129.3, 127.7, 127.6, 126.3, 125.1, 119.5, 115.5, 58.1, 43.4, 29.6, 17.8, 17.7; MS (ESI) 576.1 (M + H^+^), HPLC = 96.14%, *R*f: 0.4 (50% EtOAc:Hexane).

*(1-(4-Fluorophenyl)-1H-1,2,3-triazol-4-yl)methyl 1-((1-(4-fluorophenyl)-1H-1,2,3-triazol-4-yl)methyl)-1H-pyazolo[3,4-b]pyridine-3-carboxylate***(29)**: Off-white colour solid; Yield 83%; m.p. 139–140 °C; IR (KBr) cm^−1^: 3148, 3080, 2922, 2851, 1725, 1710, 1601, 1572, 1515, 1466, 1367, 1269, 1224, 1166, 1119, 831, 769, 695; ^1^H NMR (400 MHz, CDCl_3_ + SiMe_4_) *δ*_H_ 8.66 (dd, *J* = 4.5, 1.6 Hz, 1H), 8.52 (dd, *J* = 8.1, 1.6 Hz, 1H), 8.16 (s, 1H), 7.96 (s, 1H), 7.74–7.69 (m, 2H), 7.66–7.61 (m, 2H), 7.33 (dd, *J* = 8.1, 4.5 Hz, 1H), 7.25–7.13 (m, 5H), 6.04 (s, 2H), 5.68 (s, 2H); ^13^C NMR (101 MHz, CDCl_3_ + SiMe_4_) *δ*_C_ 161.7, 150.6, 149.9, 143.4, 143.2, 133.0, 131.4, 122.8, 122.6, 122.5, 122.5, 122.5, 121.3, 119.6, 116.8, 116.7, 116.6, 116.5, 115.4, 58.1, 43.2; MS (ESI) 514.2 (M + H^+^), HPLC = 95.27%, *R*f: 0.3 (50% EtOAc:Hexane).

*(1-(4-Chloro-2-iodophenyl)-1H-1,2,3-triazol-4-yl)methyl 1-((1-(4-chloro-2-iodophenyl)-1H-1,2,3-triazol-4-yl)methyl)-1H-pyazolo[3,4-b]pyridine-3-carboxylate***(30)**: Off-white colour solid; Yield 80%; m.p. 147–149 °C; IR (KBr) cm^−1^: 1730, 1707, 1573, 1487, 1462, 1371, 1266, 1227, 1153, 1118, 868, 771, 694; ^1^H NMR (400 MHz, CDCl_3_ + SiMe_4_) *δ*_H_ 8.66 (dd, *J* = 4.4, 1.4 Hz, 1H), 8.52 (dd, *J* = 8.1, 1.6 Hz, 1H), 8.07 (s, 1H), 8.00 (d, *J* = 2.2 Hz, 1H), 7.96 (d, *J* = 2.2 Hz, 1H), 7.93 (s, 1H), 7.47 (ddd, *J* = 17.3, 8.5, 2.2 Hz, 2H), 7.40–7.29 (m, 3H), 6.07 (s, 2H), 5.70 (s, 2H); ^13^C NMR (101 MHz, CDCl_3_ + SiMe_4_) *δ*_C_ 161.6, 149.9, 142.6, 142.5, 139.6, 139.5, 131.4, 129.4, 129.4, 128.3, 126.3, 125.0, 119.6, 94.1, 58.0, 43.2; MS (ESI) 799.9 (M + H^+^), HPLC = 96.28%, *R*f: 0.2 (50% EtOAc:Hexane).

*(1-(3-Chlorophenyl)-1H-1,2,3-triazol-4-yl)methyl 1-((1-(3-chlorophenyl)-1H-1,2,3-triazol-4-yl)methyl)-1H-pyazolo[3,4-b]pyridine-3-carboxylate* **(31)**: Off-white colour solid; Yield 84%; m.p. 171–172 °C; IR (KBr) cm^−1^: 3090, 2853, 1720, 1710, 1593, 1573, 1488, 1463, 1371, 1266, 1225, 1166, 1120, 872, 774, 672; ^1^H NMR (400 MHz, CDCl_3_ + SiMe_4_) δ_H_ 8.67 (dd, *J* = 4.5, 1.6 Hz, 1H), 8.52 (dd, *J* = 8.1, 1.6 Hz, 1H), 8.21 (s, 1H), 8.00 (s, 1H), 7.79 (t, *J* = 2.0 Hz, 1H), 7.71 (t, *J* = 1.8 Hz, 1H), 7.64 (ddd, *J* = 7.8, 2.2, 1.5 Hz, 1H), 7.60–7.54 (m, 1H), 7.51–7.37 (m, 4H), 7.34 (dd, *J* = 8.1, 4.5 Hz, 1H), 6.04 (s, 2H), 5.69 (s, 2H); ^13^C NMR (101 MHz, CDCl_3_ + SiMe_4_) δ_C_ 161.7, 150.7, 150.0, 143.6, 143.4, 137.6, 135.6, 135.5, 131.4, 130.8, 130.7, 129.0, 128.9, 122.5, 121.0, 120.8, 120.7, 119.6, 118.4, 115.4, 58.0, 43.2; MS (ESI) 546.1 (M + H^+^), HPLC = 98.87%, Rf: 0.3 (50% EtOAc:Hexane).

#### 2.4.2. In Vitro Assay for Evaluation of Antibacterial Activity

##### Qualitative Test (Agar Well Diffusion Method)

All the Gram-negative and Gram-positive bacterial strains used for the present study were obtained from the Department of Microbiology, Osmania General Hospital, Hyderabad. All strains were tested for purity by standard microbiological methods. The bacterial stock cultures were maintained on Mueller-Hinton agar slants and stored at 4 °C. An agar-well diffusion method was employed to an evaluation of antibacterial activities of test compounds. DMSO was used as a negative control. The bacterial strains were reactivated from stock cultures by transferring them into Mueller-Hinton broth and incubating at 37 °C for 18 h. A final inoculum containing 106 colonies forming units (1 × 106 CFU/mL) was added aseptically to the MHA medium and poured into sterile Petri dishes. Different test compounds at a concentration of 100 µg per well were added to wells (8 mm in diameter) punched on an agar surface. Plates were incubated overnight at 37 °C and the diameter of the inhibition zone (DIZ) around each well was measured in mm. Experiments were performed in triplicates.

##### Quantitative Estimation (Minimum Inhibitory Concentration)

The minimum inhibitory concentration (MIC) and minimum bactericidal concentration (MBC) were determined by the micro-broth dilution method done in 96 well plates according to standard protocol. A 2-fold serial dilution of the compounds, with the appropriate antibiotic, was prepared. Initially, 100 µL of MH broth was added to each well plate. Then 100 µL of compound or antibiotic was taken from the stock solution and dissolved in the first well plate. Serial dilution was done to obtain different concentrations. The stock concentrations were 1.0 mg/mL. 24 h culture turbidity was adjusted to match 0.5 McFarland standards which correspond to 1 × 10^8^ CFU/mL. The standardized suspension (100 µL) of bacteria was added to all the wells except the antibiotic control well and the 96 well plates were incubated at 37 °C for 24 h. After 24 h of incubation 40 µL of MTT (3-(4,5-dimethlthiazol-2-yl)-2,5-diphenyltrazolium bromide) reagent (0.1 mg/mL in 1x PBS) was added to all the wells. MIC was taken as the lowest concentration which did not show any growth which was visually noted from the blue colour developed by MTT. Subcultures were made from clear wells and the lowest concentration that yielded no growth after subculturing was taken as the MBC.

## 3. Conclusions

In summary, diversely functionalized pyazolo[3,4-b]pyridine and triazole molecular hybrids have been designed and synthesised using multistep synthetic strategies. The analyses of the anti-bacterial properties of the synthesised molecules were evaluated against gram-positive and negative pathogens. The compounds **24** and **27** showed potential antibacterial activity with the zone of inhibition values of 15 ± 0.82 and 18 ± 0.95 respectively against *S. aureus.* Similarly, **24** and **27** showed 14 ± 0.75 and 16 ± 0.82 zone of inhibition values respectively against *K. pneumonia.* The MIC and MIB values of the molecule, **24** has found to be 0.25 and 0.5 against *S. aureus* and *K. pneumonia* respectively. Similarly, The MIC and MIB values of the molecule, **27** has found to be 0.25 and 0.5 against *S. aureus* and *K. pneumonia* respectively.

## Figures and Tables

**Table 1:** The structures of synthesised pyazolo[3,4-*b*]pyridine and triazole molecular hybrids.

**Class-I**	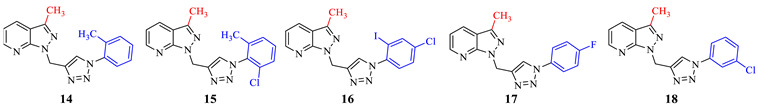
**Class-II**	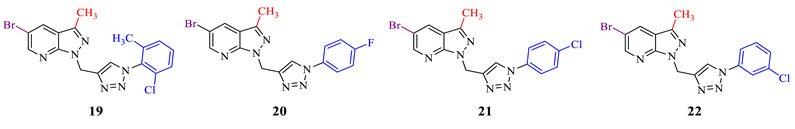
**Class-III**	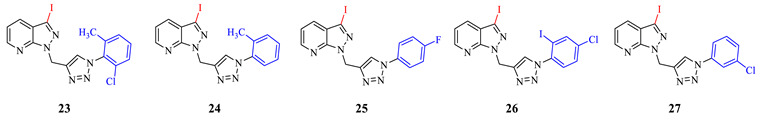
**Class-IV**	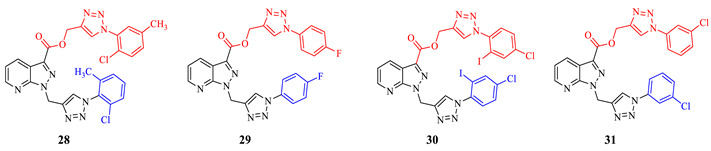

**Table 2:** The Physico-chemical and biological properties of pyazolo[3,4-*b*]pyridine and triazole molecular hybrids.

S. No.	Compound	Physico-Chemical Properties						In vitro studies					
								Zone of Inhibition		MIC and MBC			
		MW	iLog*P*	HA	HD	RB	TPSA	*S. aureus*	*K. pneumoniae*	*S. aureus*		*K. pneumoniae*	
										MIC	MBC	MIC	MBC
**1.**	**14**	304.35	2.87	4	0	3	61.42	NI	11 ± 0.15	0.25	0.5	0.25	0.5
2.	**15**	338.79	3.08	4	0	3	61.42	11 ± 0.12	13 ± 0.28	0.25	0.5	0.25	0.5
3.	**16**	450.66	3.04	4	0	3	61.42	15 ± 0.59	12 ± 0.22	0.25	0.5	0.25	0.5
4.	**17**	308.31	2.7	5	0	3	61.42	NI	11 ± 0.19	0.25	0.5	0.25	0.5
5.	**18**	324.77	2.95	4	0	3	61.42	NI	11 ± 0.09	0.5	1.0	0.5	1.0
6.	**19**	417.69	3.67	4	0	3	61.42	11 ± 0.17	13 ± 0.41	0.25	0.5	0.25	0.5
7.	**20**	387.21	3.18	5	0	3	61.42	NI	13 ± 0.39	0.25	0.5	0.25	0.5
8.	**21**	403.66	3.34	4	0	3	61.42	12 ± 0.19	NI	0.5	1.0	0.25	0.5
9.	**22**	403.66	3.3	4	0	3	61.42	11 ± 0.09	NI	0.5	1.0	0.5	1.0
10.	**23**	403.66	3.3	4	0	3	61.42	11 ± 0.16	10 ± 0.12	0.25	0.5	0.25	0.5
**11.**	**24**	416.22	2.87	4	0	3	61.42	15 ± 0.82	14 ± 0.75	0.25	0.5	0.25	0.5
12.	**25**	420.18	3.02	5	0	3	61.42	12 ± 0.18	15 ± 0.65	0.25	0.5	0.25	0.5
13.	**26**	562.53	3.26	4	0	3	61.42	13 ± 0.27	14 ± 0.45	0.25	0.5	0.25	0.5
**14.**	**27**	436.64	3.06	4	0	3	61.42	18 ± 0.95	16 ± 0.82	0.25	0.5	0.25	0.5
15.	**28**	574.42	4.63	8	0	8	118.43	10 ± 0.15	11 ± 0.15	0.5	1.0	0.25	0.5
16.	**29**	513.46	4.08	10	0	8	118.43	12 ± 0.21	14 ± 0.28	0.25	0.5	0.25	0.5
17.	**30**	798.16	4.71	8	0	8	118.43	10 ± 0.13	NI	0.25	0.5	0.25	0.5
18.	**31**	546.37	4.51	8	0	8	118.43	12 ± 0.19	NI	0.25	0.5	0.25	0.5
19.	**Ciprofloxacin**							32 ± 0.40	31 ± 0.20	0.062	0.125	0.031	0.062

NI: No Inhibition. Data are means (n = 3) ± Standard deviation of three replicates; Zone of inhibition (Diameter in mm) at a concentration of 100 μg; MIC and MBC data with a stock concentration of 1.0 mg/mL.

## Data Availability

The data presented in this study are available in insert article or Supplementary Materials here.

## Supplementary Materials

The following supporting information can be downloaded at: https://www.mdpi.com/article/10.3390/molecules27217647/s1. NMR spectrum, HPLC chromatogra, Mass spectrum and IR spectrum of compounds.

## Sample Availability

Samples of the compounds are not available from the authors.

## References

[1] Christaki E., Marcou M., Tofarides A. (2020). Antimicrobial Resistance in Bacteria: Mechanisms, Evolution, and Persistence. J. Mol. Evol..

[2] Septimus E.J. (2018). Antimicrobial Resistance: An Antimicrobial/Diagnostic Stewardship and Infection Prevention Approach. Med. Clin. N. Am..

[3] Pan American Health Organization (2022). 2021 Antibacterial Agents in Clinical and Preclinical Development: An Overview and Analysis. https://www.paho.org/en/documents/2021-antibacterial-agents-clinical-and-preclinical-development-overview-and-analysis.

[4] Maltezou H.C., Kontopidou F., Katerelos P., Daikos G., Roilides E., Theodoridou M. (2013). Infections Caused by Carbapenem-resistant Gram-negative Pathogens in Hospitalized Children. Pediatr. Infect. Dis. J..

[5] Tumbarello M., Viale P., Viscoli C., Trecarichi E.M., Tumietto F., Marchese A., Spanu T., Ambretti S., Ginocchio F., Cristini F. (2012). Predictors of Mortality in Bloodstream Infections Caused by Klebsiella pneumoniae Carbapenemase–Producing K. pneumoniae: Importance of Combination Therapy. Clin. Infect. Dis..

[6] Hobson Claire A., Pierrat G., Tenaillon O., Bonacorsi S., Bercot B., Jaouen E., Jacquier H., Birgy A. (2022). Klebsiella pneumoniae Carbapenemase Variants Resistant to Ceftazidime-Avibactam: An Evolutionary Overview. Antimicrob. Agents Chemother..

[7] Tabah A., Laupland K.B. (2022). Update on Staphylococcus aureus bacteraemia. Curr. Opin. Crit. Care.

[8] Nourollahpour Shiadeh M., Sepidarkish M., Mollalo A., As’adi N., Khani S., Shahhosseini Z., Danesh M., Esfandyari S., Mokdad A.H., Rostami A. (2022). Worldwide prevalence of maternal methicillin-resistant Staphylococcus aureus colonization: A systematic review and meta-analysis. Microb. Pathog..

[9] Mlynarczyk-Bonikowska B., Kowalewski C., Krolak-Ulinska A., Marusza W. (2022). Molecular Mechanisms of Drug Resistance in Staphylococcus aureus. Int. J. Mol. Sci..

[10] Tavares T.D., Antunes J.C., Padrão J., Ribeiro A.I., Zille A., Amorim M.T.P., Ferreira F., Felgueiras H.P. (2020). Activity of Specialized Biomolecules against Gram-Positive and Gram-Negative Bacteria. Antibiotics.

[11] Donaire-Arias A., Montagut A.M., Puig de la Bellacasa R., Estrada-Tejedor R., Teixidó J., Borrell J.I. (2022). 1H-Pyazolo[3,4-b]pyridines: Synthesis and Biomedical Applications. Molecules.

[12] Wenglowsky S. (2013). Pyazolo[3,4-b]pyridine kinase inhibitors: A patent review (2008–present). Expert Opin. Ther. Pat..

[13] Bozorov K., Zhao J., Aisa H.A. (2019). 1,2,3-Triazole-containing hybrids as leads in medicinal chemistry: A recent overview. Bioorg. Med. Chem..

[14] Zhang B. (2019). Comprehensive review on the anti-bacterial activity of 1,2,3-triazole hybrids. Eur. J. Med. Chem..

[15] Kaur J., Saxena M., Rishi N. (2021). An Overview of Recent Advances in Biomedical Applications of Click Chemistry. Bioconjug. Chem..

[16] Xu Z. (2020). 1,2,3-Triazole-containing hybrids with potential antibacterial activity against methicillin-resistant Staphylococcus aureus (MRSA). Eur. J. Med. Chem..

[17] Tan Z., Deng J., Ye Q., Zhang Z. (2021). Triazole-containing hybrids with anti-Mycobacterium tuberculosis potential—Part I: 1,2,3-Triazole. Future Med. Chem..

[18] Li J., Zhang J. (2022). The Antibacterial Activity of 1,2,3-triazole- and 1,2,4-Triazole-containing Hybrids against Staphylococcus aureus: An Updated Review (2020- Present). Curr. Top. Med. Chem..

[19] Upadhyay C.H. (2021). Coumarin-1,2,3-triazole Hybrid Molecules: An Emerging Scaffold for Combating Drug Resistance. Curr. Top. Med. Chem..

[20] Hublikar M., Kadu V., Dublad J.K., Raut D., Shirame S., Makam P., Bhosale R. (2020). (E)-2-(2-Allylidenehydrazinyl) thiazole derivatives: Design, green synthesis, in silico and in vitro antimycobacterial and radical scavenging studies. Arch. Pharm..

[21] Parameshwar M., Ramkishore M. (2021). “Big Three” Infectious Diseases: Tuberculosis, Malaria and HIV/AIDS. Curr. Top. Med. Chem..

[22] Anaikutti P., Selvaraj M., Prabhakaran J., Pooventhiran T., Jeyakumar T.C., Thomas R., Makam P. (2022). Indolyl-4H-chromenes: Multicomponent one-pot green synthesis, in vitro and in silico, anticancer and antioxidant studies. J. Mol. Struct..

[23] Hublikar M., Kadu V., Raut D., Shirame S., Anbarasu S., Al-Muhanna M.K., Makam P., Bhosale R. (2022). 3-Substituted-2-oxindole derivatives: Design, synthesis and their anti-tuberculosis and radical scavenging dual-action studies. J. Mol. Struct..

[24] Matsa R., Makam P., Sethi G., Thottasseri A.A., Kizhakkandiyil A.R., Ramadas K., Mariappan V., Pillai A.B., Kannan T. (2022). Pyridine appended 2-hydrazinylthiazole derivatives: Design, synthesis, in vitro and in silico antimycobacterial studies. RSC Adv..

[25] Huuskonen J., Livingstone D.J., Manallack D.T. (2008). Prediction of drug solubility from molecular structure using a drug-like training set. SAR QSAR Environ. Res..

[26] Yazdanian M., Glynn S.L., Wright J.L., Hawi A. (1998). Correlating Partitioning and Caco-2 Cell Permeability of Structurally Diverse Small Molecular Weight Compounds. Pharm. Res..

[27] Artursson P., Ungell A.-L., Löfroth J.-E. (1993). Selective Paracellular Permeability in Two Models of Intestinal Absorption: Cultured Monolayers of Human Intestinal Epithelial Cells and Rat Intestinal Segments. Pharm. Res..

[28] Sharifi M., Ghafourian T. (2014). Estimation of Biliary Excretion of Foreign Compounds Using Properties of Molecular Structure. AAPS J..

[29] Varma M.V., Lai Y., El-Kattan A.F. (2017). Molecular properties associated with transporter-mediated drug disposition. Adv. Drug Deliv. Rev..

[30] Struck S., Schmidt U., Gruening B., Jaeger I.S., Hossbach J., Preissner R. (2008). Toxicity versus potency: Elucidation of toxicity properties discriminating between toxins, drugs, and natural compounds. Genome Informatics 2008.

[31] Lipinski C.A., Lombardo F., Dominy B.W., Feeney P.J. (1997). Experimental and computational approaches to estimate solubility and permeability in drug discovery and development settings. Adv. Drug Deliv. Rev..

[32] Lipinski C.A., Lombardo F., Dominy B.W., Feeney P.J. (2001). Experimental and computational approaches to estimate solubility and permeability in drug discovery and development settings. Adv. Drug Deliv. Rev..

[33] Kola I., Landis J. (2004). Can the pharmaceutical industry reduce attrition rates?. Nat. Rev. Drug Discov..

[34] Veber D.F., Johnson S.R., Cheng H.-Y., Smith B.R., Ward K.W., Kopple K.D. (2002). Molecular properties that influence the oral bioavailability of drug candidates. J. Med. Chem..

[35] Leeson P.D., Davis A.M. (2004). Time-related differences in the physical property profiles of oral drugs. J. Med. Chem..

[36] Gleeson M.P., Hersey A., Montanari D., Overington J. (2011). Probing the links between in vitro potency, ADMET and physicochemical parameters. Nat. Rev. Drug Discov..

[37] Khojasteh S., Wong H., Hop C.C.A. (2011). ADME properties and their dependence on physicochemical properties. Drug Metabolism and Pharmacokinetics Quick Guide.

[38] Daina A., Michielin O., Zoete V. (2017). SwissADME: A free web tool to evaluate pharmacokinetics, drug-likeness and medicinal chemistry friendliness of small molecules. Sci. Rep..

[39] Gaywood A.P., McNab H. (2010). 3-Hydroxypyrrolo [2,3-b]pyridine and related compounds—Indoxyl analogues with fused electron deficient rings. Org. Biomol. Chem..

[40] Moir M., Lane S., Lai F., Connor M., Hibbs D.E., Kassiou M. (2019). Strategies to develop selective CB2 receptor agonists from indole carboxamide synthetic cannabinoids. Eur. J. Med. Chem..

[41] Stepanenko I.N., Novak M.S., Mühlgassner G., Roller A., Hejl M., Arion V.B., Jakupec M.A., Keppler B.K. (2011). Organometallic 3-(1H-Benzimidazol-2-yl)-1H-pyazolo[3,4-b]pyridines as Potential Anticancer Agents. Inorg. Chem..

[42] Ye Q., Cao J., Zhou X., Lv D., He Q., Yang B., Hu Y. (2009). Synthesis and evaluation of novel 7-azaindazolyl-indolyl-maleimide derivatives as antitumor agents and protein kinase C inhibitors. Bioorg. Med. Chem..

[43] Huang P.-H., Wen Y.-S., Shen J.-Y. (2014). 3-Iodo-1H-pyazolo[3,4-b]pyridine. Acta Crystallogr. Sect. E.

[44] Nagender P., Malla Reddy G., Naresh Kumar R., Poornachandra Y., Ganesh Kumar C., Narsaiah B. (2014). Synthesis, cytotoxicity, antimicrobial and anti-biofilm activities of novel pyazolo[3,4-b]pyridine and pyrimidine functionalized 1,2,3-triazole derivatives. Bioorg. Med. Chem. Lett..

[45] Nagender P., Naresh Kumar R., Malla Reddy G., Krishna Swaroop D., Poornachandra Y., Ganesh Kumar C., Narsaiah B. (2016). Synthesis of novel hydrazone and azole functionalized pyazolo[3,4-b]pyridine derivatives as promising anticancer agents. Bioorg. Med. Chem. Lett..

