# Supplementary material for: Molecular Hybrids of Pyazolo[3,4-b]pyridine and Triazole: Design, Synthesis and In Vitro Antibacterial Studies
Source: Molecules. 2022 Nov 7;27(21):7647. doi: 10.3390/molecules27217647 (PMC9655101; doi:10.3390/molecules27217647)
Supplement: Supplementary file 1 [file molecules-27-07647-s001.zip › molecules-1911818-supplementary.pdf]

Supporting Information

Molecular Hybrids of Pyazolo[3,4-*b*]pyridine and Triazole: Design, Synthesis and In Vitro Antibacterial Studies

Narasimha Rao Bandaru [1], Parameshwar Makam [2], Parameswari Akshinthala [3], Naresh Kumar Katari [1], Venkanna Banothu [4], Balakrishna Kolli [1,5,*] and Rambabu Gundla [1,*]

[1] Department of Chemistry, GITAM School of Science, GITAM Deemed to be University Hyderabad, Telangana 502329, India
[2] Department of Chemistry, School of Applied and Life Sciences, Uttaranchal University, Arcadia Grant, Chandanwari, Premnagar, Dehradun, Uttarakhand 248007, India
[3] Department of Science and Humanities, MLR Institute of Technology, Dundigal, Medchal, Hyderabad, Telangana 500043, India
[4] Department of Biotechnology, University Post Graduate College of Science and Technology, Jawaharlal Nehru Technological University Hyderabad, Telangana 500085, India
[5] Department of Chemistry, GITAM School of Science, GITAM Deemed to be University Visakhapatnam, Andhra Pradesh 530045, India
* Correspondence: bkolli@gitam.edu (B.K.); rgundla@gitam.edu (R.G.)

Table of Contents

S. No.	Name
01	^{1}H NMR spectrum of *3-Methyl-1-((1-(o-tolyl)-1H-1,2,3-triazol-4-yl)methyl)-1H-pyrazolo[3,4-b]pyridine* (14)
02	^{13}C NMR spectrum of *3-Methyl-1-((1-(o-tolyl)-1H-1,2,3-triazol-4-yl)methyl)-1H-pyrazolo[3,4-b]pyridine* (14)
03	HPLC chromatogram of *3-Methyl-1-((1-(o-tolyl)-1H-1,2,3-triazol-4-yl)methyl)-1H-pyrazolo[3,4-b]pyridine* (14)
04	^{1}H NMR spectrum of *1-((1-(2-Chloro-6-methylphenyl)-1H-1,2,3-triazol-4-yl)methyl)-3-methyl-1H-pyrazolo[3,4-b]pyridine* (15)
05	^{13}C NMR spectrum of *1-((1-(2-Chloro-6-methylphenyl)-1H-1,2,3-triazol-4-yl)methyl)-3-methyl-1H-pyrazolo[3,4-b]pyridine* (15)
06	Mass spectrum of *1-((1-(2-Chloro-6-methylphenyl)-1H-1,2,3-triazol-4-yl)methyl)-3-methyl-1H-pyrazolo[3,4-b]pyridine* (15)
07	HPLC chromatogram of *1-((1-(2-Chloro-6-methylphenyl)-1H-1,2,3-triazol-4-yl)methyl)-3-methyl-1H-pyrazolo[3,4-b]pyridine* (15)
08	^{1}H NMR spectrum of *1-((1-(4-Chloro-2-iodophenyl)-1H-1,2,3-triazol-4-yl)methyl)-3-methyl-1H-pyrazolo[3,4-b]pyridine* (16)

09	^{13}C NMR spectrum of *1-((1-(4-Chloro-2-iodophenyl)-1H-1,2,3-triazol-4-yl)methyl)-3-methyl-1H-pyrazolo[3,4-b]pyridine* (16)
10	IR spectrum of *1-((1-(4-Chloro-2-iodophenyl)-1H-1,2,3-triazol-4-yl)methyl)-3-methyl-1H-pyrazolo[3,4-b]pyridine* (16)
11	Mass spectrum of *1-((1-(4-Chloro-2-iodophenyl)-1H-1,2,3-triazol-4-yl)methyl)-3-methyl-1H-pyrazolo[3,4-b]pyridine* (16)
12	HPLC chromatogram of *1-((1-(4-Chloro-2-iodophenyl)-1H-1,2,3-triazol-4-yl)methyl)-3-methyl-1H-pyrazolo[3,4-b]pyridine* (16)
13	^{1}H NMR spectrum of *1-((1-(4-Fluorophenyl)-1H-1,2,3-triazol-4-yl)methyl)-3-methyl-1H-pyrazolo[3,4-b]pyridine* (17)
14	^{13}C NMR spectrum of *1-((1-(4-Fluorophenyl)-1H-1,2,3-triazol-4-yl)methyl)-3-methyl-1H-pyrazolo[3,4-b]pyridine* (17)
15	IR spectrum of *1-((1-(4-Fluorophenyl)-1H-1,2,3-triazol-4-yl)methyl)-3-methyl-1H-pyrazolo[3,4-b]pyridine* (17)
16	Mass spectrum of *1-((1-(4-Fluorophenyl)-1H-1,2,3-triazol-4-yl)methyl)-3-methyl-1H-pyrazolo[3,4-b]pyridine* (17)
17	HPLC chromatogram of *1-((1-(4-Fluorophenyl)-1H-1,2,3-triazol-4-yl)methyl)-3-methyl-1H-pyrazolo[3,4-b]pyridine* (17)
18	^{1}H NMR spectrum of *1-((1-(3-Chlorophenyl)-1H-1,2,3-triazol-4-yl)methyl)-3-methyl-1H-pyrazolo[3,4-b]pyridine* (18)
19	^{13}C NMR spectrum of *1-((1-(3-Chlorophenyl)-1H-1,2,3-triazol-4-yl)methyl)-3-methyl-1H-pyrazolo[3,4-b]pyridine* (18)
20	Mass spectrum of *1-((1-(3-Chlorophenyl)-1H-1,2,3-triazol-4-yl)methyl)-3-methyl-1H-pyrazolo[3,4-b]pyridine* (18)
21	HPLC chromatogram of *1-((1-(3-Chlorophenyl)-1H-1,2,3-triazol-4-yl)methyl)-3-methyl-1H-pyrazolo[3,4-b]pyridine* (18)
22	^{1}H NMR spectrum of *5-Bromo-1-((1-(2-chloro-6-methylphenyl)-1H-1,2,3-triazol-4-yl)methyl)-3-methyl-1H-pyrazolo[3,4-b]pyridine* (19)
23	^{13}C NMR spectrum of *5-Bromo-1-((1-(2-chloro-6-methylphenyl)-1H-1,2,3-triazol-4-yl)methyl)-3-methyl-1H-pyrazolo[3,4-b]pyridine* (19)
24	IR spectrum of *5-Bromo-1-((1-(2-chloro-6-methylphenyl)-1H-1,2,3-triazol-4-yl)methyl)-3-methyl-1H-pyrazolo[3,4-b]pyridine* (19)
25	Mass spectrum of *5-Bromo-1-((1-(2-chloro-6-methylphenyl)-1H-1,2,3-triazol-4-yl)methyl)-3-methyl-1H-pyrazolo[3,4-b]pyridine* (19)
26	HPLC chromatogram of *5-Bromo-1-((1-(2-chloro-6-methylphenyl)-1H-1,2,3-triazol-4-yl)methyl)-3-methyl-1H-pyrazolo[3,4-b]pyridine* (19)
27	^{1}H NMR spectrum of *5-Bromo-1-((1-(4-fluorophenyl)-1H-1,2,3-triazol-4-yl)methyl)-3-methyl-1H-*

	pyrazolo[3,4-b]pyridine (20)
28	^{13}C NMR spectrum of *5-Bromo-1-((1-(4-fluorophenyl)-1H-1,2,3-triazol-4-yl)methyl)-3-methyl-1H-pyrazolo[3,4-b]pyridine* (20)
29	IR spectrum of *5-Bromo-1-((1-(4-fluorophenyl)-1H-1,2,3-triazol-4-yl)methyl)-3-methyl-1H-pyrazolo[3,4-b]pyridine* (20)
30	Mass spectrum of *5-Bromo-1-((1-(4-fluorophenyl)-1H-1,2,3-triazol-4-yl)methyl)-3-methyl-1H-pyrazolo[3,4-b]pyridine* (20)
31	HPLC chromatogram of *5-Bromo-1-((1-(4-fluorophenyl)-1H-1,2,3-triazol-4-yl)methyl)-3-methyl-1H-pyrazolo[3,4-b]pyridine* (20)
32	^{1}H NMR spectrum of *5-Bromo-1-((1-(4-chlorophenyl)-1H-1,2,3-triazol-4-yl)methyl)-3-methyl-1H-pyrazolo[3,4-b]pyridine* (21)
33	^{13}C NMR spectrum of *5-Bromo-1-((1-(4-chlorophenyl)-1H-1,2,3-triazol-4-yl)methyl)-3-methyl-1H-pyrazolo[3,4-b]pyridine* (21)
34	IR spectrum of *5-Bromo-1-((1-(4-chlorophenyl)-1H-1,2,3-triazol-4-yl)methyl)-3-methyl-1H-pyrazolo[3,4-b]pyridine* (21)
35	Mass spectrum of *5-Bromo-1-((1-(4-chlorophenyl)-1H-1,2,3-triazol-4-yl)methyl)-3-methyl-1H-pyrazolo[3,4-b]pyridine* (21)
36	HPLC chromatogram of *5-Bromo-1-((1-(4-chlorophenyl)-1H-1,2,3-triazol-4-yl)methyl)-3-methyl-1H-pyrazolo[3,4-b]pyridine* (21)
37	^{1}H NMR spectrum of *5-Bromo-1-((1-(3-chlorophenyl)-1H-1,2,3-triazol-4-yl)methyl)-3-methyl-1H-pyrazolo[3,4-b]pyridine* (22)
38	^{13}C NMR spectrum of *5-Bromo-1-((1-(3-chlorophenyl)-1H-1,2,3-triazol-4-yl)methyl)-3-methyl-1H-pyrazolo[3,4-b]pyridine* (22)
39	IR spectrum of *5-Bromo-1-((1-(3-chlorophenyl)-1H-1,2,3-triazol-4-yl)methyl)-3-methyl-1H-pyrazolo[3,4-b]pyridine* (22)
40	Mass spectrum of *5-Bromo-1-((1-(3-chlorophenyl)-1H-1,2,3-triazol-4-yl)methyl)-3-methyl-1H-pyrazolo[3,4-b]pyridine* (22)
41	HPLC chromatogram of *5-Bromo-1-((1-(3-chlorophenyl)-1H-1,2,3-triazol-4-yl)methyl)-3-methyl-1H-pyrazolo[3,4-b]pyridine* (22)
42	^{1}H NMR spectrum of *1-((1-(2-Chloro-6-methylphenyl)-1H-1,2,3-triazol-4-yl)methyl)-3-iodo-1H-pyrazolo[3,4-b]pyridine* (23)
43	^{13}C NMR spectrum of *1-((1-(2-Chloro-6-methylphenyl)-1H-1,2,3-triazol-4-yl)methyl)-3-iodo-1H-pyrazolo[3,4-b]pyridine* (23)
44	IR spectrum of *1-((1-(2-Chloro-6-methylphenyl)-1H-1,2,3-triazol-4-yl)methyl)-3-iodo-1H-pyrazolo[3,4-b]pyridine* (23)
45	Mass spectrum of *1-((1-(2-Chloro-6-methylphenyl)-1H-1,2,3-triazol-4-yl)methyl)-3-iodo-1H-pyrazolo[3,4-b]pyridine* (23)

46	HPLC chromatogram of *1-((1-(2-Chloro-6-methylphenyl)-1H-1,2,3-triazol-4-yl)methyl)-3-iodo-1H-pyrazolo[3,4-b]pyridine* (23)
47	^{1}H NMR spectrum of *3-Iodo-1-((1-(o-tolyl)-1H-1,2,3-triazol-4-yl)methyl)-1H-pyrazolo[3,4-b]pyridine* (24)
48	^{13}C NMR spectrum of *3-Iodo-1-((1-(o-tolyl)-1H-1,2,3-triazol-4-yl)methyl)-1H-pyrazolo[3,4-b]pyridine* (24)
49	IR spectrum of *3-Iodo-1-((1-(o-tolyl)-1H-1,2,3-triazol-4-yl)methyl)-1H-pyrazolo[3,4-b]pyridine* (24)
50	Mass spectrum of *3-Iodo-1-((1-(o-tolyl)-1H-1,2,3-triazol-4-yl)methyl)-1H-pyrazolo[3,4-b]pyridine* (24)
51	HPLC chromatogram of *3-Iodo-1-((1-(o-tolyl)-1H-1,2,3-triazol-4-yl)methyl)-1H-pyrazolo[3,4-b]pyridine* (24)
52	^{1}H NMR spectrum of *1-((1-(4-Fluorophenyl)-1H-1,2,3-triazol-4-yl)methyl)-3-iodo-1H-pyrazolo[3,4-b]pyridin* (25)
53	^{13}C NMR spectrum of *1-((1-(4-Fluorophenyl)-1H-1,2,3-triazol-4-yl)methyl)-3-iodo-1H-pyrazolo[3,4-b]pyridin* (25)
54	IR spectrum of *1-((1-(4-Fluorophenyl)-1H-1,2,3-triazol-4-yl)methyl)-3-iodo-1H-pyrazolo[3,4-b]pyridin* (25)
55	Mass spectrum of *1-((1-(4-Fluorophenyl)-1H-1,2,3-triazol-4-yl)methyl)-3-iodo-1H-pyrazolo[3,4-b]pyridin* (25)
56	HPLC chromatogram of *1-((1-(4-Fluorophenyl)-1H-1,2,3-triazol-4-yl)methyl)-3-iodo-1H-pyrazolo[3,4-b]pyridin* (25)
57	^{1}H NMR spectrum of *1-((1-(4-Chloro-2-iodophenyl)-1H-1,2,3-triazol-4-yl)methyl)-3-iodo-1H-pyrazolo[3,4-b]pyridine* (26)
58	^{13}C NMR spectrum of *1-((1-(4-Chloro-2-iodophenyl)-1H-1,2,3-triazol-4-yl)methyl)-3-iodo-1H-pyrazolo[3,4-b]pyridine* (26)
59	IR spectrum of *1-((1-(4-Chloro-2-iodophenyl)-1H-1,2,3-triazol-4-yl)methyl)-3-iodo-1H-pyrazolo[3,4-b]pyridine* (26)
60	Mass spectrum of *1-((1-(4-Chloro-2-iodophenyl)-1H-1,2,3-triazol-4-yl)methyl)-3-iodo-1H-pyrazolo[3,4-b]pyridine* (26)
61	HPLC chromatogram of *1-((1-(4-Chloro-2-iodophenyl)-1H-1,2,3-triazol-4-yl)methyl)-3-iodo-1H-pyrazolo[3,4-b]pyridine* (26)
62	^{1}H NMR spectrum of *1-((1-(3-Chlorophenyl)-1H-1,2,3-triazol-4-yl)methyl)-3-iodo-1H-pyrazolo[3,4-b]pyridine* (27)
63	^{13}C NMR spectrum of *1-((1-(3-Chlorophenyl)-1H-1,2,3-triazol-4-yl)methyl)-3-iodo-1H-pyrazolo[3,4-b]pyridine* (27)
64	HPLC chromatogram of *1-((1-(3-Chlorophenyl)-1H-1,2,3-triazol-4-yl)methyl)-3-iodo-1H-pyrazolo[3,4-b]pyridine* (27)

65	^{1}H NMR spectrum of *(1-(2-Chloro-5-methylphenyl)-1H-1,2,3-triazol-4-yl)methyl1-((1-(2-chloro-6-methylphenyl) -1H-1,2,3-triazol-4-yl)methyl)-1H-pyrazolo[3,4-b]pyridine-3-carboxylate* (28)
66	^{13}C NMR spectrum of *(1-(2-Chloro-5-methylphenyl)-1H-1,2,3-triazol-4-yl)methyl1-((1-(2-chloro-6-methylphenyl) -1H-1,2,3-triazol-4-yl)methyl)-1H-pyrazolo[3,4-b]pyridine-3-carboxylate* (28)
67	IR spectrum of *(1-(2-Chloro-5-methylphenyl)-1H-1,2,3-triazol-4-yl)methyl1-((1-(2-chloro-6-methylphenyl) -1H-1,2,3-triazol-4-yl)methyl)-1H-pyrazolo[3,4-b]pyridine-3-carboxylate* (28)
68	Mass spectrum of *(1-(2-Chloro-5-methylphenyl)-1H-1,2,3-triazol-4-yl)methyl1-((1-(2-chloro-6-methylphenyl) -1H-1,2,3-triazol-4-yl)methyl)-1H-pyrazolo[3,4-b]pyridine-3-carboxylate* (28)
69	HPLC chromatogram of *(1-(2-Chloro-5-methylphenyl)-1H-1,2,3-triazol-4-yl)methyl1-((1-(2-chloro-6-methylphenyl) -1H-1,2,3-triazol-4-yl)methyl)-1H-pyrazolo[3,4-b]pyridine-3-carboxylate* (28)
70	^{1}H NMR spectrum of *(1-(4-Fluorophenyl)-1H-1,2,3-triazol-4-yl)methyl 1-((1-(4-fluorophenyl)-1H-1,2,3-triazol-4-yl)methyl)-1H-pyrazolo[3,4-b]pyridine-3-carboxylate* (29)
71	^{13}C NMR spectrum of *(1-(4-Fluorophenyl)-1H-1,2,3-triazol-4-yl)methyl 1-((1-(4-fluorophenyl)-1H-1,2,3-triazol-4-yl)methyl)-1H-pyrazolo[3,4-b]pyridine-3-carboxylate* (29)
72	IR spectrum of *(1-(4-Fluorophenyl)-1H-1,2,3-triazol-4-yl)methyl 1-((1-(4-fluorophenyl)-1H-1,2,3-triazol-4-yl)methyl)-1H-pyrazolo[3,4-b]pyridine-3-carboxylate* (29)
73	Mass spectrum of *(1-(4-Fluorophenyl)-1H-1,2,3-triazol-4-yl)methyl 1-((1-(4-fluorophenyl)-1H-1,2,3-triazol-4-yl)methyl)-1H-pyrazolo[3,4-b]pyridine-3-carboxylate* (29)
74	HPLC chromatogram of *(1-(4-Fluorophenyl)-1H-1,2,3-triazol-4-yl)methyl 1-((1-(4-fluorophenyl)-1H-1,2,3-triazol-4-yl)methyl)-1H-pyrazolo[3,4-b]pyridine-3-carboxylate* (29)
75	^{1}H NMR spectrum of *(1-(4-Chloro-2-iodophenyl)-1H-1,2,3-triazol-4-yl)methyl 1-((1-(4-chloro-2-iodophenyl)-1H-1,2,3-triazol-4-yl)methyl)-1H-pyrazolo[3,4-b]pyridine-3-carboxylate* (30)
76	^{13}C NMR spectrum of *(1-(4-Chloro-2-iodophenyl)-1H-1,2,3-triazol-4-yl)methyl 1-((1-(4-chloro-2-iodophenyl)-1H-1,2,3-triazol-4-yl)methyl)-1H-pyrazolo[3,4-b]pyridine-3-carboxylate* (30)
77	IR spectrum of *(1-(4-Chloro-2-iodophenyl)-1H-1,2,3-triazol-4-yl)methyl 1-((1-(4-chloro-2-iodophenyl)-1H-1,2,3-triazol-4-yl)methyl)-1H-pyrazolo[3,4-b]pyridine-3-carboxylate* (30)
78	Mass spectrum of *(1-(4-Chloro-2-iodophenyl)-1H-1,2,3-triazol-4-yl)methyl 1-((1-(4-chloro-2-iodophenyl)-1H-1,2,3-triazol-4-yl)methyl)-1H-pyrazolo[3,4-b]pyridine-3-carboxylate* (30)
79	HPLC chromatogram of *(1-(4-Chloro-2-iodophenyl)-1H-1,2,3-triazol-4-yl)methyl 1-((1-(4-chloro-2-iodophenyl)-1H-1,2,3-triazol-4-yl)methyl)-1H-pyrazolo[3,4-b]pyridine-3-carboxylate* (30)
80	^{1}H NMR spectrum of *(1-(3-Chlorophenyl)-1H-1,2,3-triazol-4-yl)methyl 1-((1-(3-chlorophenyl)-1H-1,2,3-triazol-4-yl)methyl)-1H-pyrazolo[3,4-b]pyridine-3-carboxylate* (31)
81	^{13}C NMR spectrum of *(1-(3-Chlorophenyl)-1H-1,2,3-triazol-4-yl)methyl 1-((1-(3-chlorophenyl)-1H-1,2,3-triazol-4-yl)methyl)-1H-pyrazolo[3,4-b]pyridine-3-carboxylate* (31)
82	IR spectrum of *(1-(3-Chlorophenyl)-1H-1,2,3-triazol-4-yl)methyl 1-((1-(3-chlorophenyl)-1H-1,2,3-triazol-4-yl)methyl)-1H-pyrazolo[3,4-b]pyridine-3-carboxylate* (31)

83	Mass spectrum of *(1-(3-Chlorophenyl)-1H-1,2,3-triazol-4-yl)methyl 1-((1-(3-chlorophenyl)-1H-1,2,3-triazol-4-yl)methyl)-1H-pyrazolo[3,4-b]pyridine-3-carboxylate* (31)
84	HPLC chromatogram of *(1-(3-Chlorophenyl)-1H-1,2,3-triazol-4-yl)methyl 1-((1-(3-chlorophenyl)-1H-1,2,3-triazol-4-yl)methyl)-1H-pyrazolo[3,4-b]pyridine-3-carboxylate* (31)

Spectra:

^{1}H NMR spectrum of *3-Methyl-1-((1-(o-tolyl)-1H-1,2,3-triazol-4-yl)methyl)-1H-pyrazolo[3,4-b]pyridine* (14)

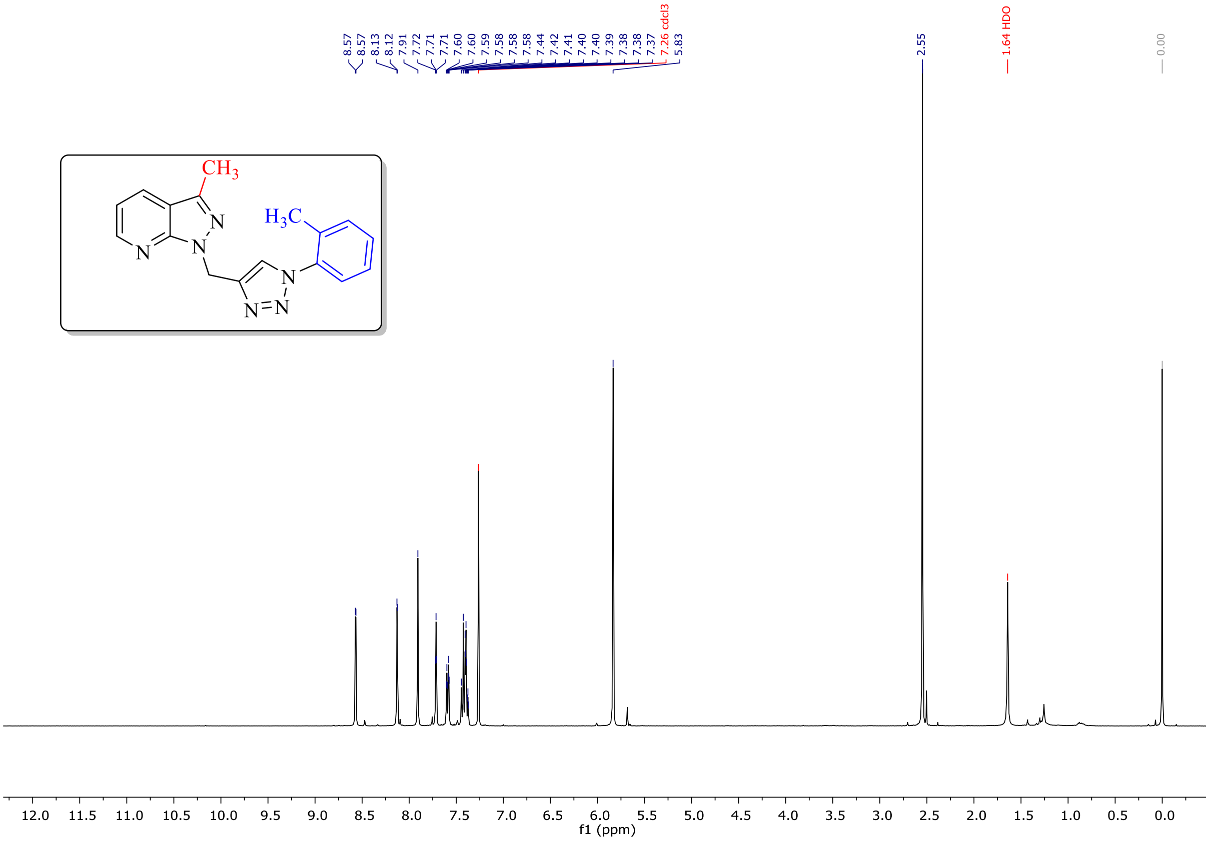

^{13}C NMR spectrum of *3-Methyl-1-((1-(o-tolyl)-1H-1,2,3-triazol-4-yl)methyl)-1H-pyrazolo[3,4-b]pyridine* (14)

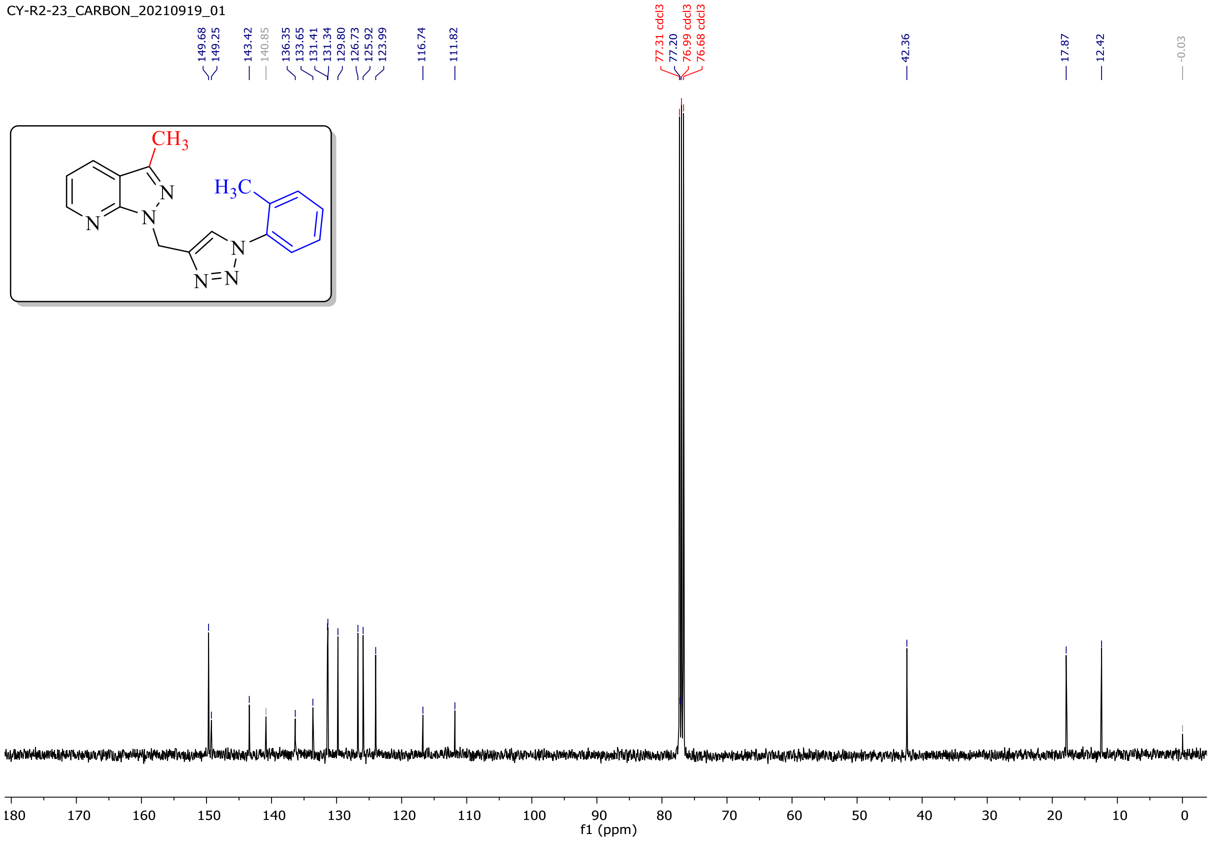

HPLC chromatogram of *3-Methyl-1-((1-(o-tolyl)-1H-1,2,3-triazol-4-yl)methyl)-1H-pyrazolo[3,4-b]pyridine* (14)

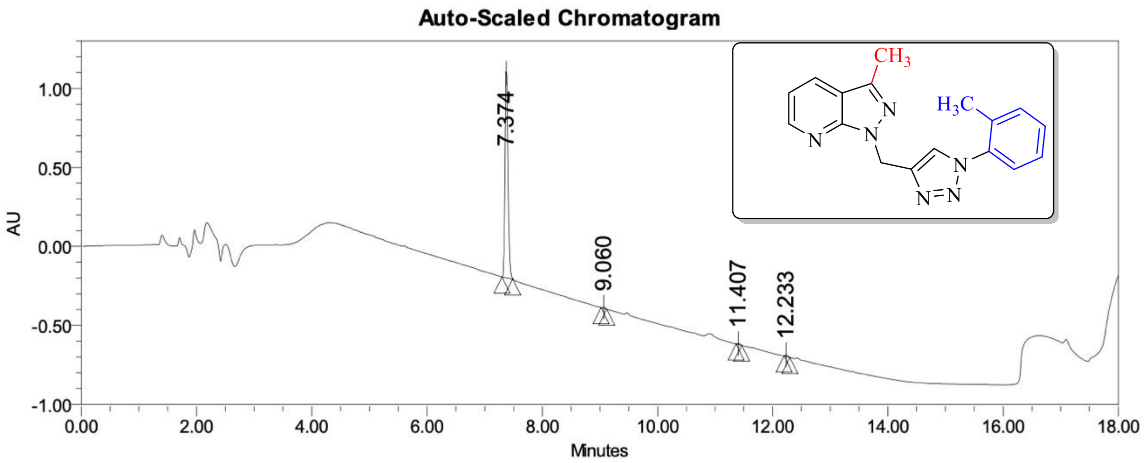

Peak Results

	Name	RT	Area	% Area
1		7.374	4724530	98.36
2		9.060	26208	0.55
3		11.407	19985	0.42
4		12.233	32574	0.68

^{1}H NMR spectrum of *1-((1-(2-Chloro-6-methylphenyl)-1H-1,2,3-triazol-4-yl)methyl)-3-methyl-1H-pyrazolo[3,4-b]pyridine* (15)

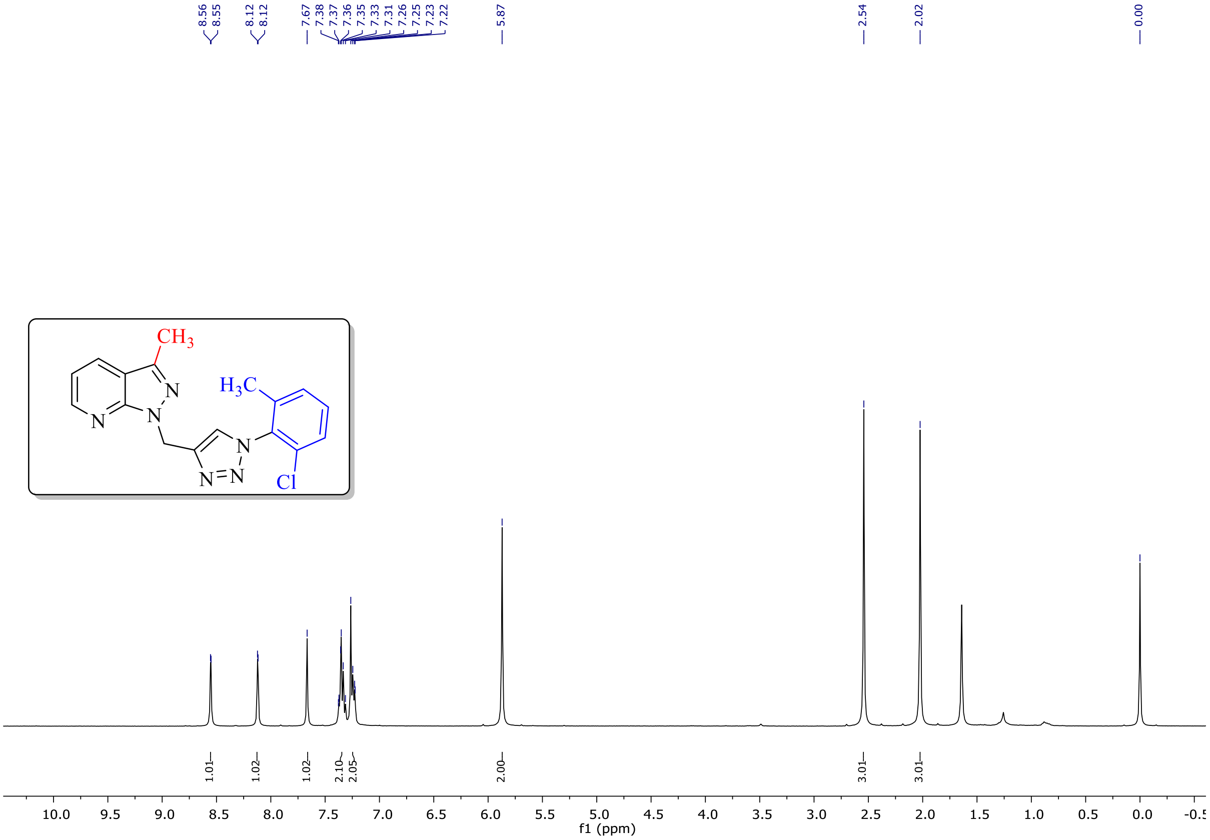

^{13}C NMR spectrum of *1-((1-(2-Chloro-6-methylphenyl)-1H-1,2,3-triazol-4-yl)methyl)-3-methyl-1H-pyrazolo[3,4-b]pyridine* (15)

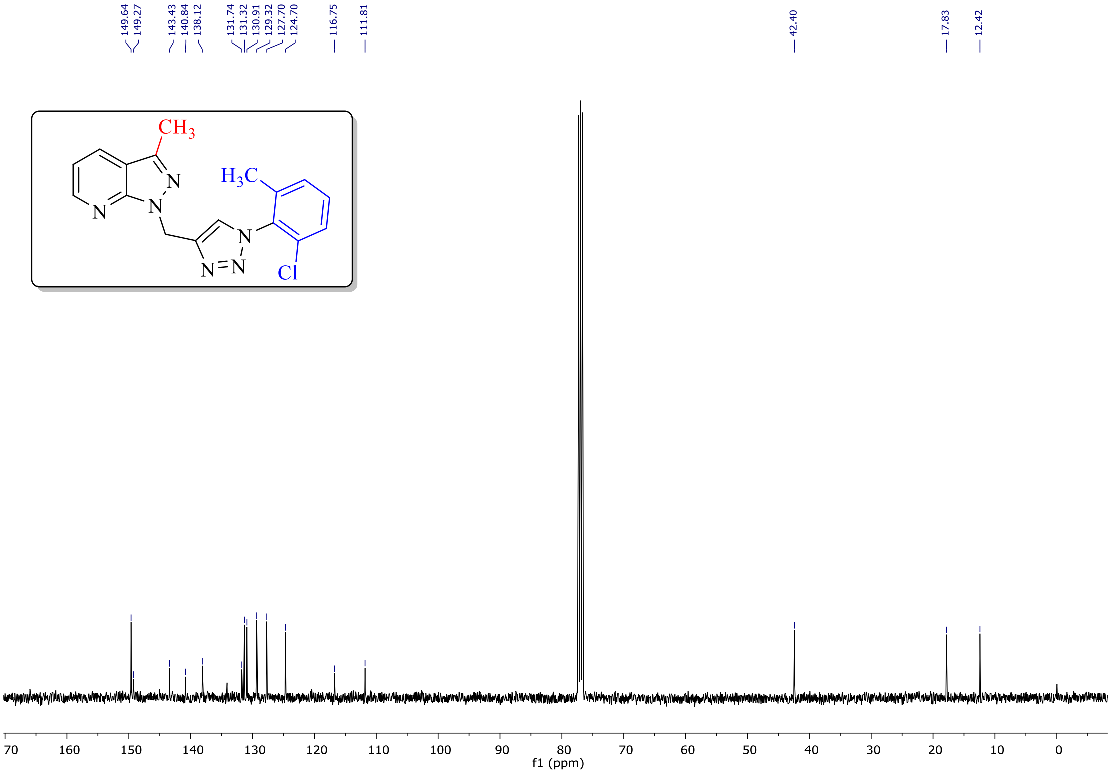

Mass spectrum of *1-((1-(2-Chloro-6-methylphenyl)-1H-1,2,3-triazol-4-yl)methyl)-3-methyl-1H-pyrazolo[3,4-b]pyridine* (15)

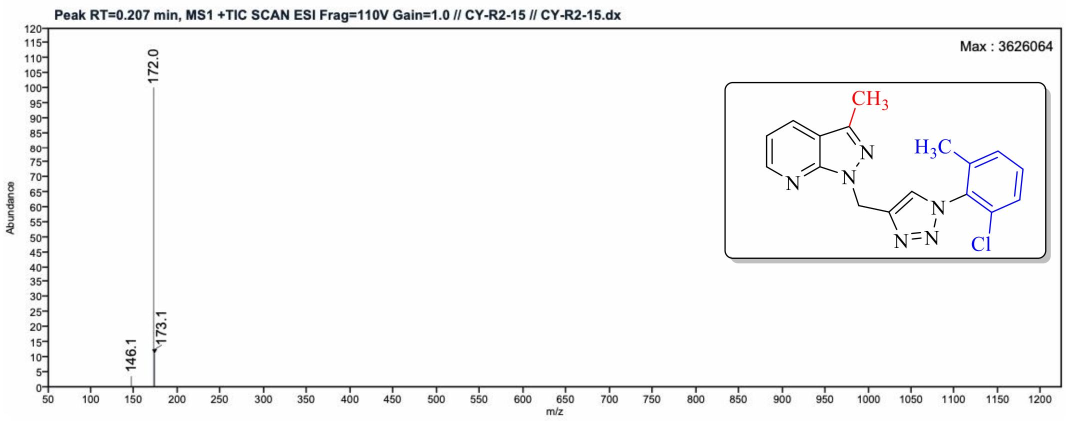

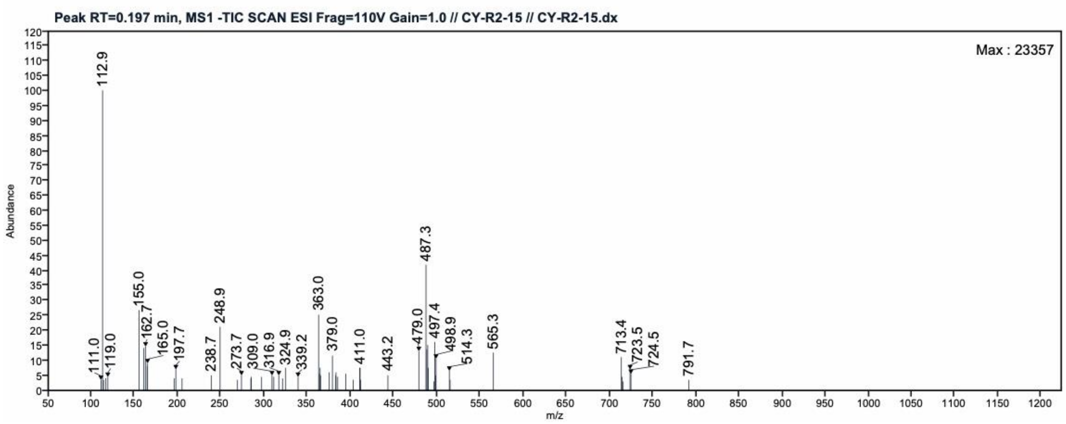

HPLC chromatogram of *1-((1-(2-Chloro-6-methylphenyl)-1H-1,2,3-triazol-4-yl)methyl)-3-methyl-1H-pyrazolo[3,4-b]pyridine* (15)

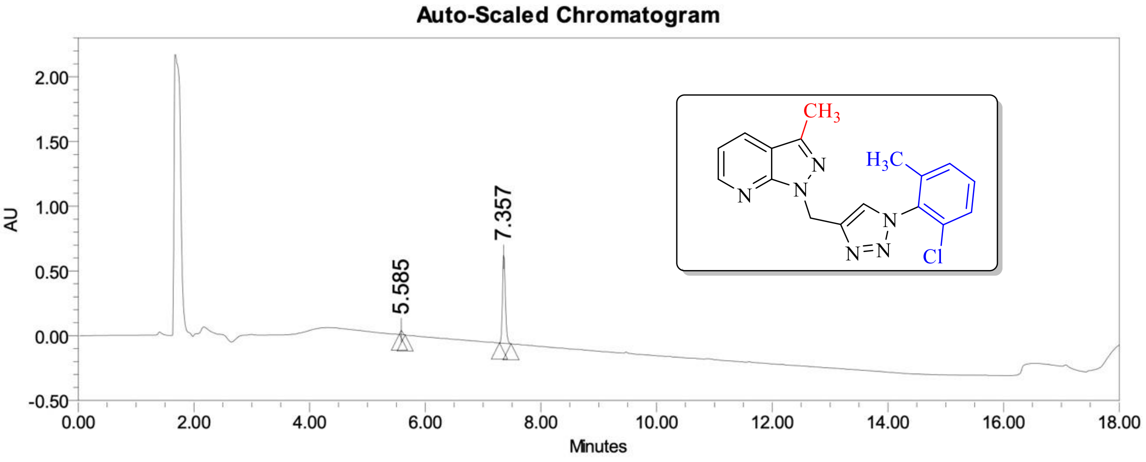

Peak Results

	Name	RT	Area	% Area
1		5.585	82860	3.37
2		7.357	2373545	96.63

^{1}H NMR spectrum of *1-((1-(4-Chloro-2-iodophenyl)-1H-1,2,3-triazol-4-yl)methyl)-3-methyl-1H-pyrazolo[3,4-b]pyridine* **(16)**

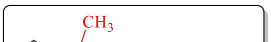

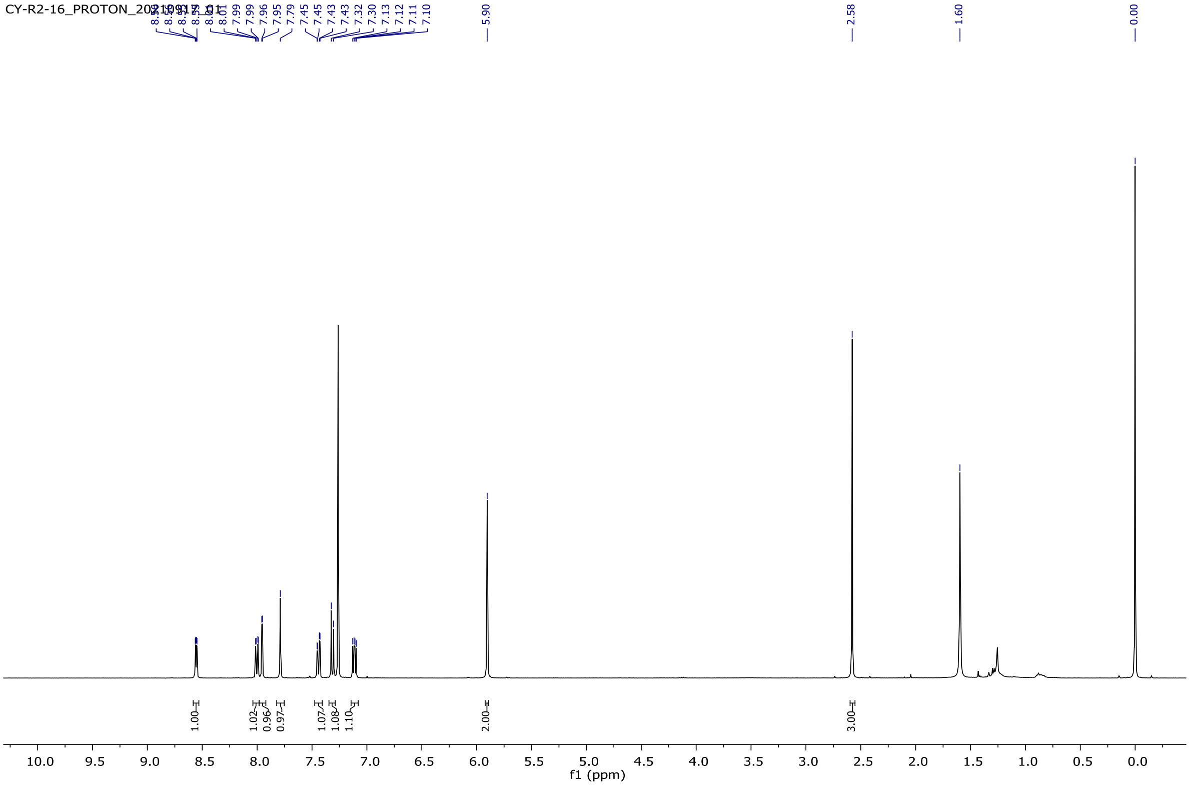

^{13}C NMR spectrum of *1-((1-(4-Chloro-2-iodophenyl)-1H-1,2,3-triazol-4-yl)methyl)-3-methyl-1H-pyrazolo[3,4-b]pyridine* **(16)**

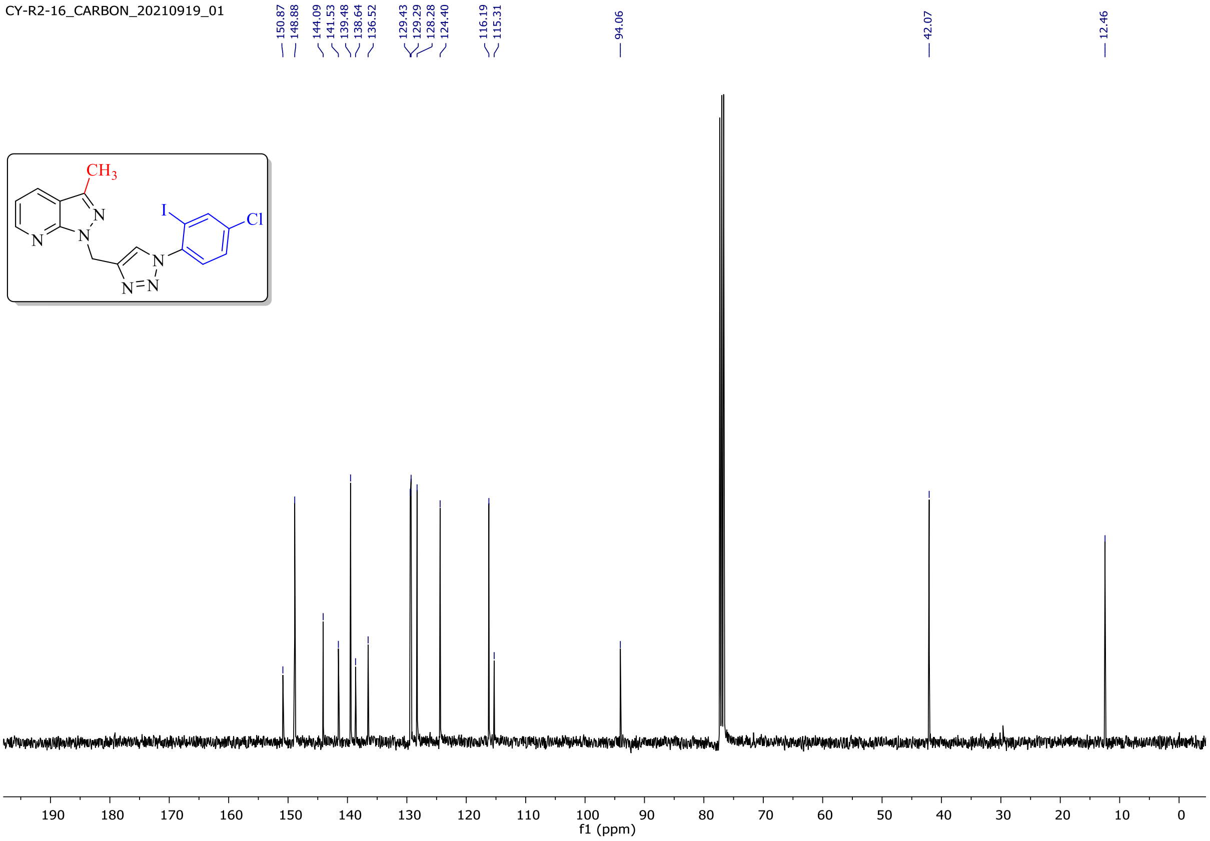

IR spectrum of *1-((1-(4-Chloro-2-iodophenyl)-1H-1,2,3-triazol-4-yl)methyl)-3-methyl-1H-pyrazolo[3,4-b]pyridine* **(16)**

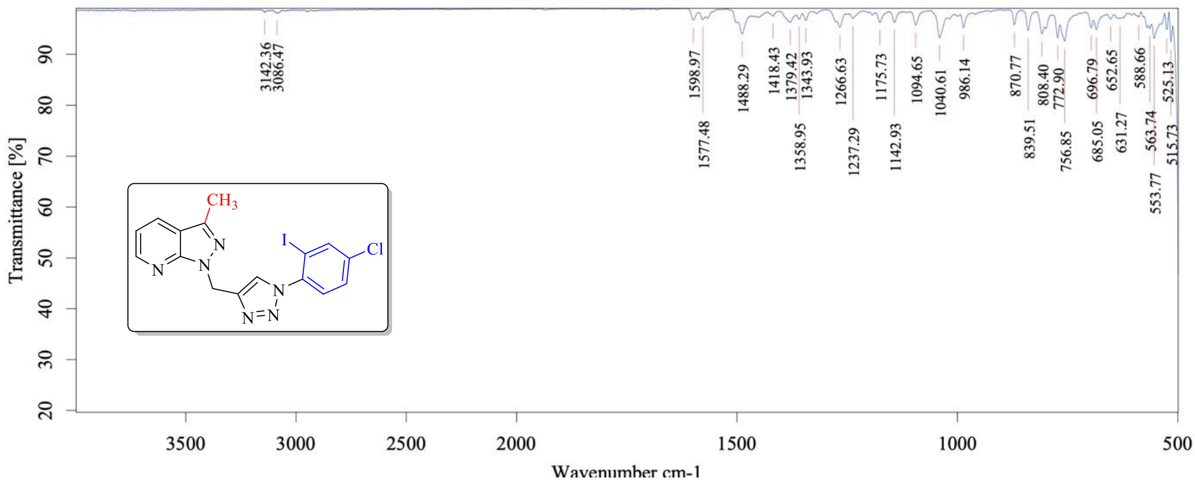

Mass spectrum of *1-((1-(4-Chloro-2-iodophenyl)-1H-1,2,3-triazol-4-yl)methyl)-3-methyl-1H-pyrazolo[3,4-b]pyridine* **(16)**

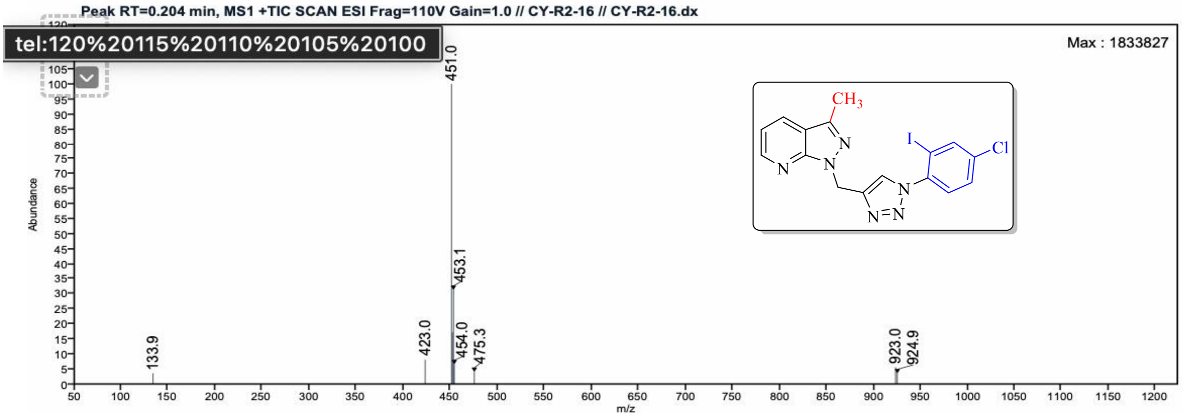

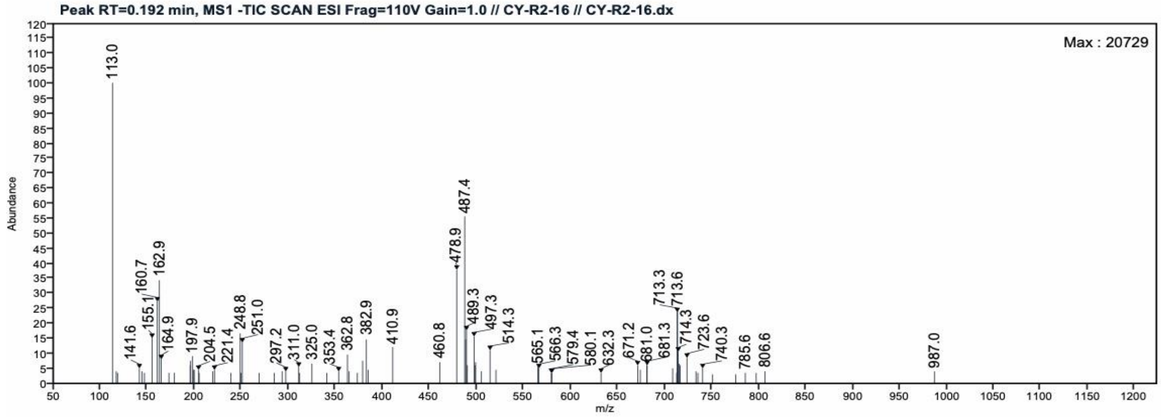

HPLC chromatogram of *1-((1-(4-Chloro-2-iodophenyl)-1H-1,2,3-triazol-4-yl)methyl)-3-methyl-1H-pyrazolo[3,4-b]pyridine* **(16)**

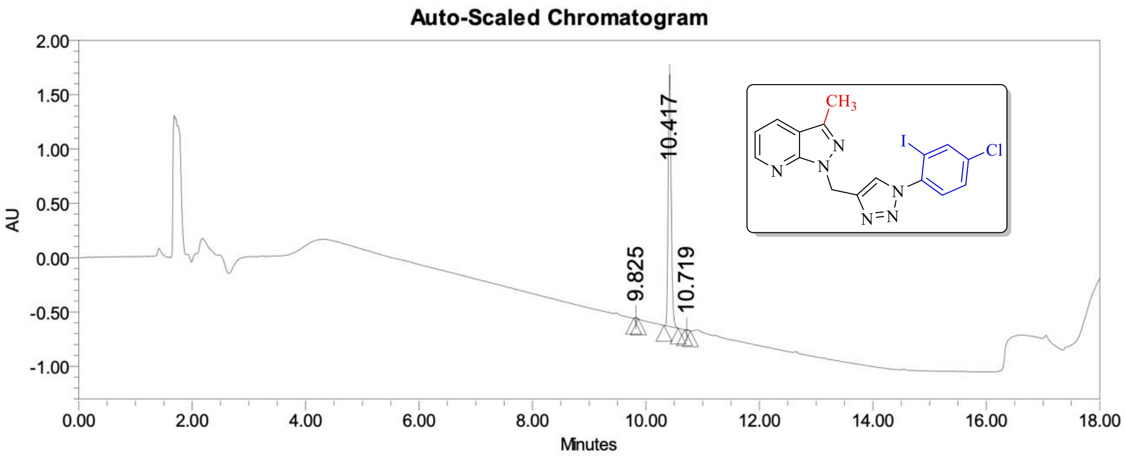

Peak Results

	Name	RT	Area	% Area
1		9.825	39331	0.44
2		10.417	8898778	99.35
3		10.719	19338	0.22

^{1}H NMR spectrum of *1-((1-(4-Fluorophenyl)-1H-1,2,3-triazol-4-yl)methyl)-3-methyl-1H-pyrazolo[3,4-b]pyridine* (17)

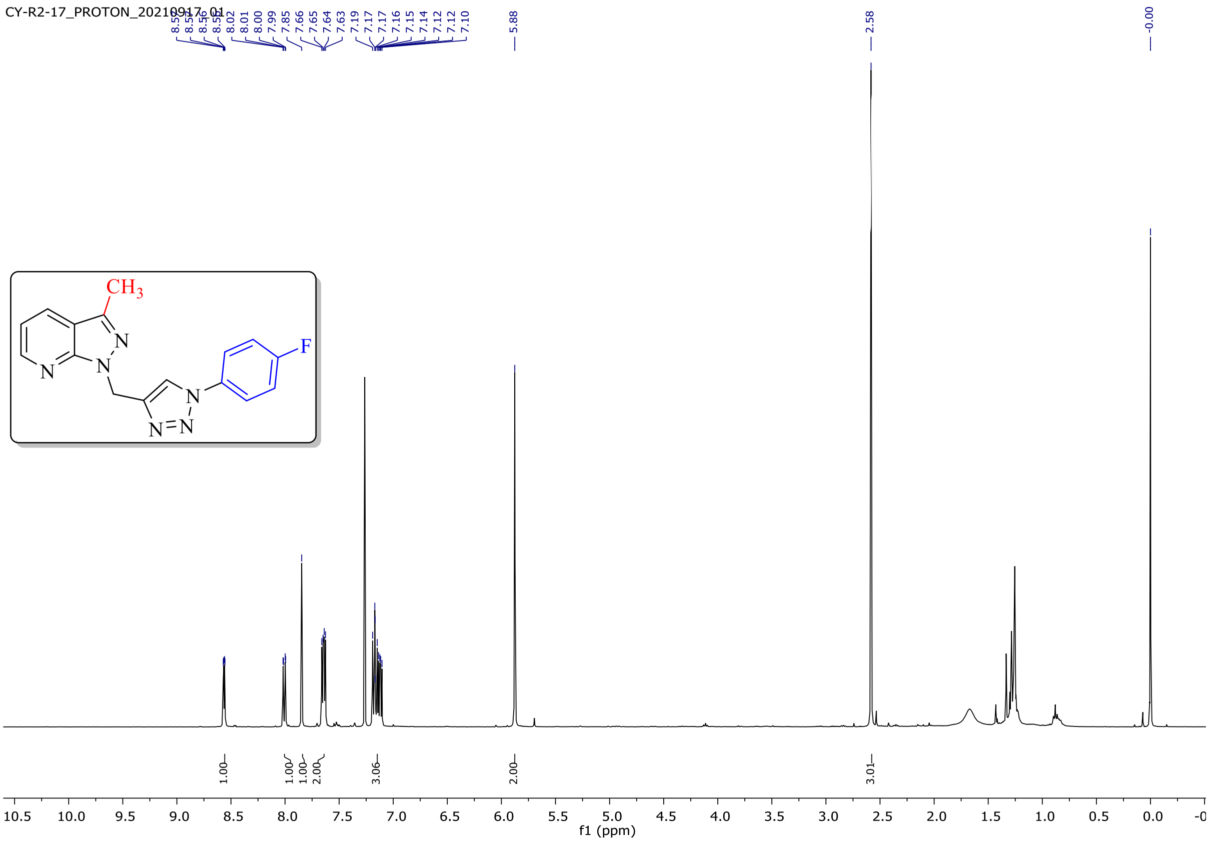

^{13}C NMR spectrum of *1-((1-(4-Fluorophenyl)-1H-1,2,3-triazol-4-yl)methyl)-3-methyl-1H-pyrazolo[3,4-b]pyridine* (17)

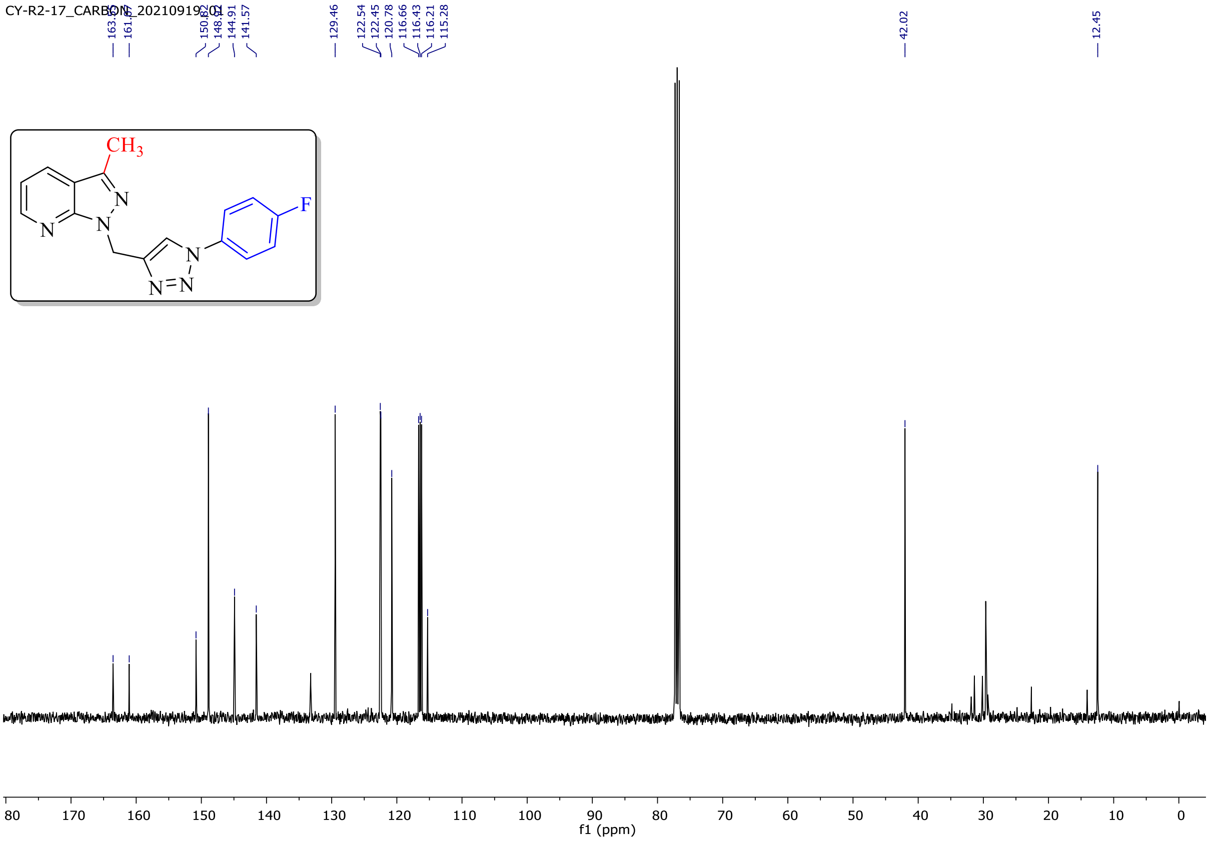

IR spectrum of *1-((1-(4-Fluorophenyl)-1H-1,2,3-triazol-4-yl)methyl)-3-methyl-1H-pyrazolo[3,4-b]pyridine* (17)

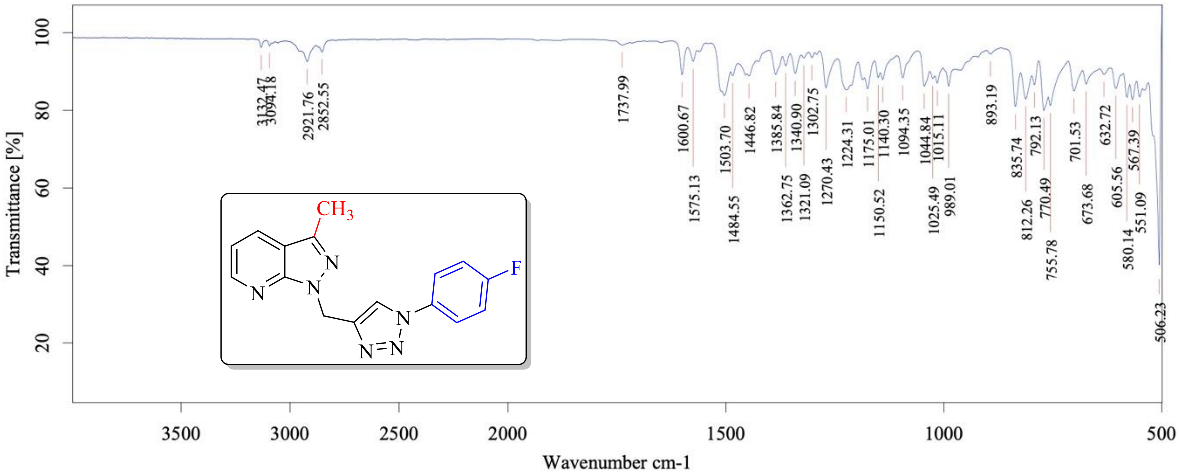

Mass spectrum of *1-((1-(4-Fluorophenyl)-1H-1,2,3-triazol-4-yl)methyl)-3-methyl-1H-pyrazolo[3,4-b]pyridine* (17)

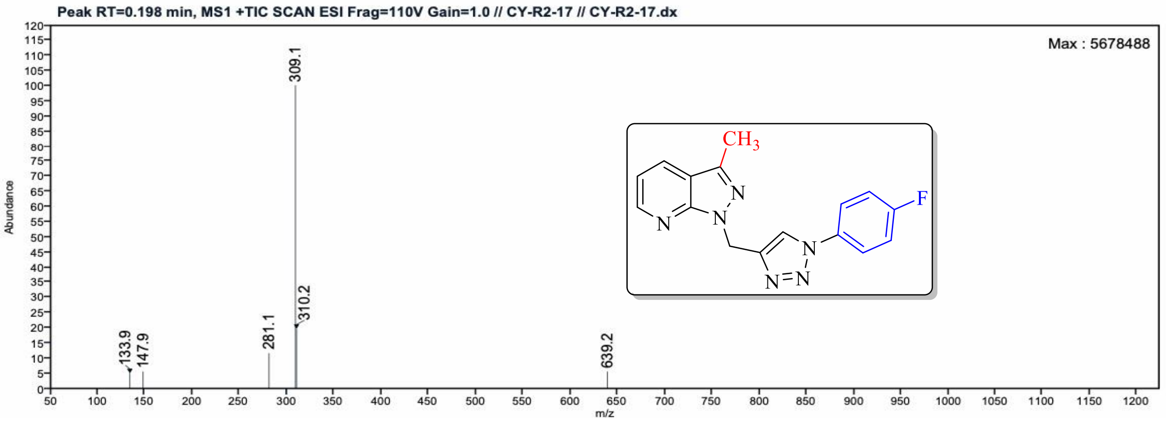

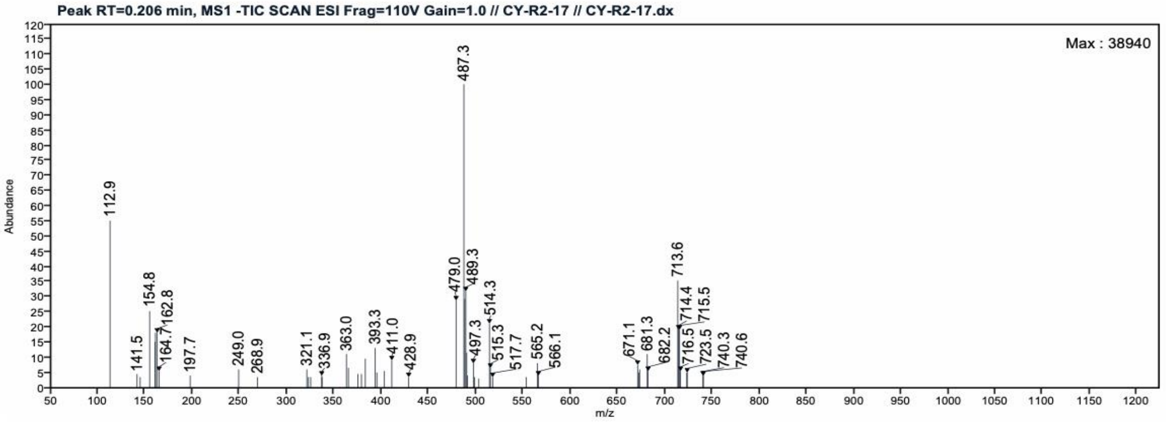

HPLC chromatogram of *1-((1-(4-Fluorophenyl)-1H-1,2,3-triazol-4-yl)methyl)-3-methyl-1H-pyrazolo[3,4-b]pyridine* (17)

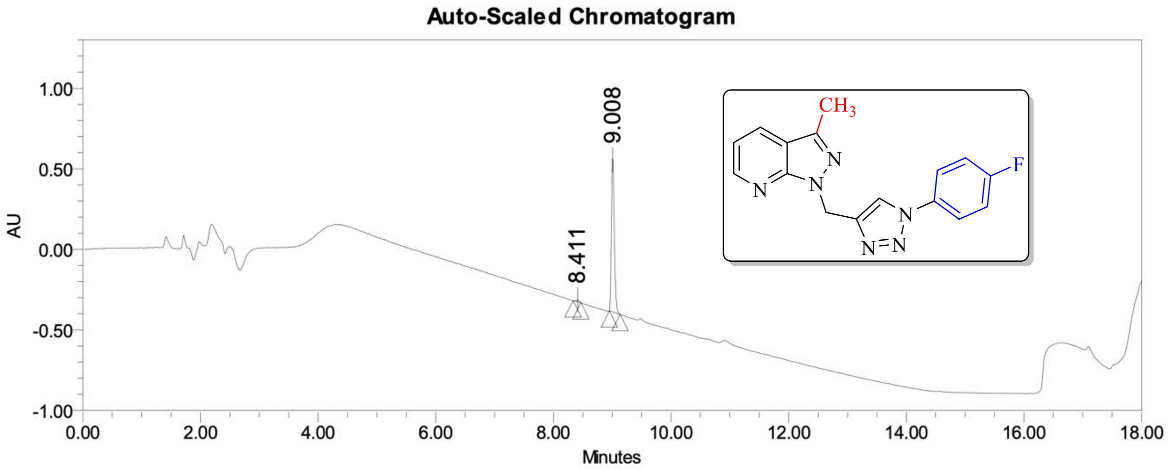

Peak Results

	Name	RT	Area	% Area
1		8.411	40037	1.20
2		9.008	3290473	98.80

^{1}H NMR spectrum of *1-((1-(3-Chlorophenyl)-1H-1,2,3-triazol-4-yl)methyl)-3-methyl-1H-pyrazolo[3,4-b]pyridine* (18)

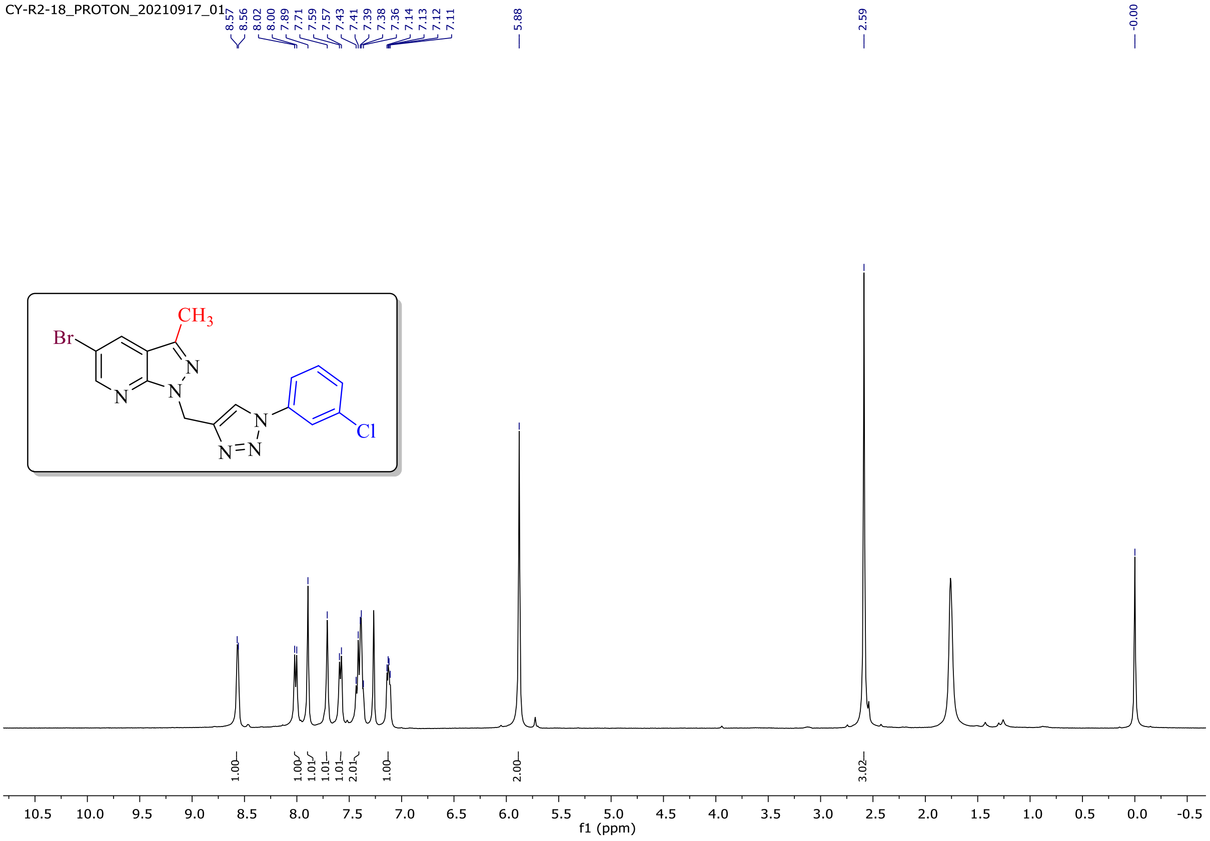

^{13}C NMR spectrum of *1-((1-(3-Chlorophenyl)-1H-1,2,3-triazol-4-yl)methyl)-3-methyl-1H-pyrazolo[3,4-b]pyridine* (18)

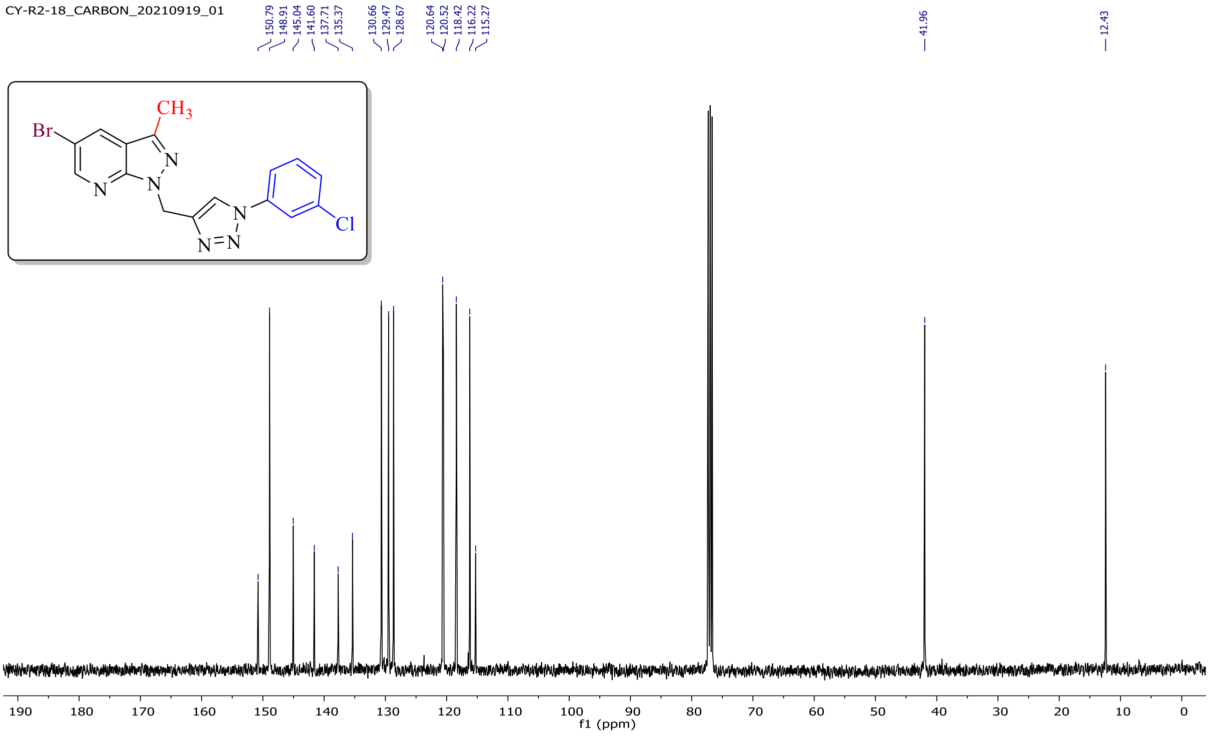

Mass spectrum of *1-((1-(3-Chlorophenyl)-1H-1,2,3-triazol-4-yl)methyl)-3-methyl-1H-pyrazolo[3,4-b]pyridine* (18)

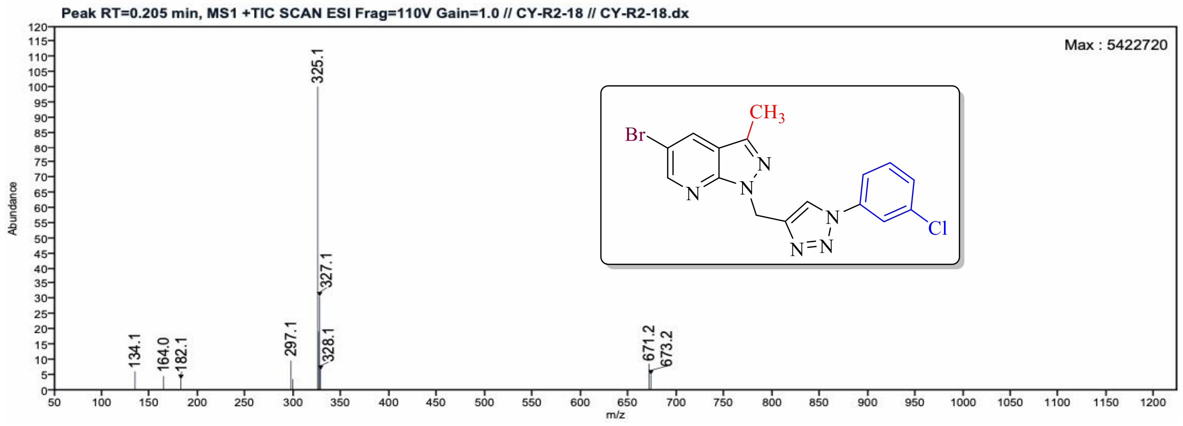

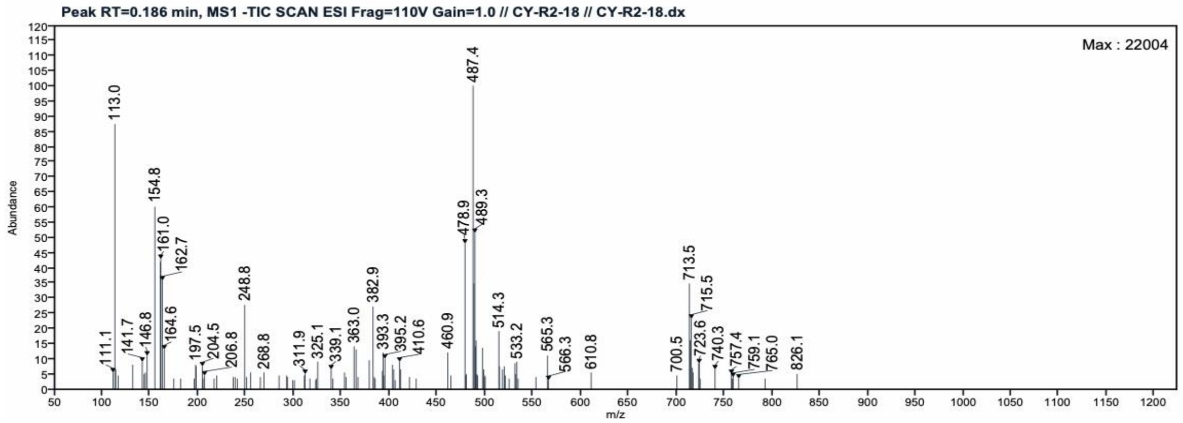

HPLC chromatogram of *1-((1-(3-Chlorophenyl)-1H-1,2,3-triazol-4-yl)methyl)-3-methyl-1H-pyrazolo[3,4-b]pyridine* (18)

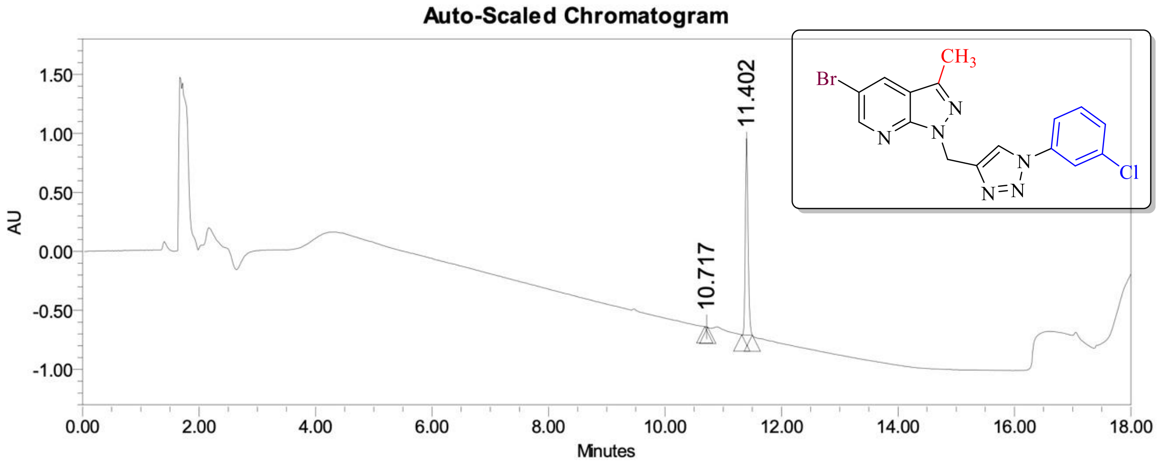

Peak Results

	Name	RT	Area	% Area
1		10.717	4609	0.08
2		11.402	5474714	99.92

^{1}H NMR spectrum of *5-Bromo-1-((1-(2-chloro-6-methylphenyl)-1H-1,2,3-triazol-4-yl)methyl)-3-methyl-1H-pyrazolo[3,4-b]pyridine* (19)

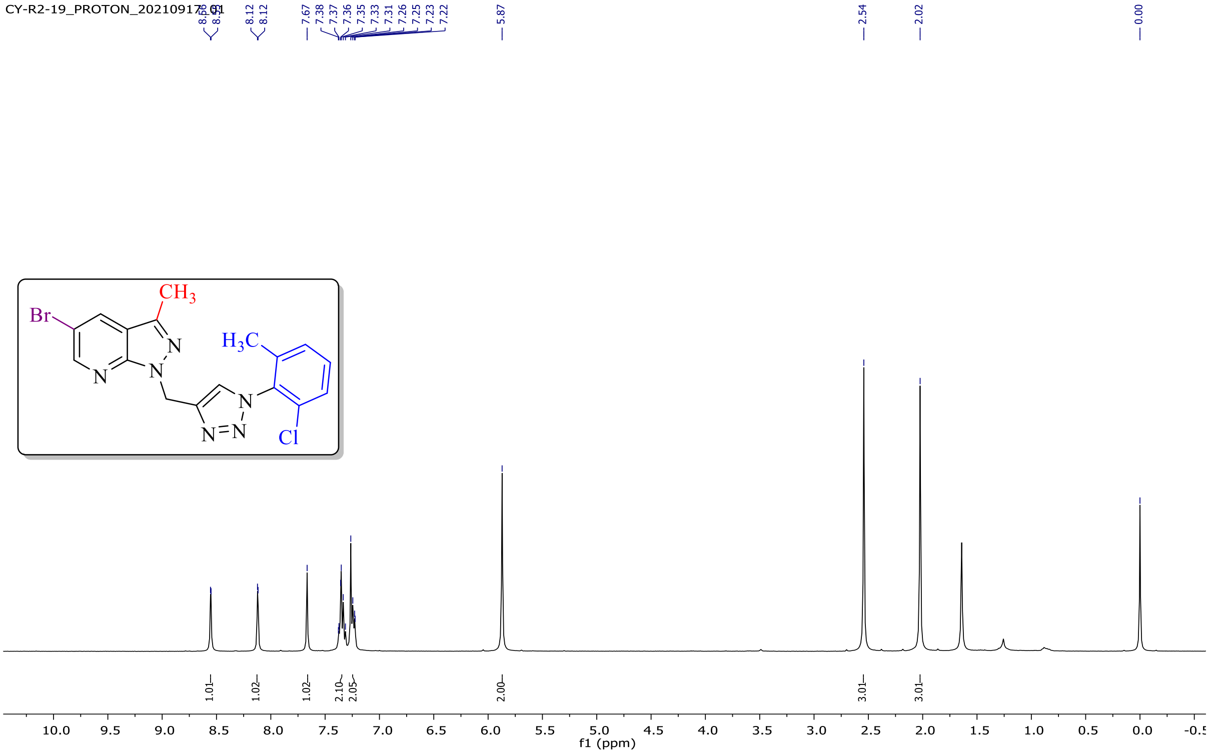

^{13}C NMR spectrum of *5-Bromo-1-((1-(2-chloro-6-methylphenyl)-1H-1,2,3-triazol-4-yl)methyl)-3-methyl-1H-pyrazolo[3,4-b]pyridine* (19)

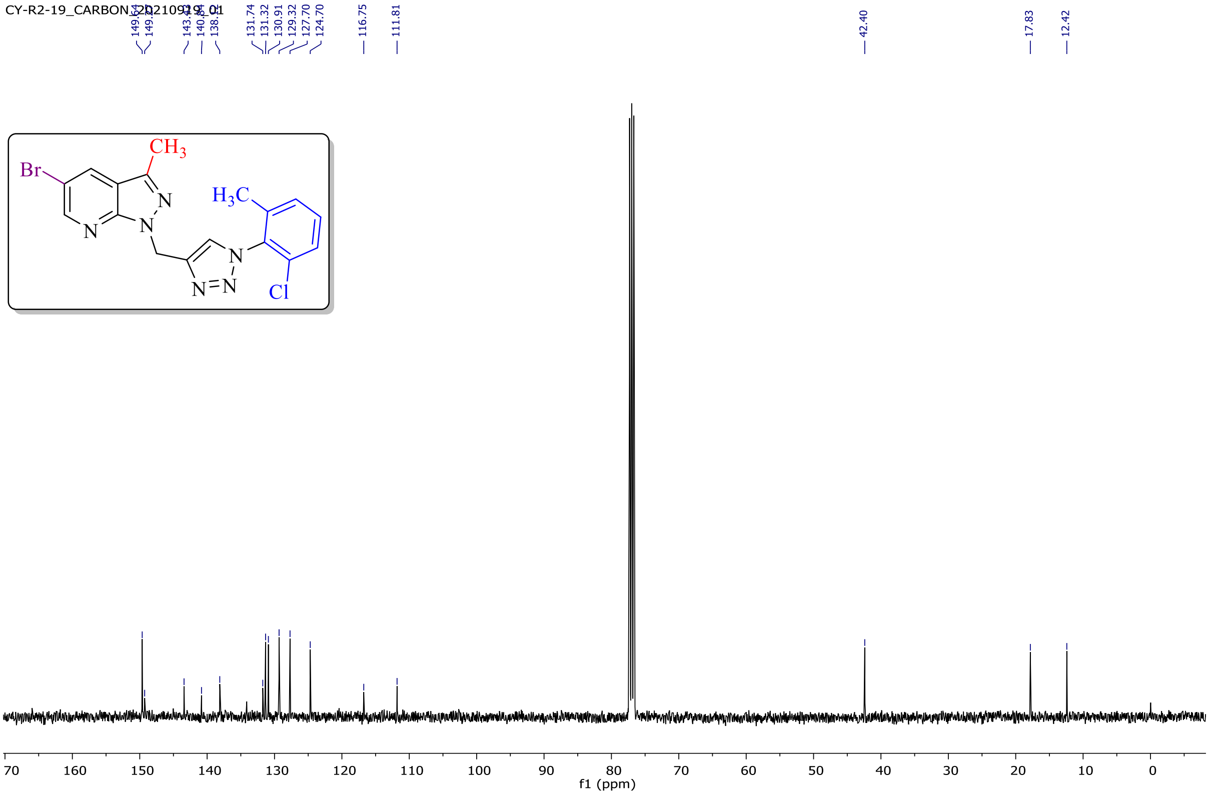

IR spectrum of *5-Bromo-1-((1-(2-chloro-6-methylphenyl)-1H-1,2,3-triazol-4-yl)methyl)-3-methyl-1H-pyrazolo[3,4-b]pyridine* (19)

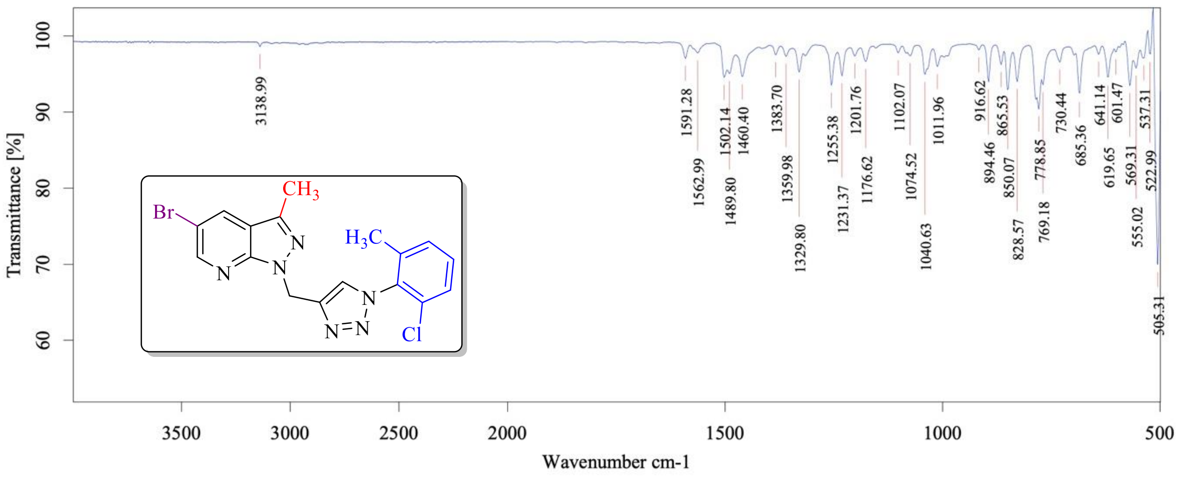

Mass spectrum of *5-Bromo-1-((1-(2-chloro-6-methylphenyl)-1H-1,2,3-triazol-4-yl)methyl)-3-methyl-1H-pyrazolo[3,4-b]pyridine* (19)

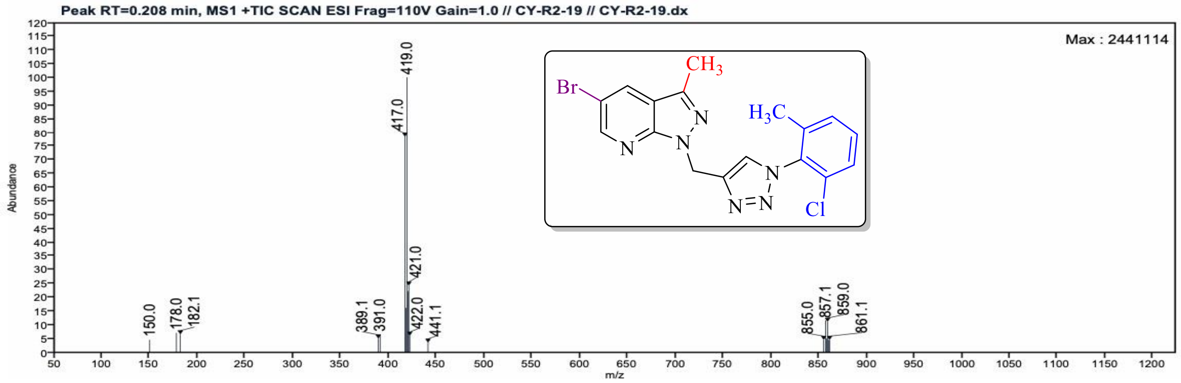

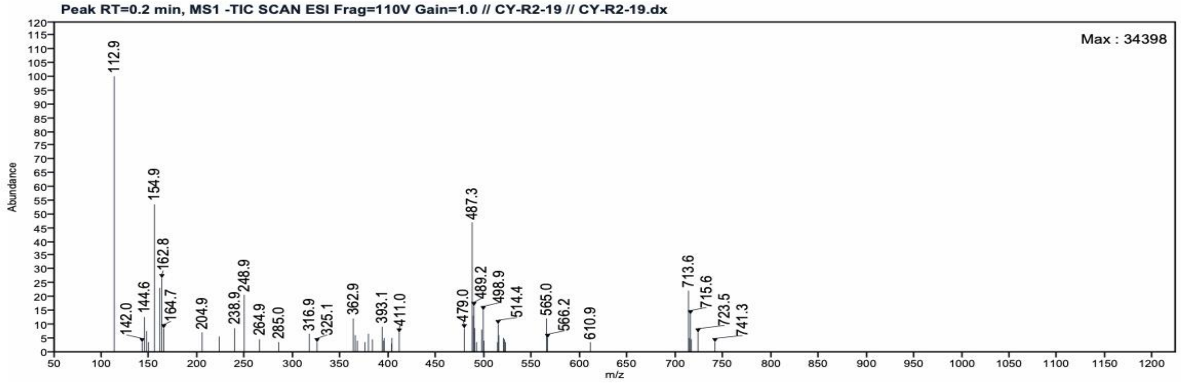

HPLC chromatogram of *5-Bromo-1-((1-(2-chloro-6-methylphenyl)-1H-1,2,3-triazol-4-yl)methyl)-3-methyl-1H-pyrazolo[3,4-b]pyridine* (19)

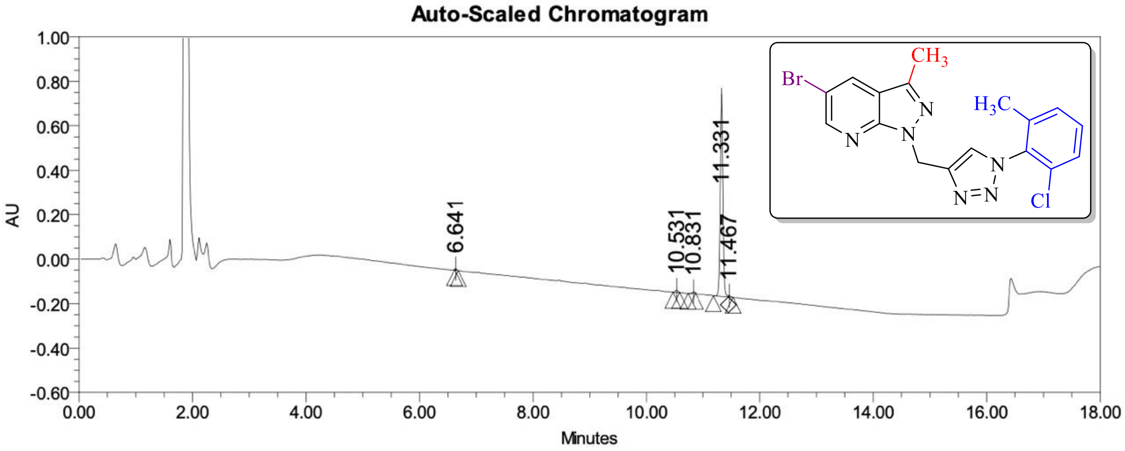

Peak Results

	Name	RT	Area	% Area
1		6.641	14263	0.48
2		10.531	34427	1.16
3		10.831	22944	0.78
4		11.331	2869270	97.05
5		11.467	15584	0.53

^{1}H NMR spectrum of *5-Bromo-1-((1-(4-fluorophenyl)-1H-1,2,3-triazol-4-yl)methyl)-3-methyl-1H-pyrazolo[3,4-b]pyridine* (20)

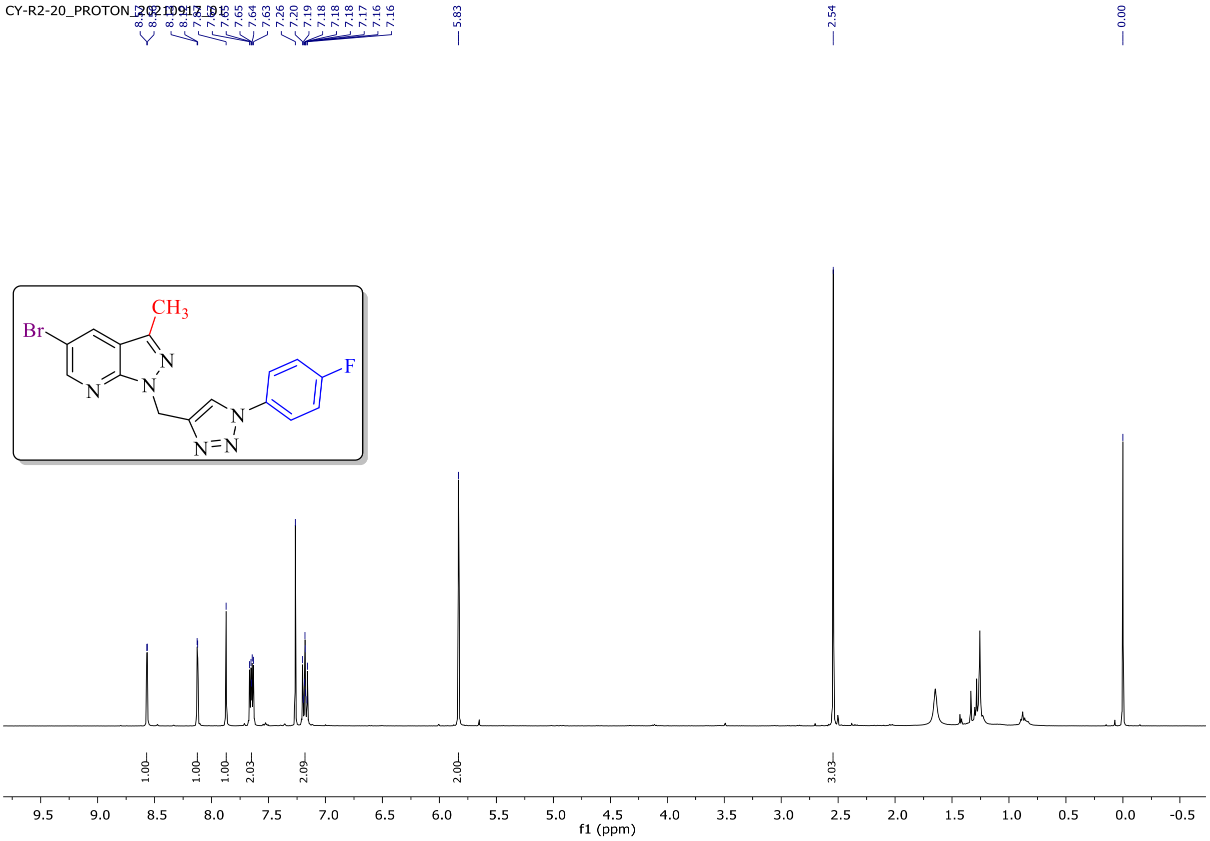

^{13}C NMR spectrum of *5-Bromo-1-((1-(4-fluorophenyl)-1H-1,2,3-triazol-4-yl)methyl)-3-methyl-1H-pyrazolo[3,4-b]pyridine* (20)

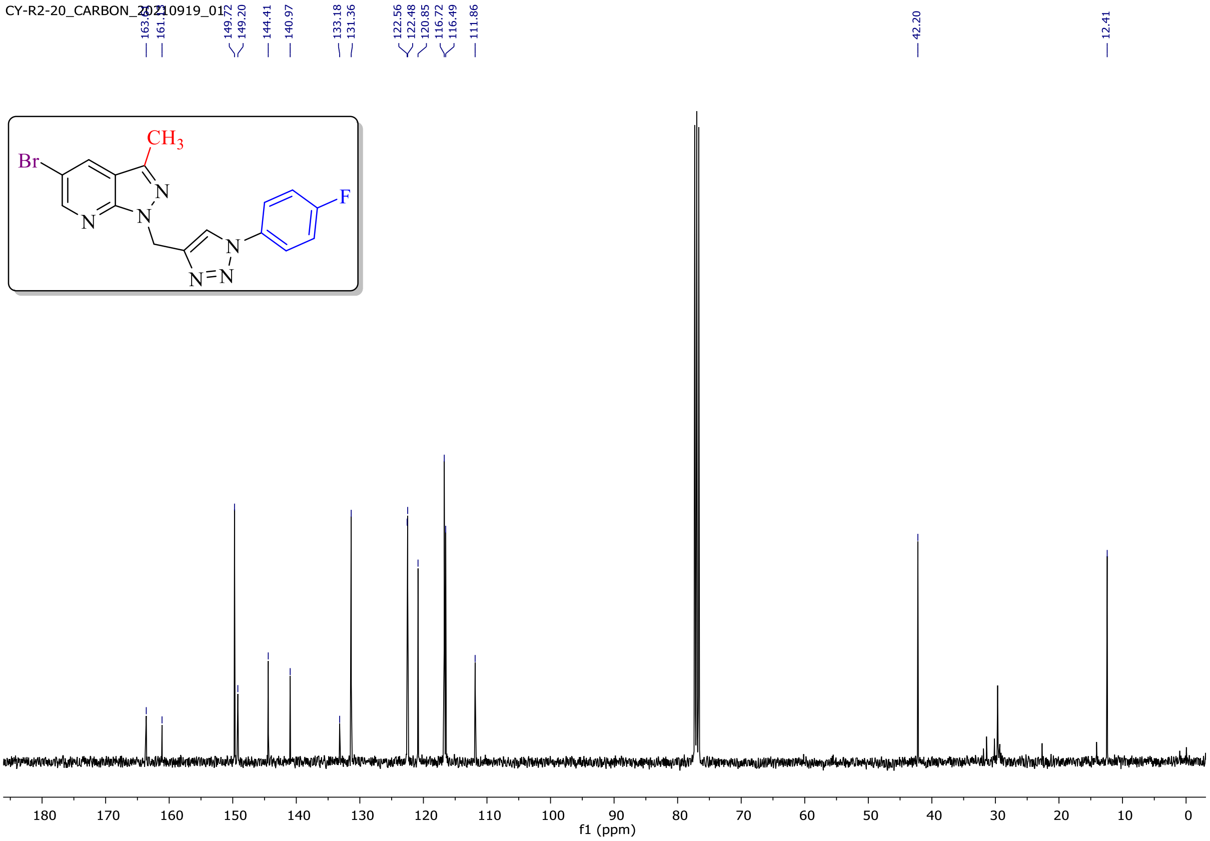

IR spectrum of *5-Bromo-1-((1-(4-fluorophenyl)-1H-1,2,3-triazol-4-yl)methyl)-3-methyl-1H-pyrazolo[3,4-b]pyridine* (20)

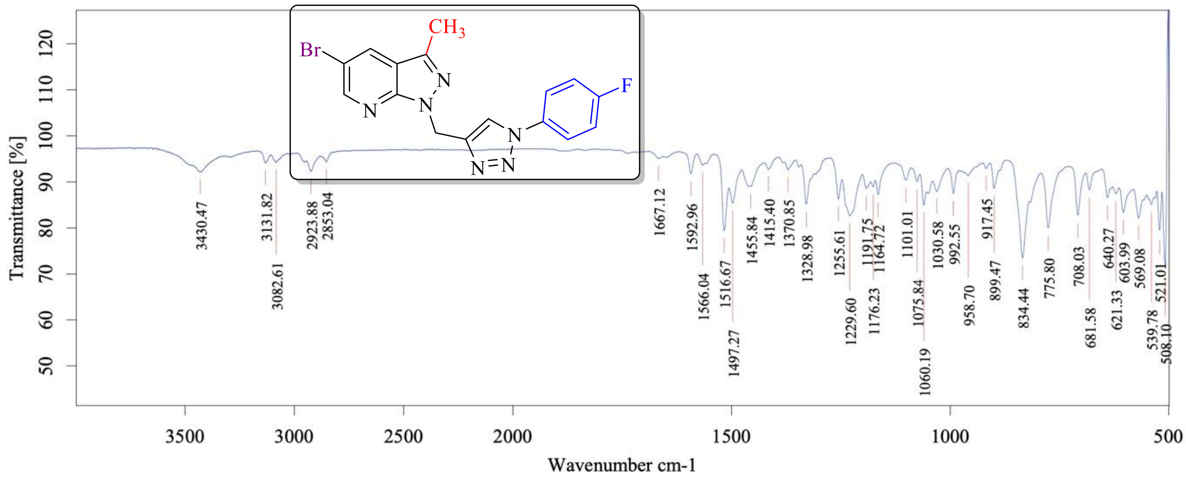

Mass spectrum of *5-Bromo-1-((1-(4-fluorophenyl)-1H-1,2,3-triazol-4-yl)methyl)-3-methyl-1H-pyrazolo[3,4-b]pyridine* (20)

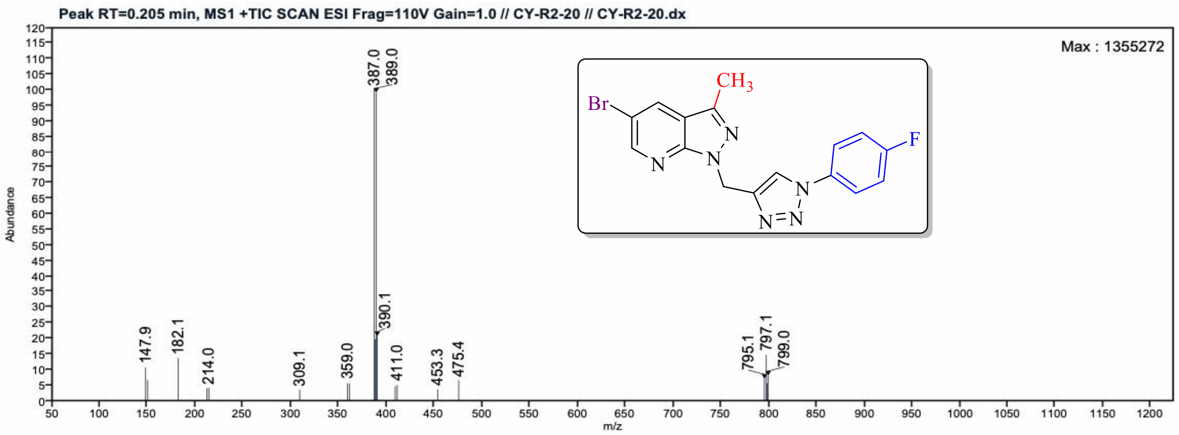

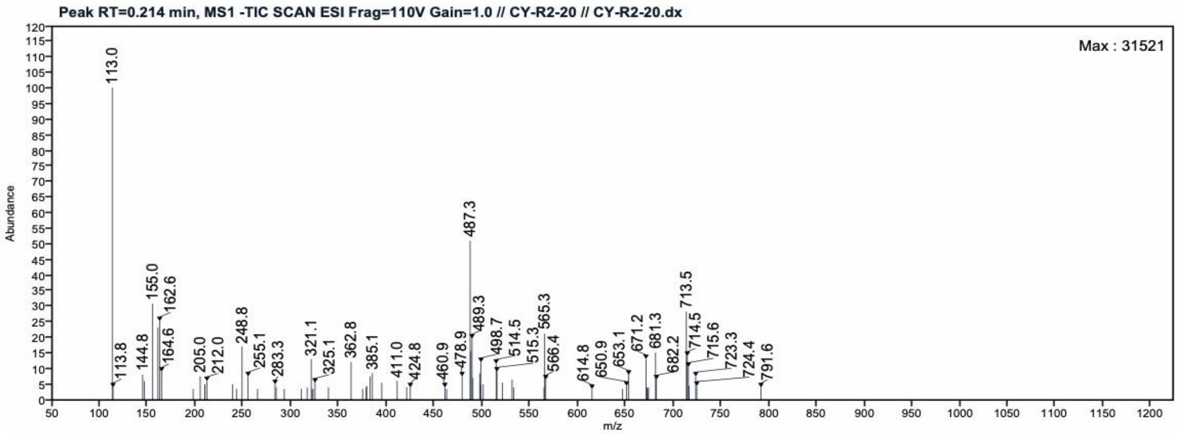

HPLC chromatogram of *5-Bromo-1-((1-(4-fluorophenyl)-1H-1,2,3-triazol-4-yl)methyl)-3-methyl-1H-pyrazolo[3,4-b]pyridine* (20)

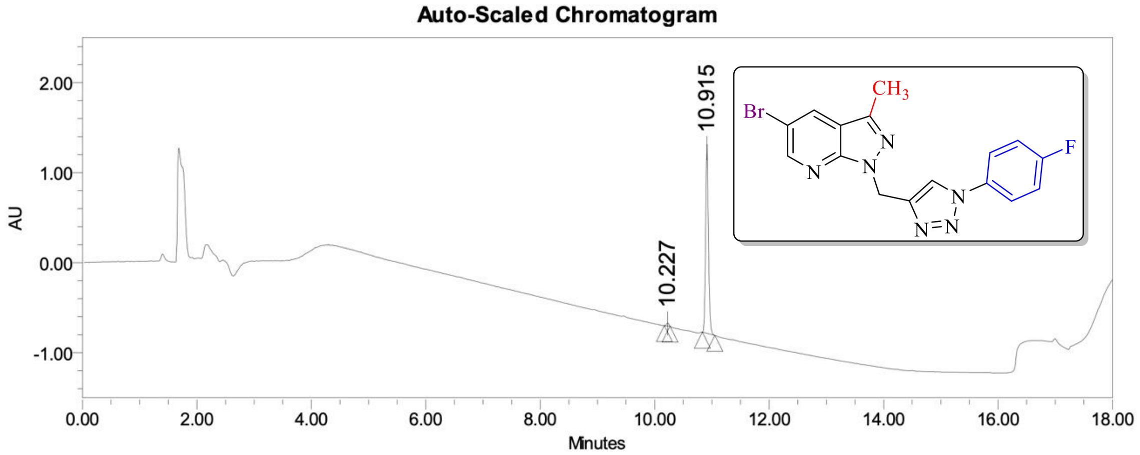

Peak Results

	Name	RT	Area	% Area
1		10.227	90125	1.28
2		10.915	6944173	98.72

^{1}H NMR spectrum of *5-Bromo-1-((1-(4-chlorophenyl)-1H-1,2,3-triazol-4-yl)methyl)-3-methyl-1H-pyrazolo[3,4-b]pyridine* (21)

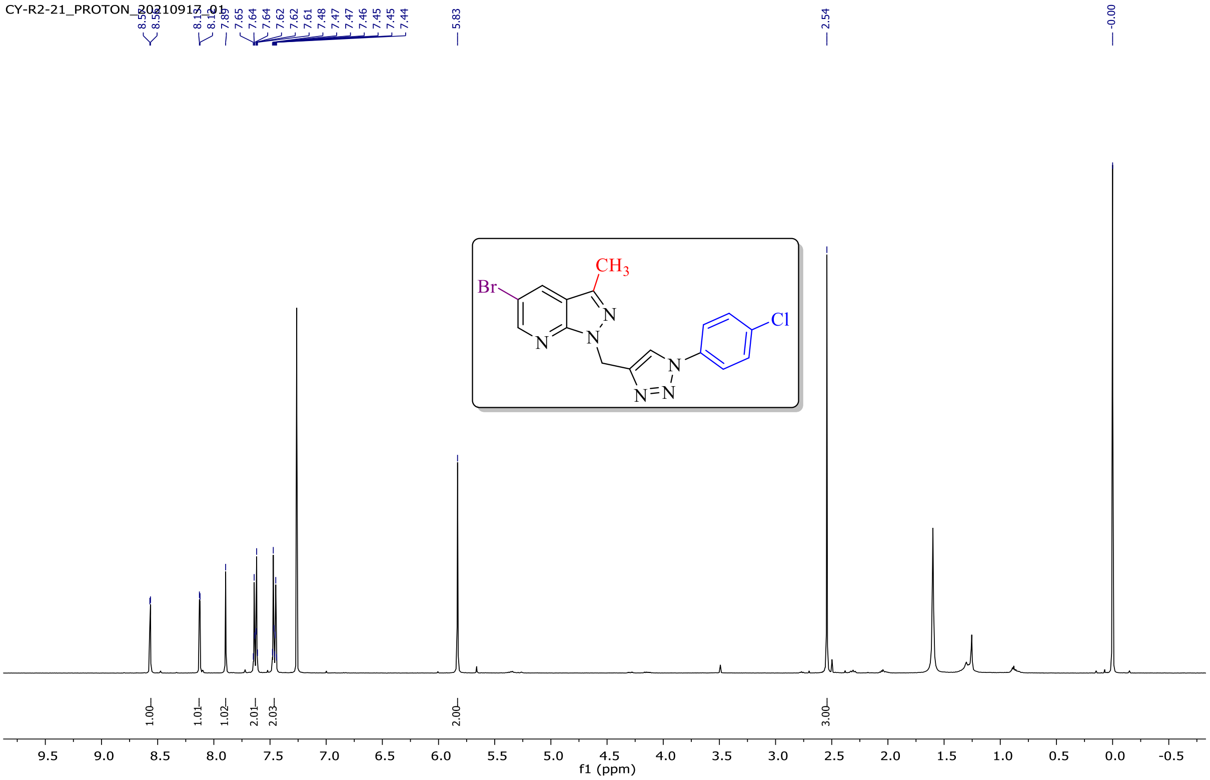

^{13}C NMR spectrum of *5-Bromo-1-((1-(4-chlorophenyl)-1H-1,2,3-triazol-4-yl)methyl)-3-methyl-1H-pyrazolo[3,4-b]pyridine* (21)

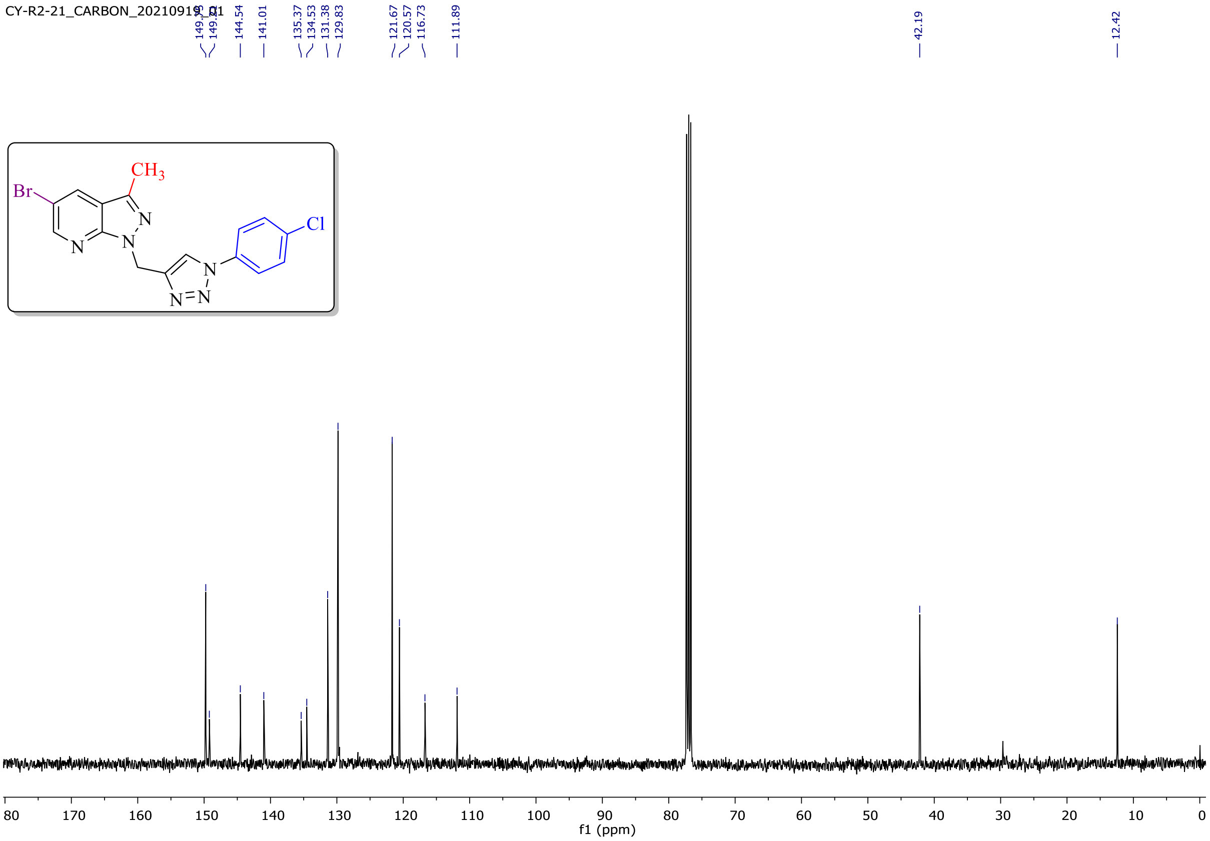

IR spectrum of *5-Bromo-1-((1-(4-chlorophenyl)-1H-1,2,3-triazol-4-yl)methyl)-3-methyl-1H-pyrazolo[3,4-b]pyridine* (21)

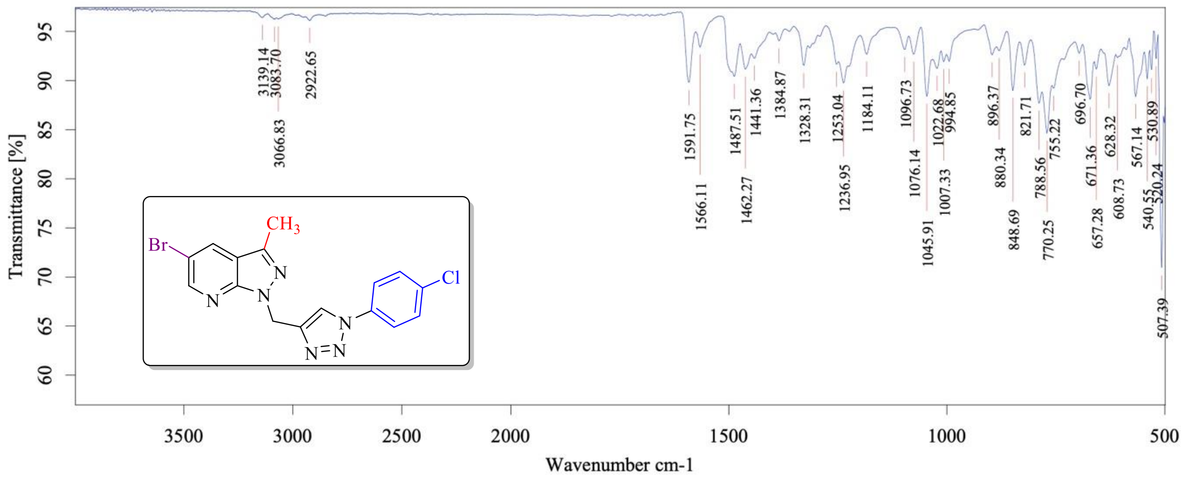

Mass spectrum of *5-Bromo-1-((1-(4-chlorophenyl)-1H-1,2,3-triazol-4-yl)methyl)-3-methyl-1H-pyrazolo[3,4-b]pyridine* (21)

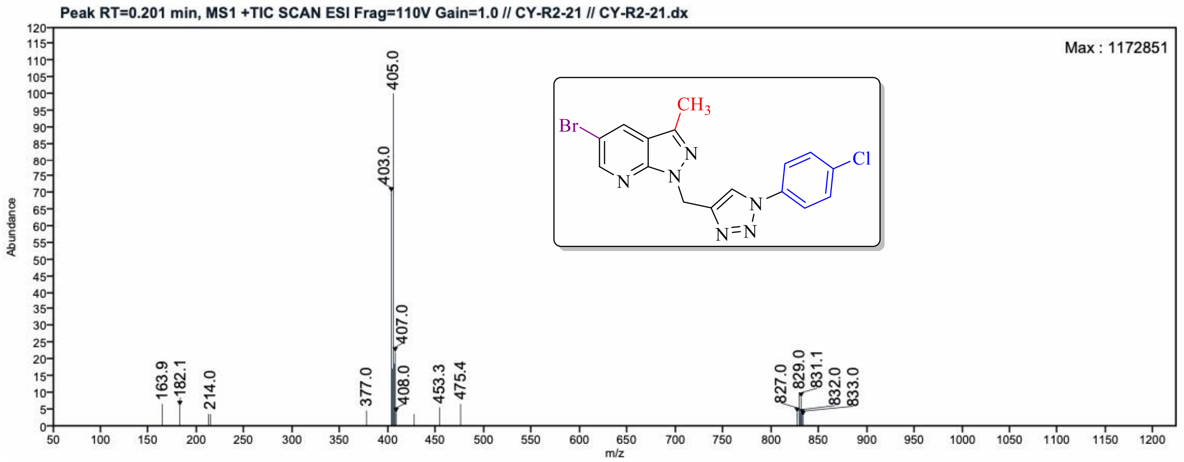

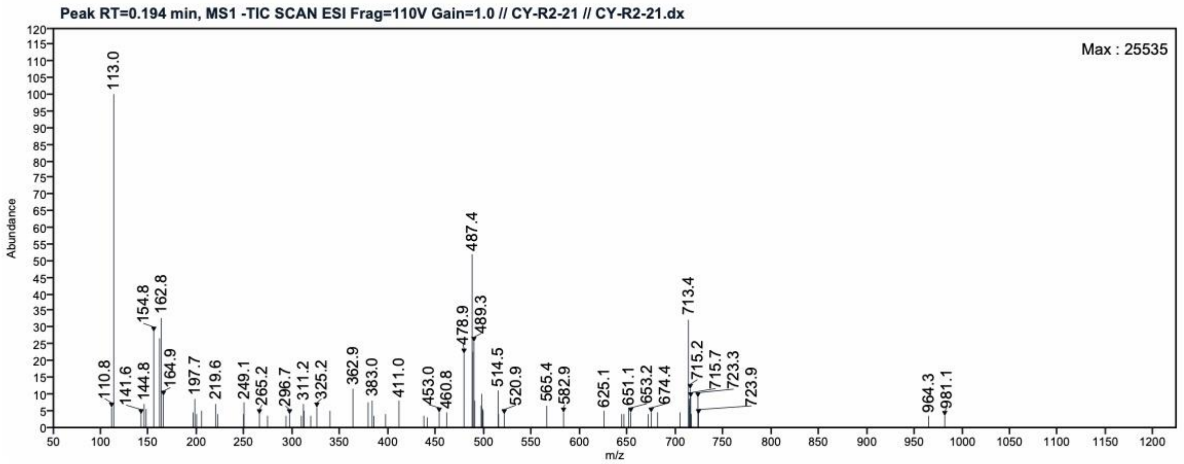

HPLC chromatogram of *5-Bromo-1-((1-(4-chlorophenyl)-1H-1,2,3-triazol-4-yl)methyl)-3-methyl-1H-pyrazolo[3,4-b]pyridine* (21)

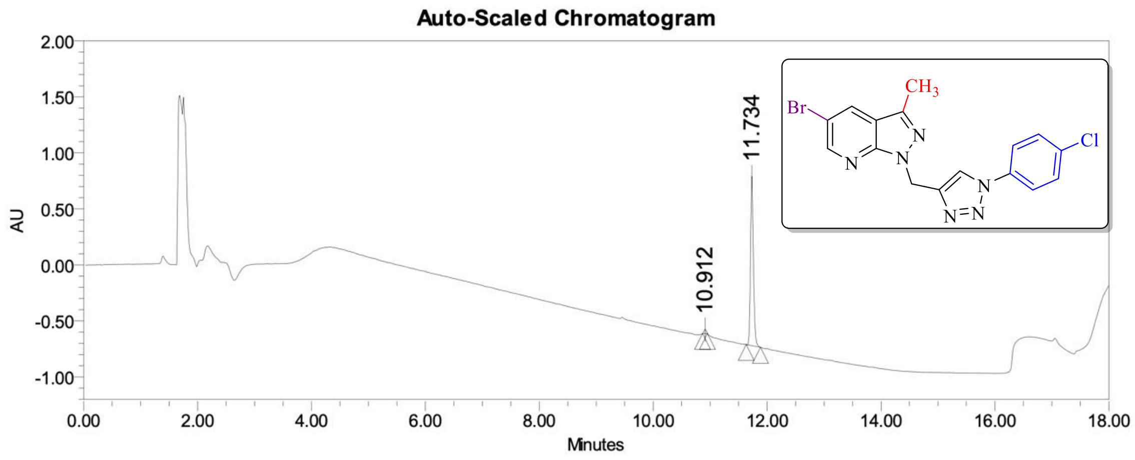

Peak Results

	Name	RT	Area	% Area
1		10.912	101965	1.97
2		11.734	5085406	98.03

^{1}H NMR spectrum of *5-Bromo-1-((1-(3-chlorophenyl)-1H-1,2,3-triazol-4-yl)methyl)-3-methyl-1H-pyrazolo[3,4-b]pyridine* (22)

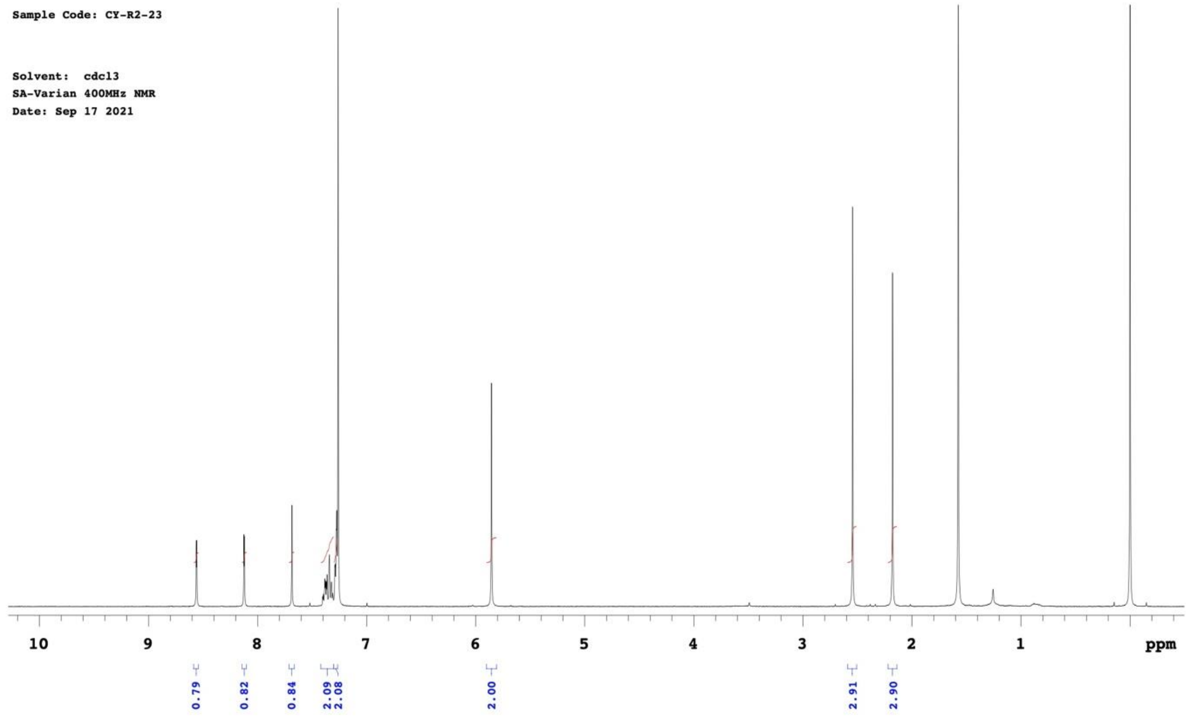

^{13}C NMR spectrum of *5-Bromo-1-((1-(3-chlorophenyl)-1H-1,2,3-triazol-4-yl)methyl)-3-methyl-1H-pyrazolo[3,4-b]pyridine* (22)

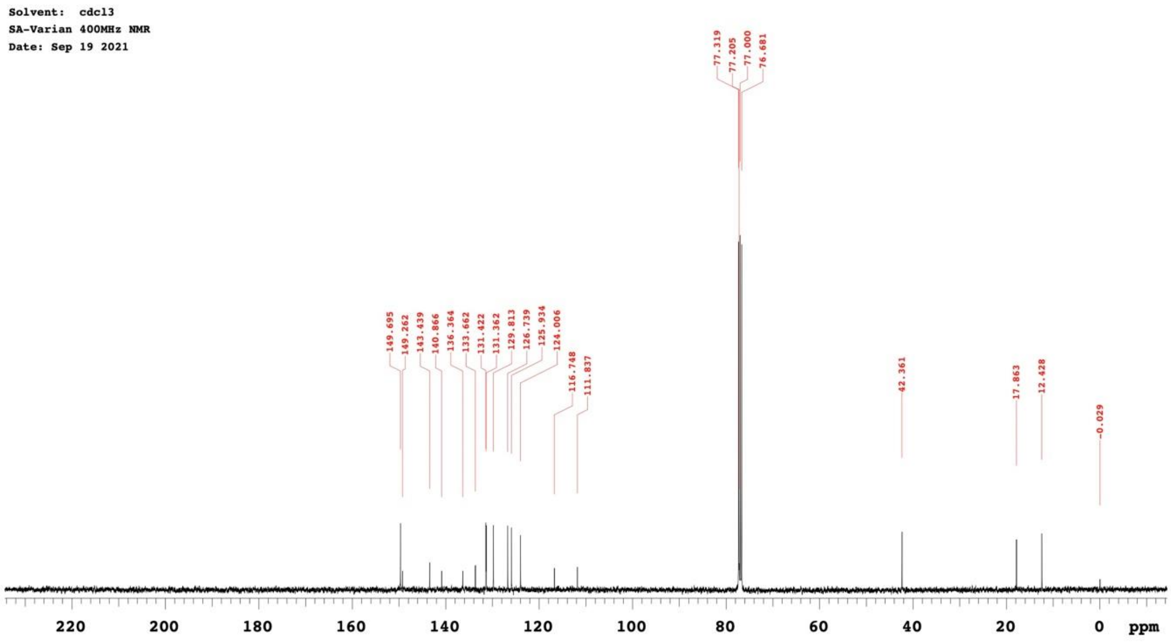

IR spectrum of *5-Bromo-1-((1-(3-chlorophenyl)-1H-1,2,3-triazol-4-yl)methyl)-3-methyl-1H-pyrazolo[3,4-b]pyridine* (22)

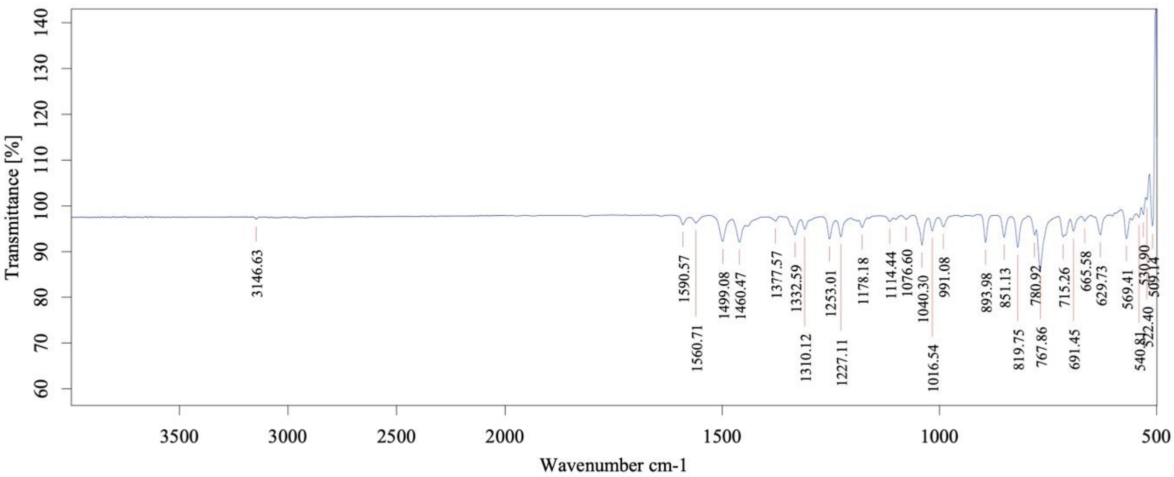

Mass spectrum of *5-Bromo-1-((1-(3-chlorophenyl)-1H-1,2,3-triazol-4-yl)methyl)-3-methyl-1H-pyrazolo[3,4-b]pyridine* (22)

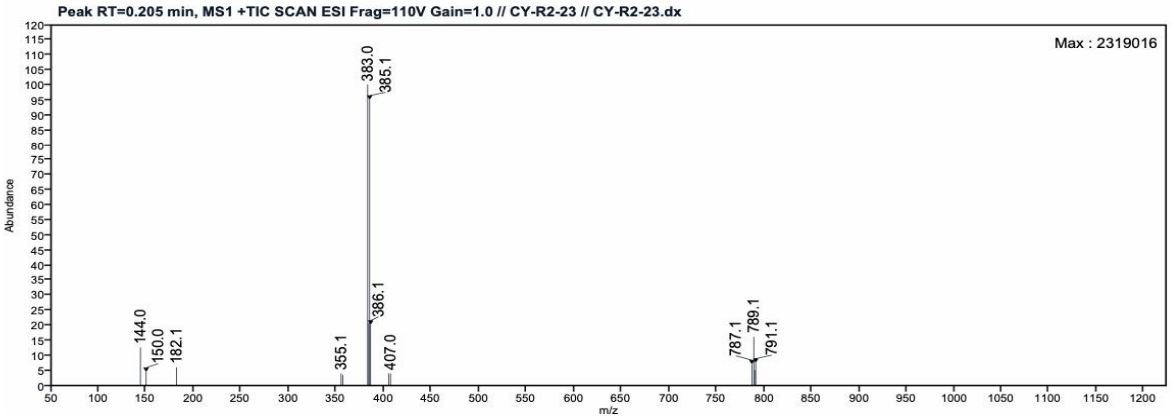

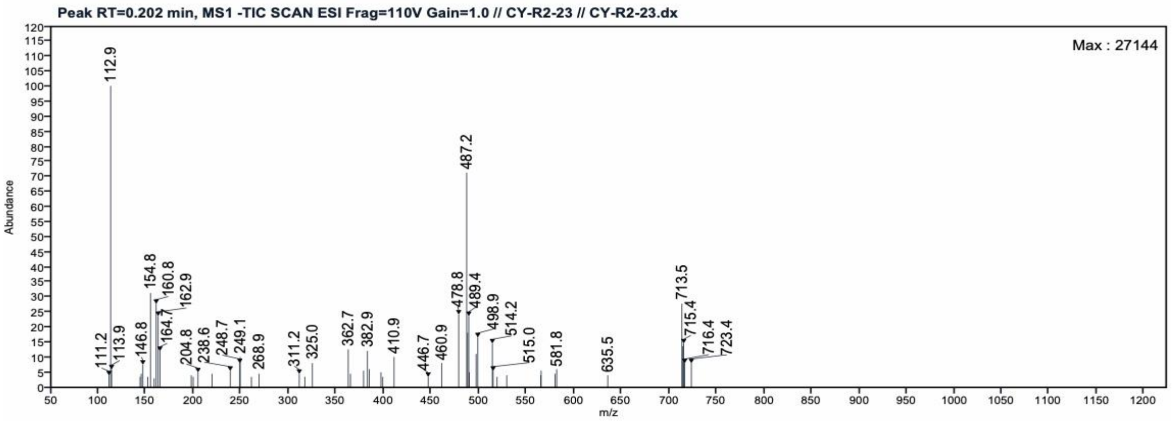

HPLC chromatogram of *5-Bromo-1-((1-(3-chlorophenyl)-1H-1,2,3-triazol-4-yl)methyl)-3-methyl-1H-pyrazolo[3,4-b]pyridine* (22)

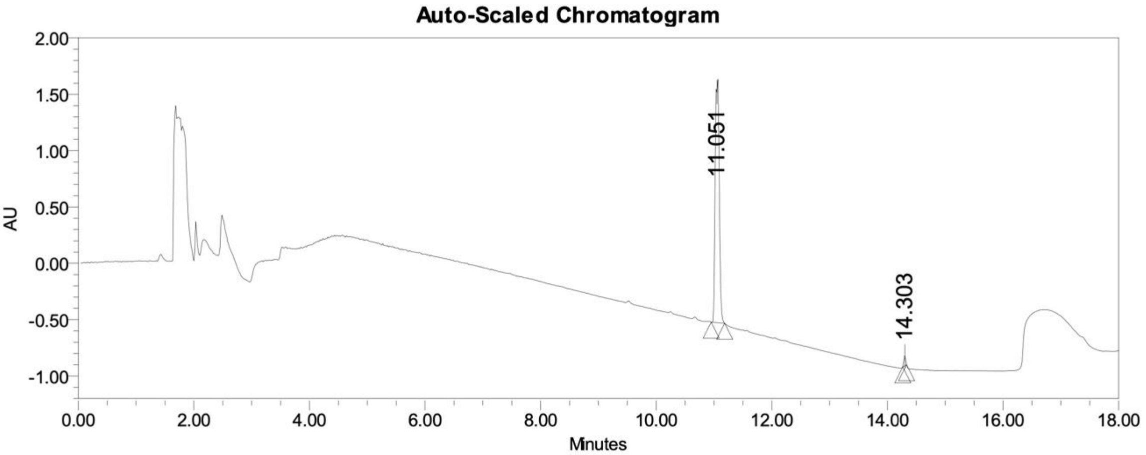

Peak Results

	Name	RT	Area	% Area
1		11.051	10361708	98.19
2		14.303	191009	1.81

^{1}H NMR spectrum of *1-((1-(2-Chloro-6-methylphenyl)-1H-1,2,3-triazol-4-yl)methyl)-3-iodo-1H-pyrazolo[3,4-b]pyridine* (23)

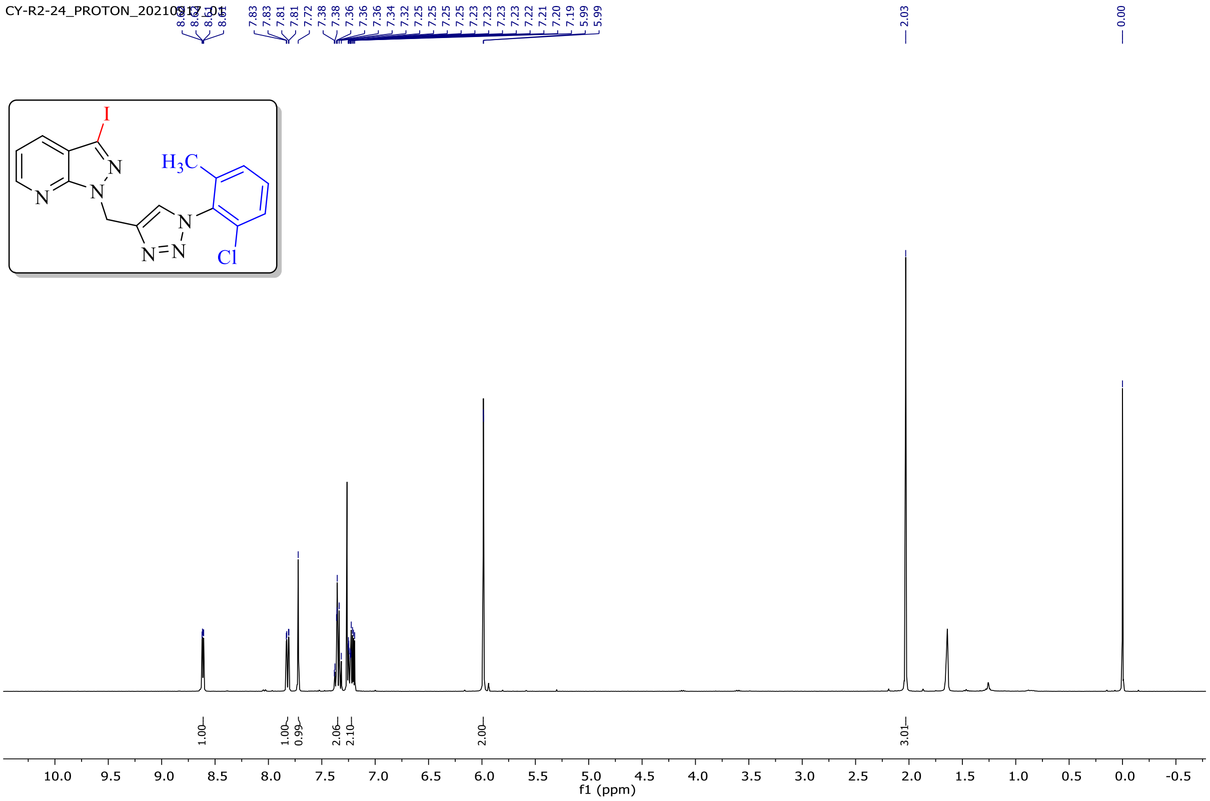

^{13}C NMR spectrum of *1-((1-(2-Chloro-6-methylphenyl)-1H-1,2,3-triazol-4-yl)methyl)-3-iodo-1H-pyrazolo[3,4-b]pyridine* (23)

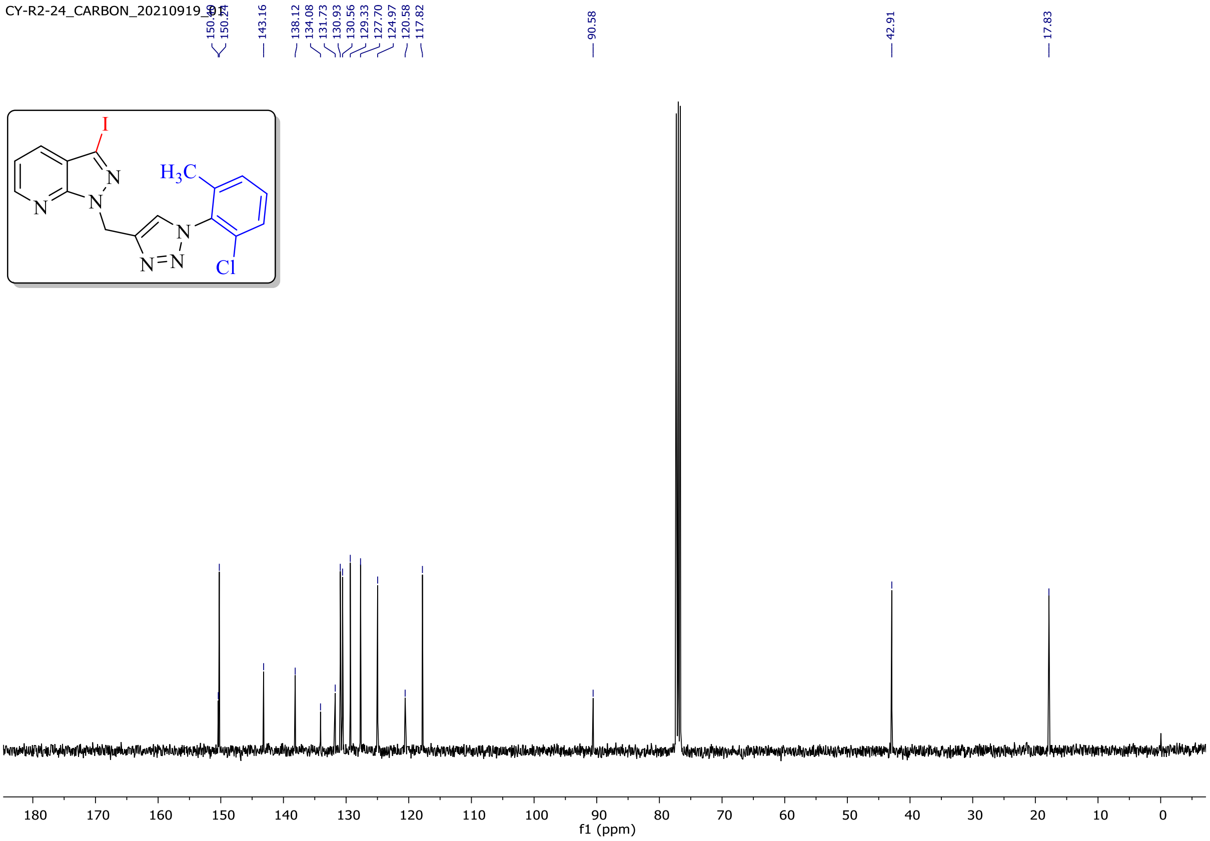

IR spectrum of *1-((1-(2-Chloro-6-methylphenyl)-1H-1,2,3-triazol-4-yl)methyl)-3-iodo-1H-pyrazolo[3,4-b]pyridine* (23)

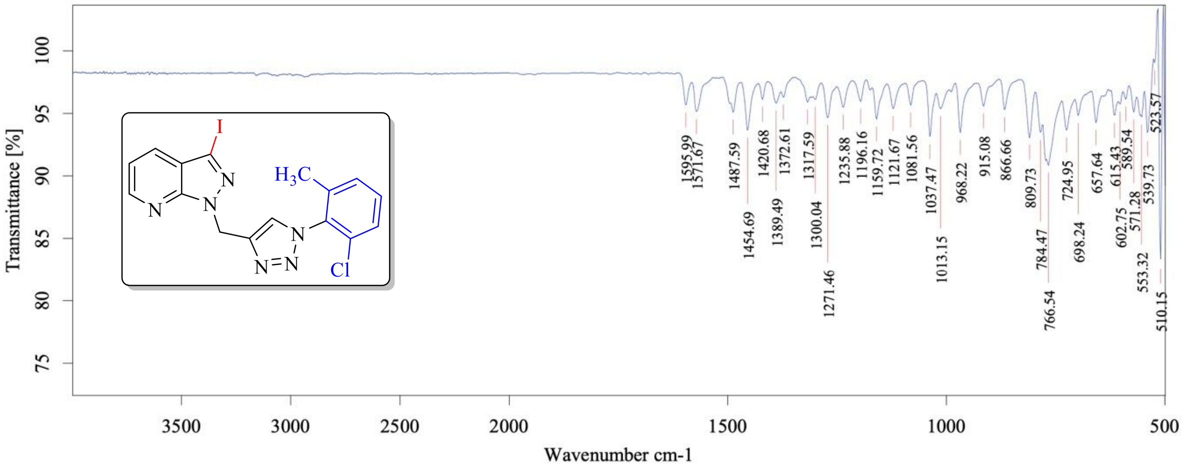

Mass spectrum of *1-((1-(2-Chloro-6-methylphenyl)-1H-1,2,3-triazol-4-yl)methyl)-3-iodo-1H-pyrazolo[3,4-b]pyridine* (23)

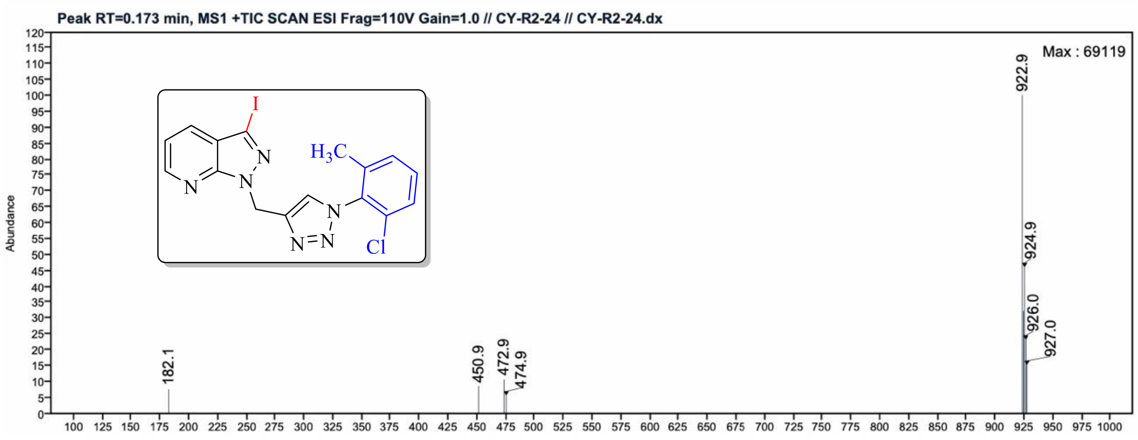

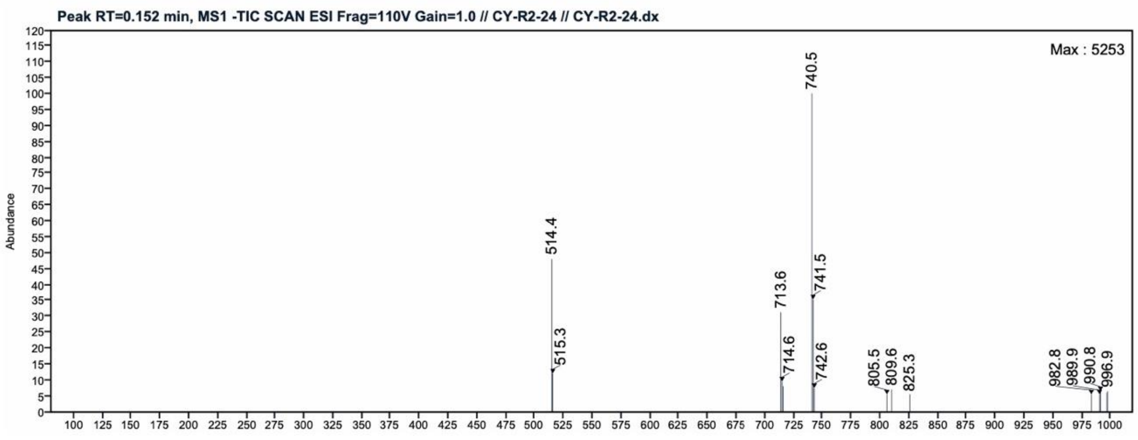

HPLC chromatogram of *1-((1-(2-Chloro-6-methylphenyl)-1H-1,2,3-triazol-4-yl)methyl)-3-iodo-1H-pyrazolo[3,4-b]pyridine* (23)

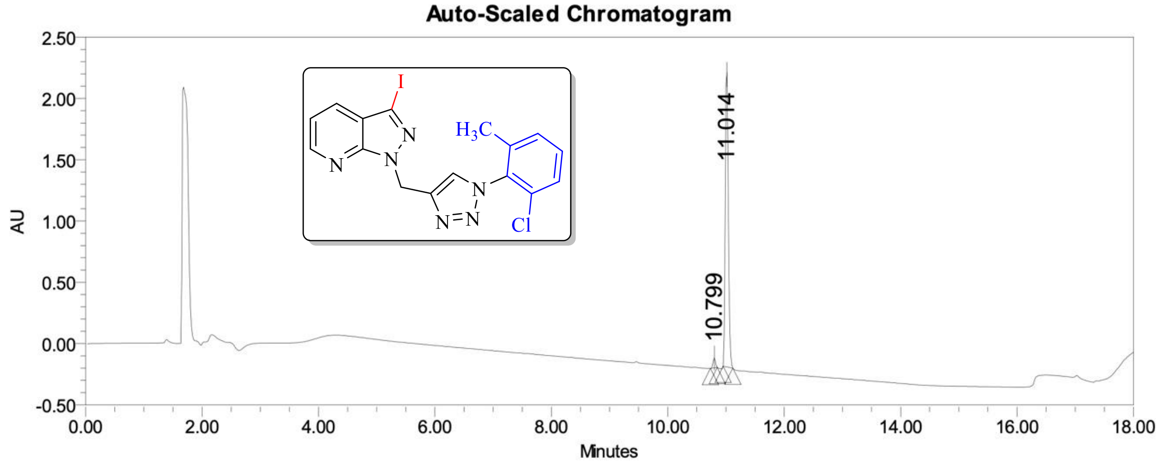

Peak Results

	Name	RT	Area	% Area
1		10.799	243648	2.67
2		11.014	8884521	97.33

^{1}H NMR spectrum of *3-Iodo-1-((1-(o-tolyl)-1H-1,2,3-triazol-4-yl)methyl)-1H-pyrazolo[3,4-b]pyridine* (24)

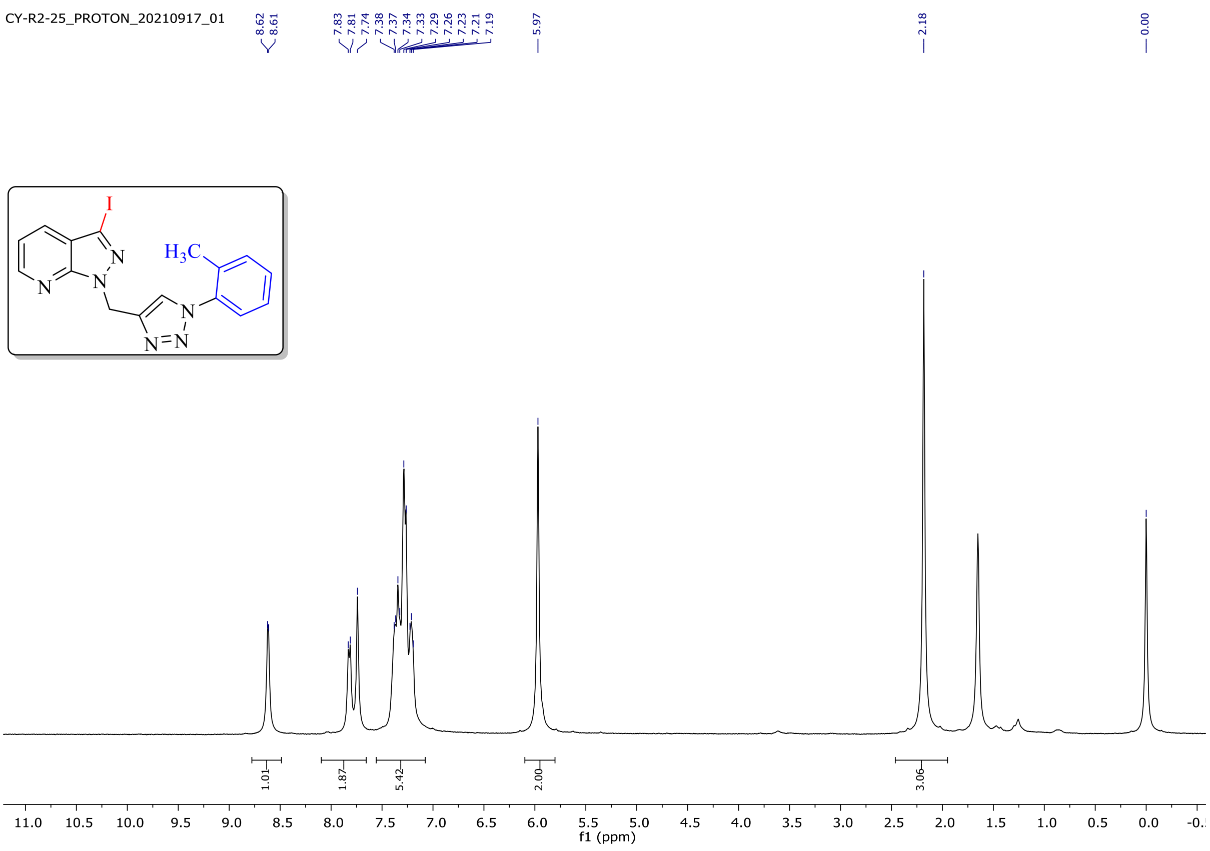

^{13}C NMR spectrum of *3-Iodo-1-((1-(o-tolyl)-1H-1,2,3-triazol-4-yl)methyl)-1H-pyrazolo[3,4-b]pyridine* (24)

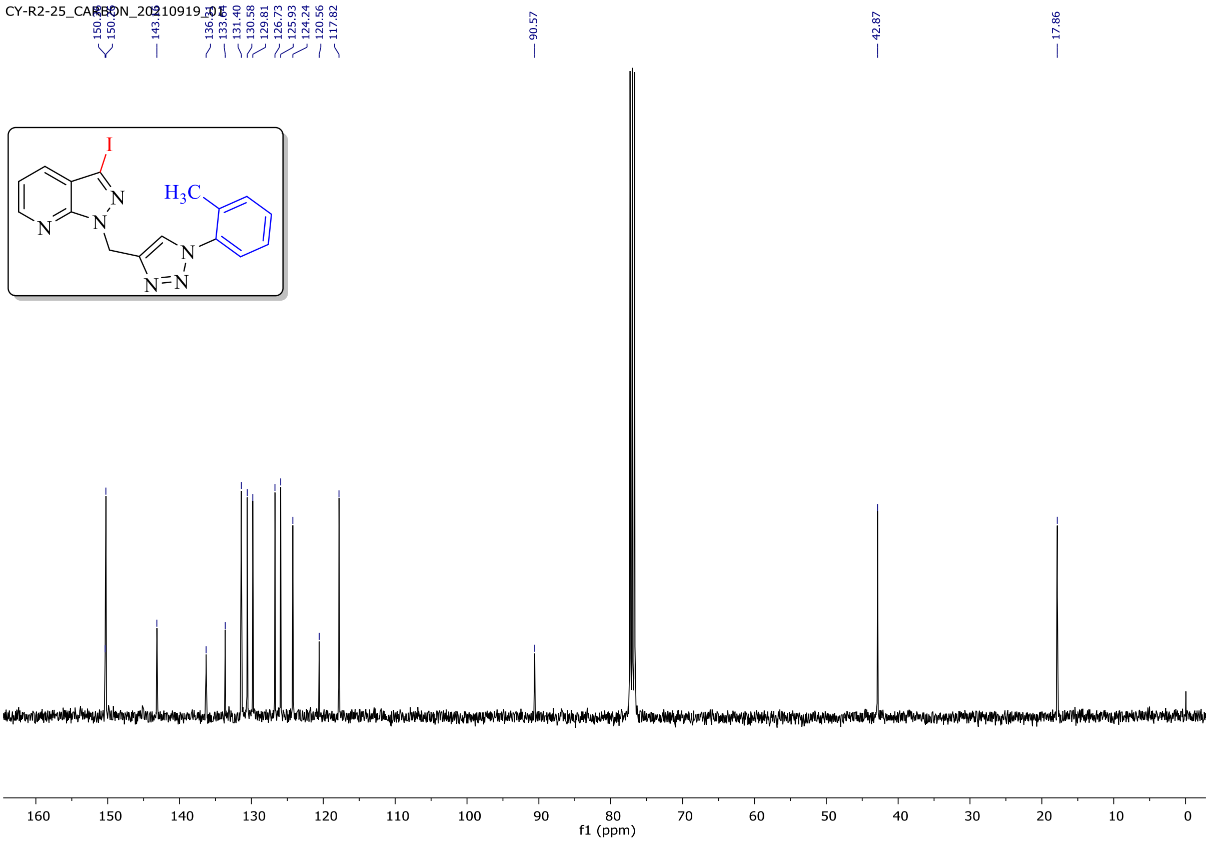

IR spectrum of *3-Iodo-1-((1-(o-tolyl)-1H-1,2,3-triazol-4-yl)methyl)-1H-pyrazolo[3,4-b]pyridine* (24)

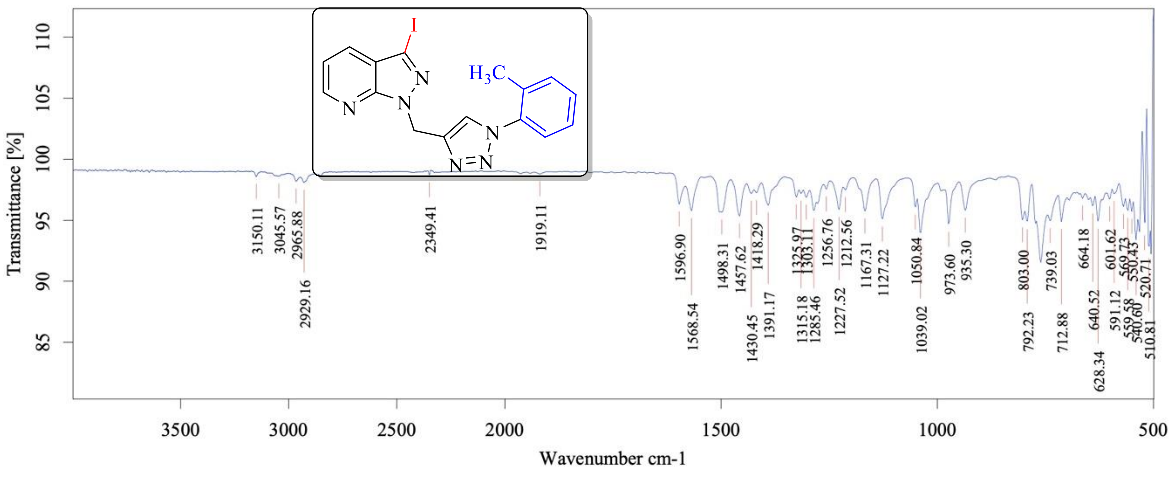

Mass spectrum of *3-Iodo-1-((1-(o-tolyl)-1H-1,2,3-triazol-4-yl)methyl)-1H-pyrazolo[3,4-b]pyridine* (24)

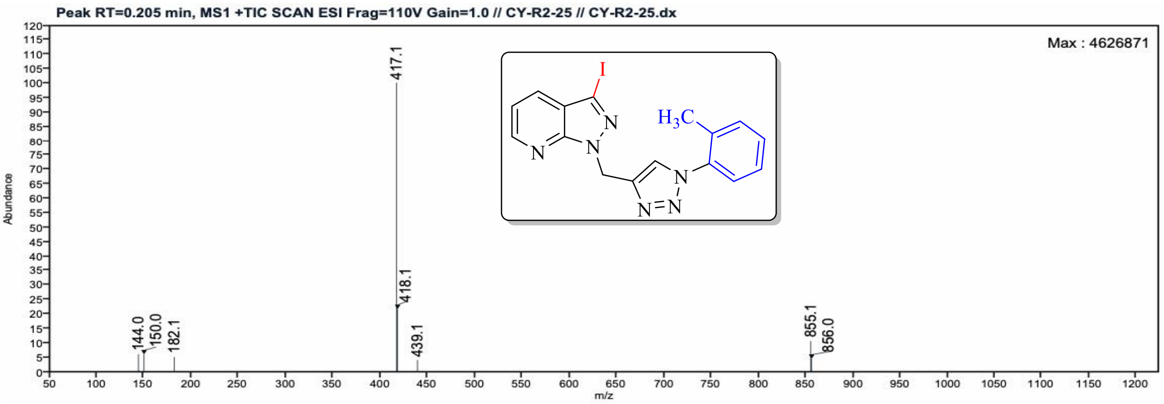

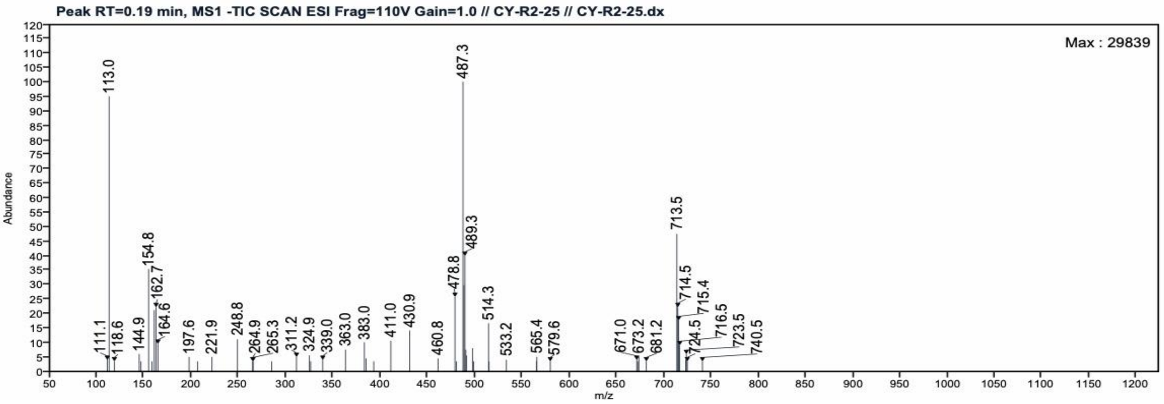

HPLC chromatogram of *3-Iodo-1-((1-(o-tolyl)-1H-1,2,3-triazol-4-yl)methyl)-1H-pyrazolo[3,4-b]pyridine* (24)

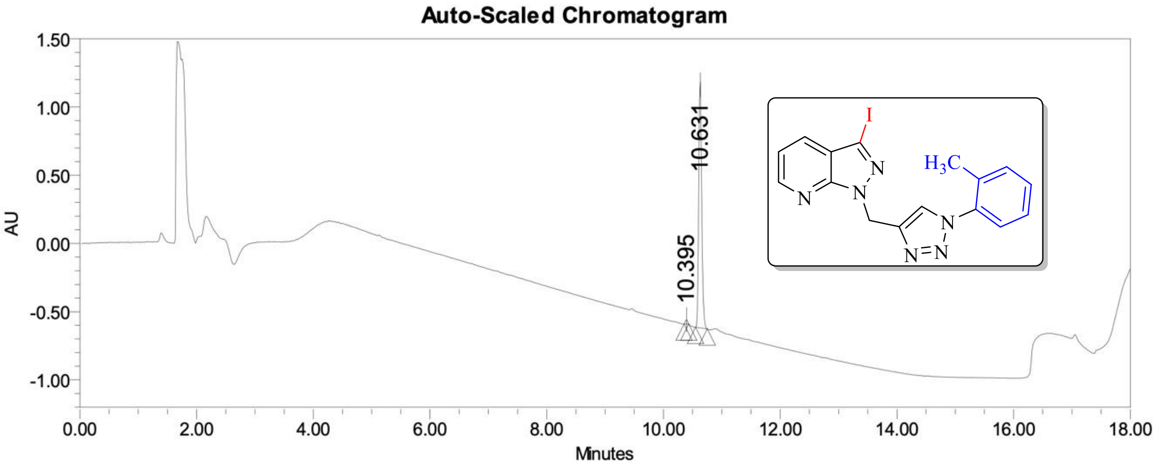

Peak Results

	Name	RT	Area	% Area
1		10.395	87247	1.33
2		10.631	6452798	98.67

^{1}H NMR spectrum of *1-((1-(4-Fluorophenyl)-1H-1,2,3-triazol-4-yl)methyl)-3-iodo-1H-pyrazolo[3,4-b]pyridin* (25)

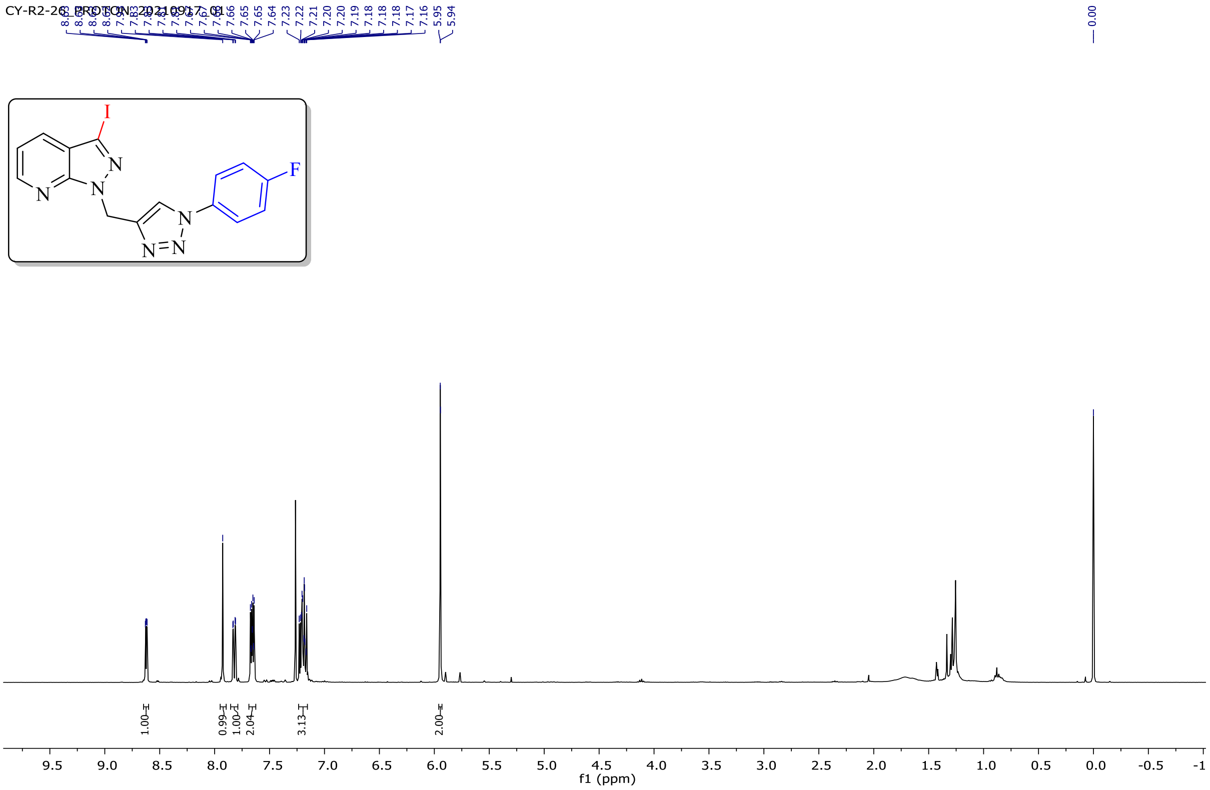

^{13}C NMR spectrum of *1-((1-(4-Fluorophenyl)-1H-1,2,3-triazol-4-yl)methyl)-3-iodo-1H-pyrazolo[3,4-b]pyridin* (25)

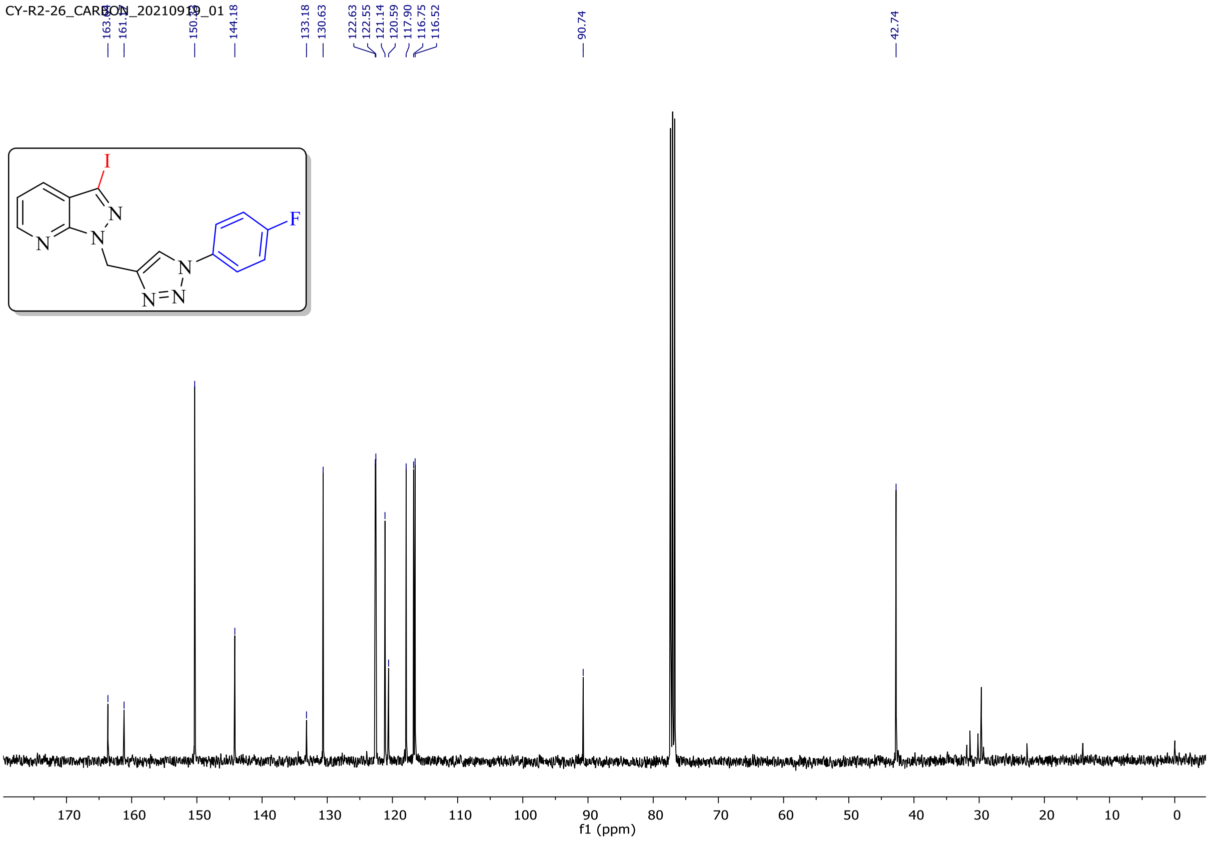

IR spectrum of *1-((1-(4-Fluorophenyl)-1H-1,2,3-triazol-4-yl)methyl)-3-iodo-1H-pyrazolo[3,4-b]pyridin* (25)

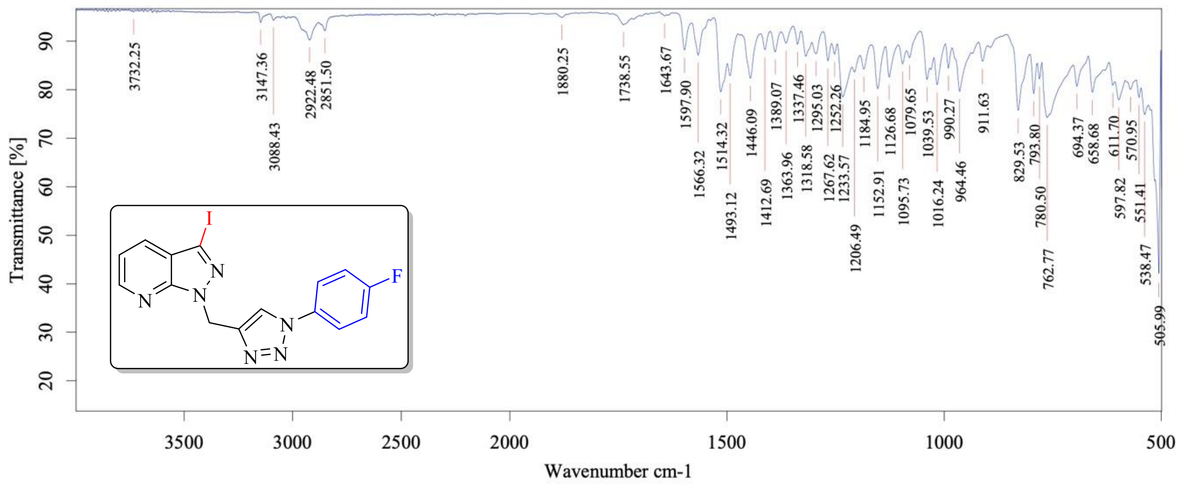

Mass spectrum of *1-((1-(4-Fluorophenyl)-1H-1,2,3-triazol-4-yl)methyl)-3-iodo-1H-pyrazolo[3,4-b]pyridin* (25)

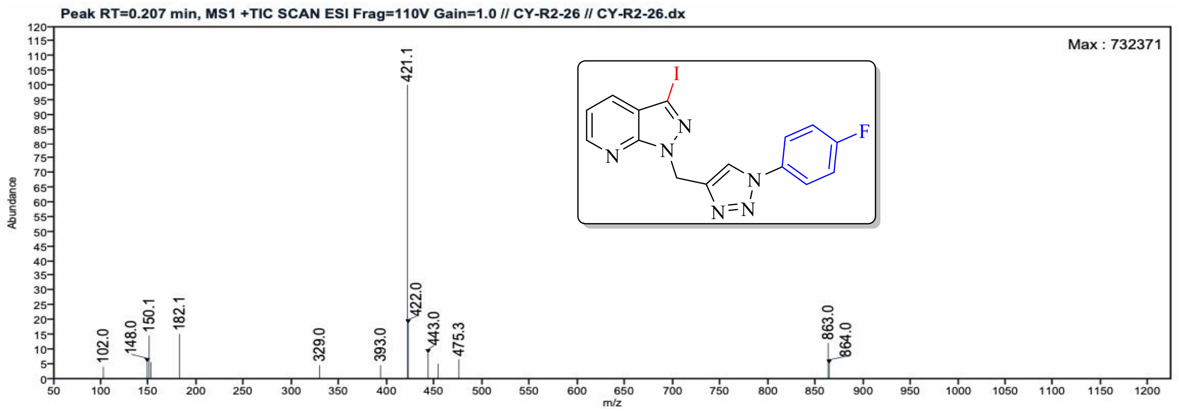

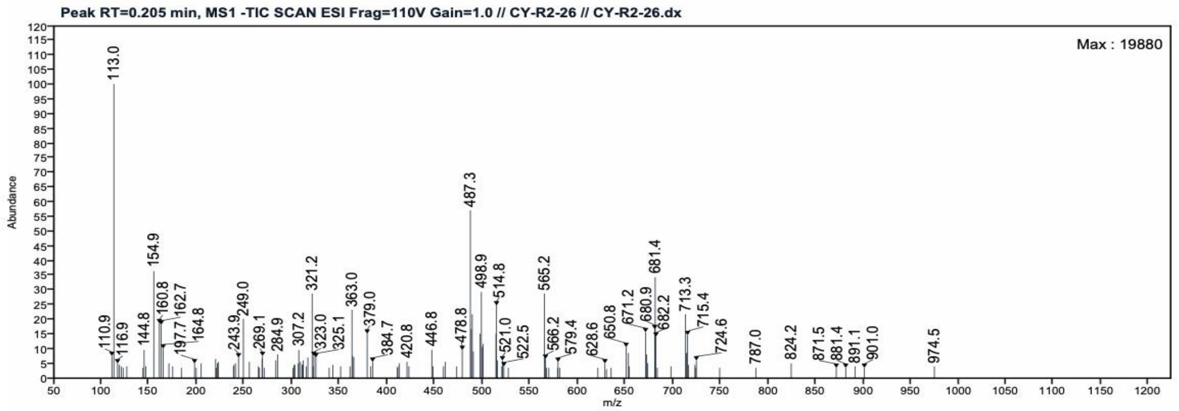

HPLC chromatogram of *1-((1-(4-Fluorophenyl)-1H-1,2,3-triazol-4-yl)methyl)-3-iodo-1H-pyrazolo[3,4-b]pyridin* (25)

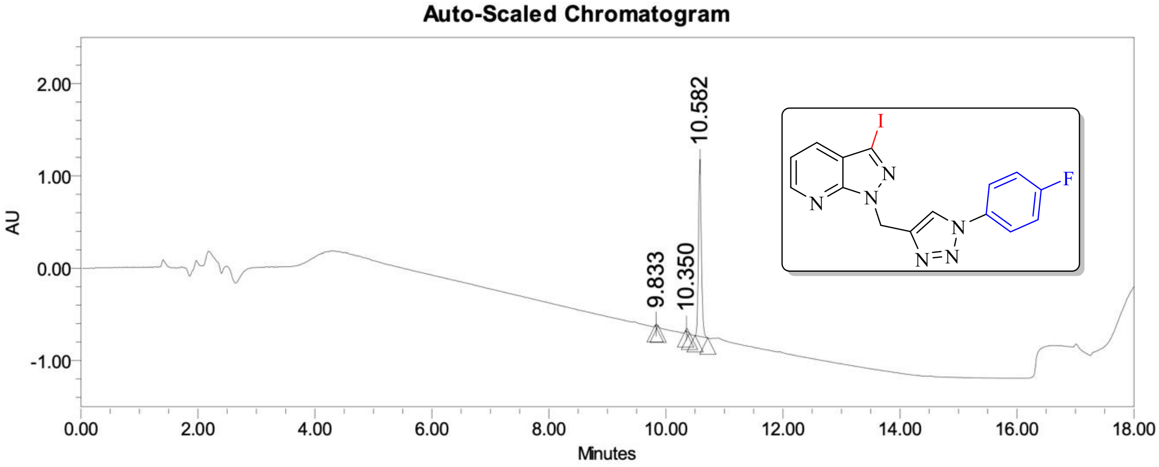

Peak Results

	Name	RT	Area	% Area
1		9.833	34621	0.53
2		10.350	77602	1.18
3		10.582	6481318	98.30

¹H NMR spectrum of *1-((1-(4-Chloro-2-iodophenyl)-1H-1,2,3-triazol-4-yl)methyl)-3-iodo-1H-pyrazolo[3,4-b]pyridine* (26)

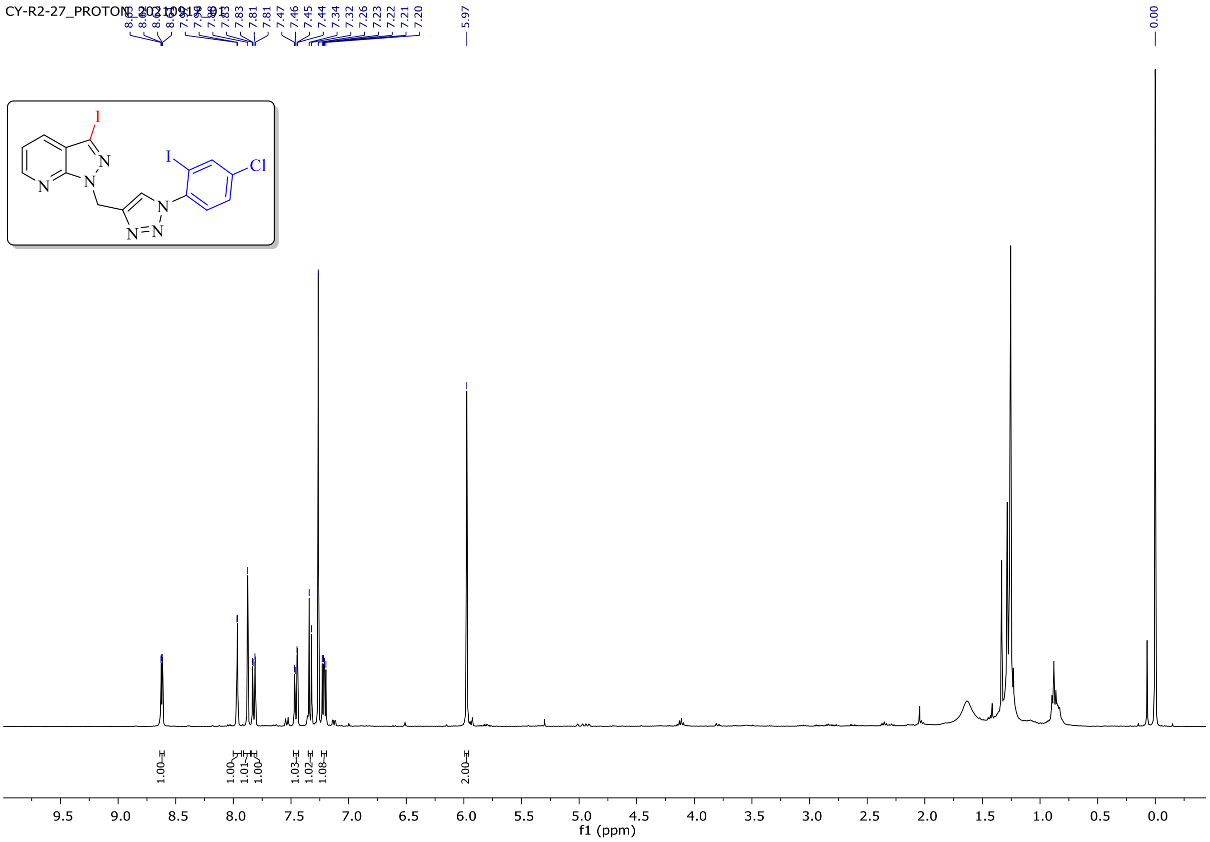

^{13}C NMR spectrum of *1-((1-(4-Chloro-2-iodophenyl)-1H-1,2,3-triazol-4-yl)methyl)-3-iodo-1H-pyrazolo[3,4-b]pyridine* (26)

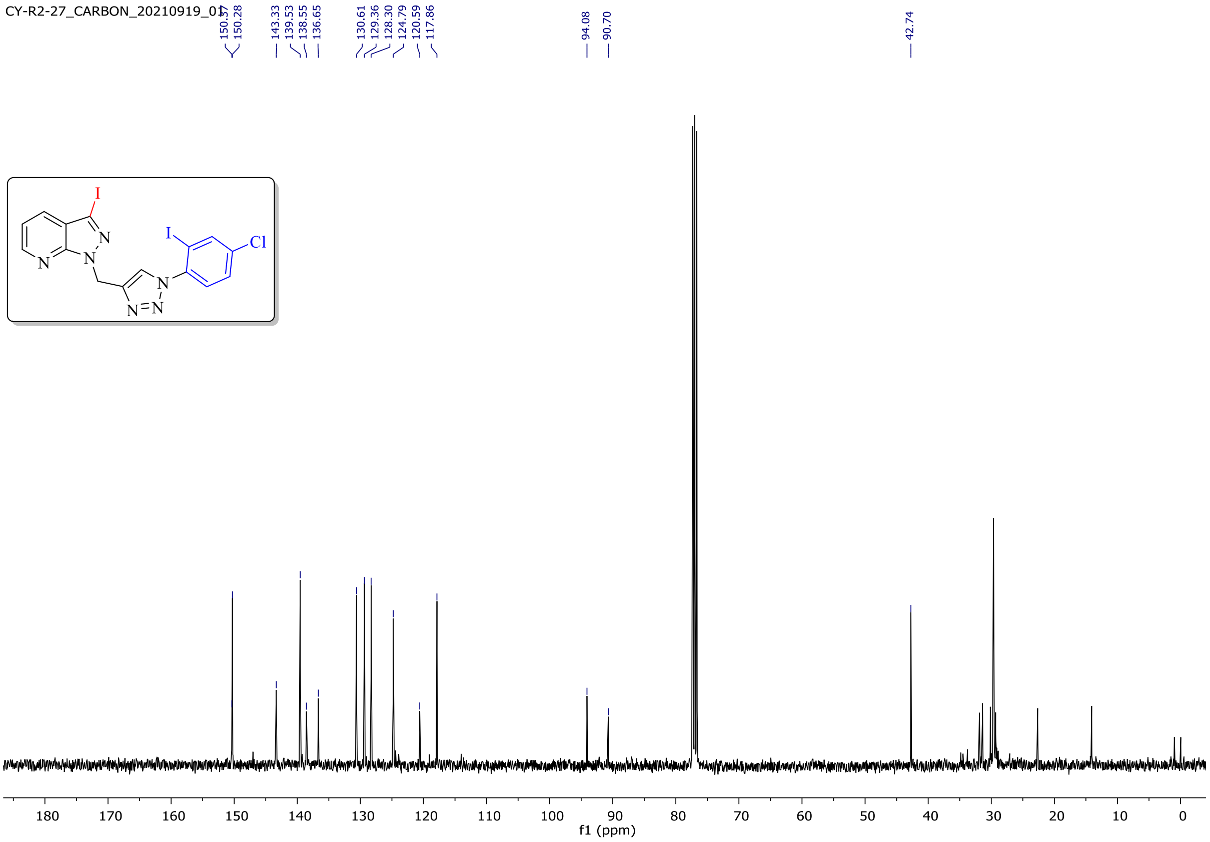

IR spectrum of *1-((1-(4-Chloro-2-iodophenyl)-1H-1,2,3-triazol-4-yl)methyl)-3-iodo-1H-pyrazolo[3,4-b]pyridine* (26)

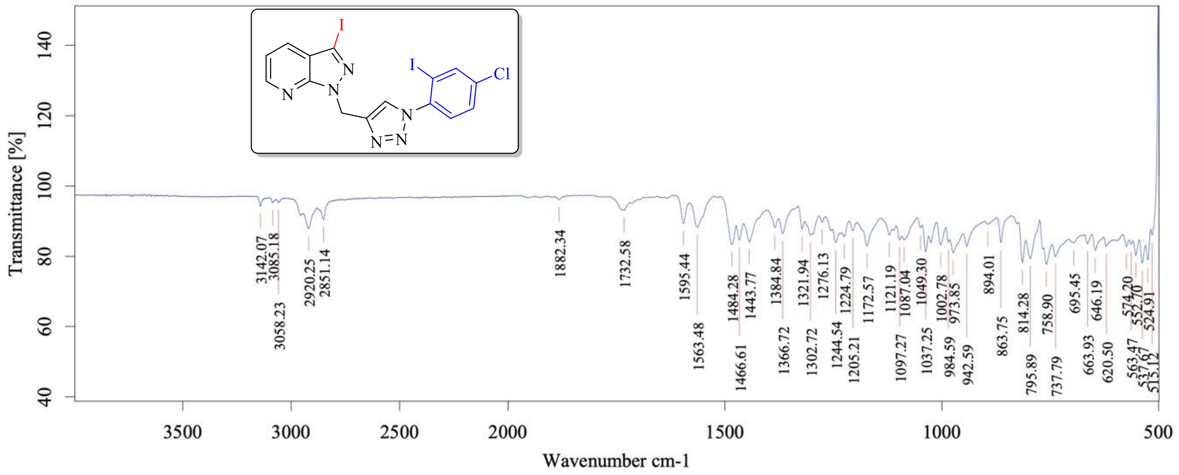

Mass spectrum of *1-((1-(4-Chloro-2-iodophenyl)-1H-1,2,3-triazol-4-yl)methyl)-3-iodo-1H-pyrazolo[3,4-b]pyridine* (26)

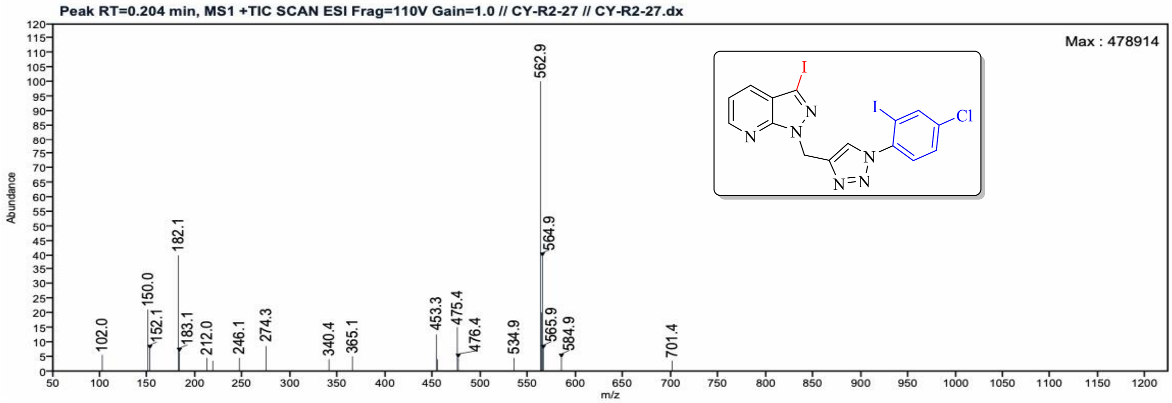

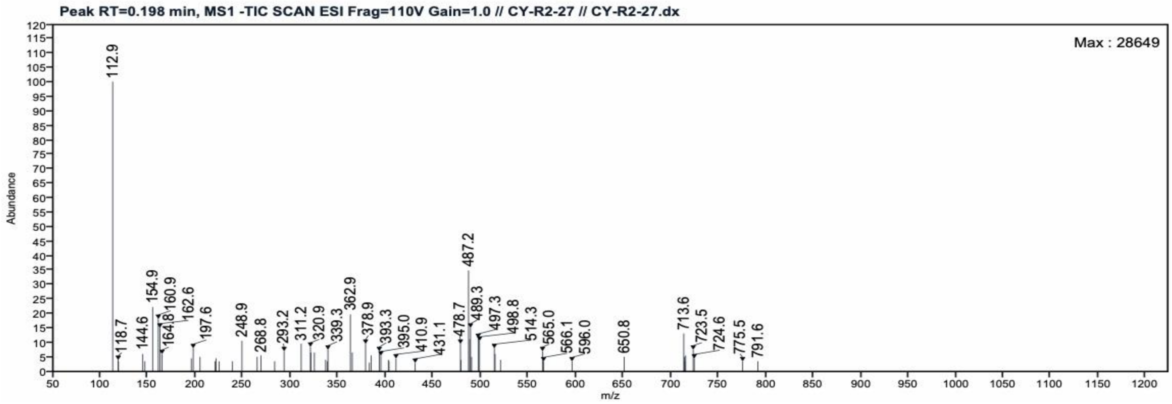

HPLC chromatogram of *1-((1-(4-Chloro-2-iodophenyl)-1H-1,2,3-triazol-4-yl)methyl)-3-iodo-1H-pyrazolo[3,4-b]pyridine* (26)

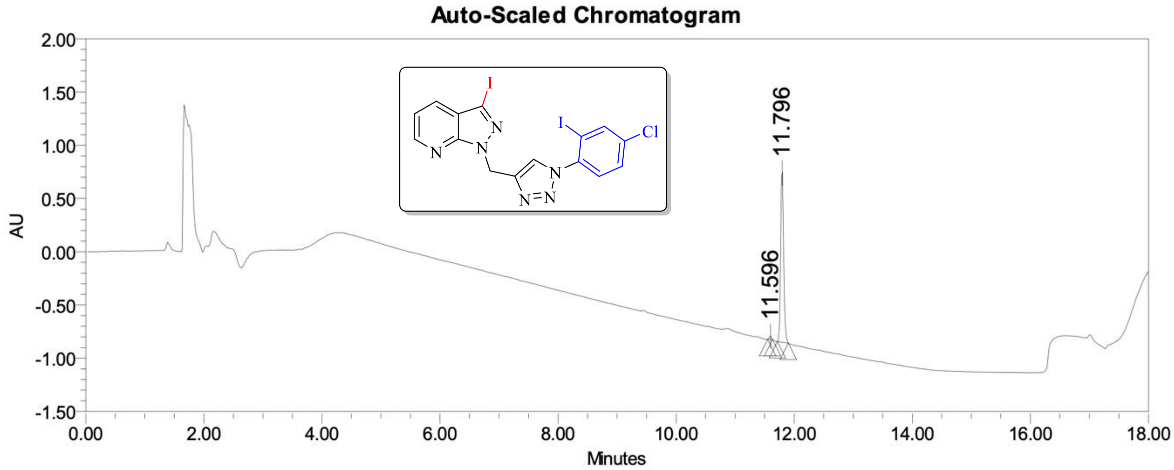

Peak Results

	Name	RT	Area	% Area
1		11.596	80924	1.41
2		11.796	5672987	98.59

^{1}H NMR spectrum of *1-((1-(3-Chlorophenyl)-1H-1,2,3-triazol-4-yl)methyl)-3-iodo-1H-pyrazolo[3,4-b]pyridine* (27)

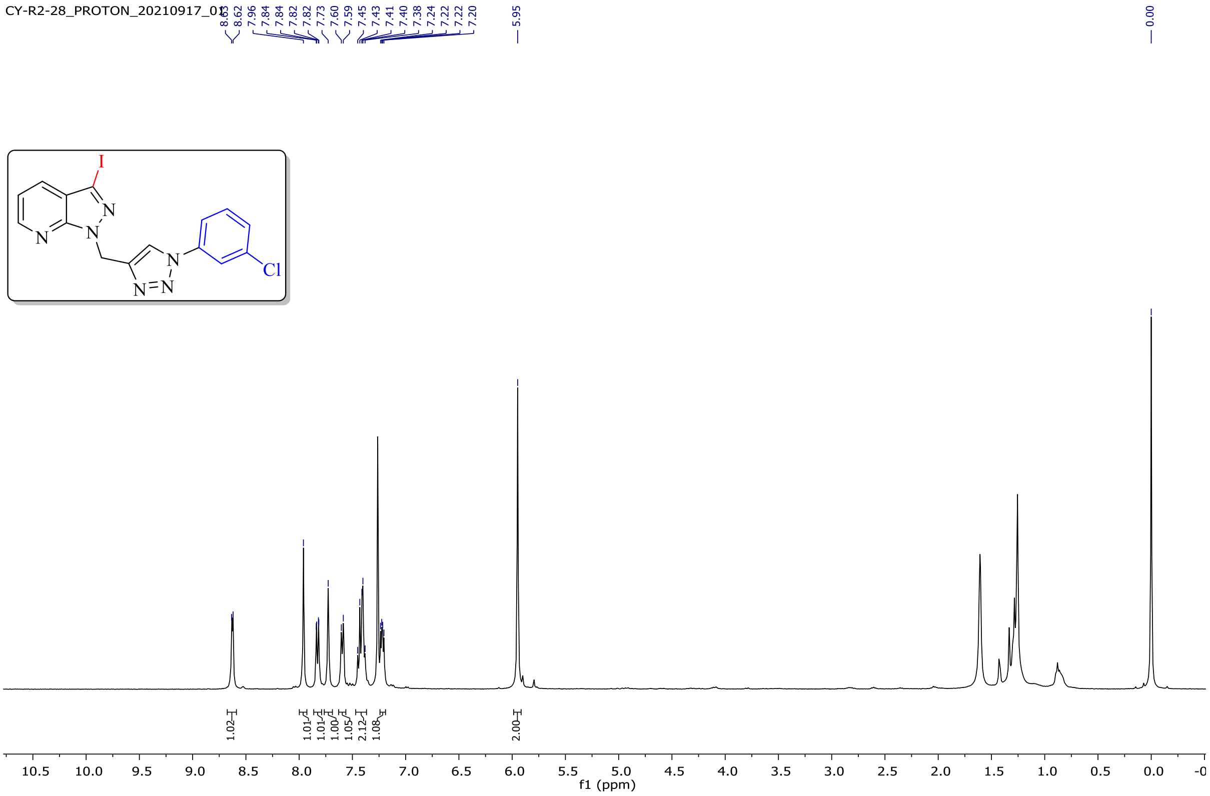

^{13}C NMR spectrum of *1-((1-(3-Chlorophenyl)-1H-1,2,3-triazol-4-yl)methyl)-3-iodo-1H-pyrazolo[3,4-b]pyridine* (27)

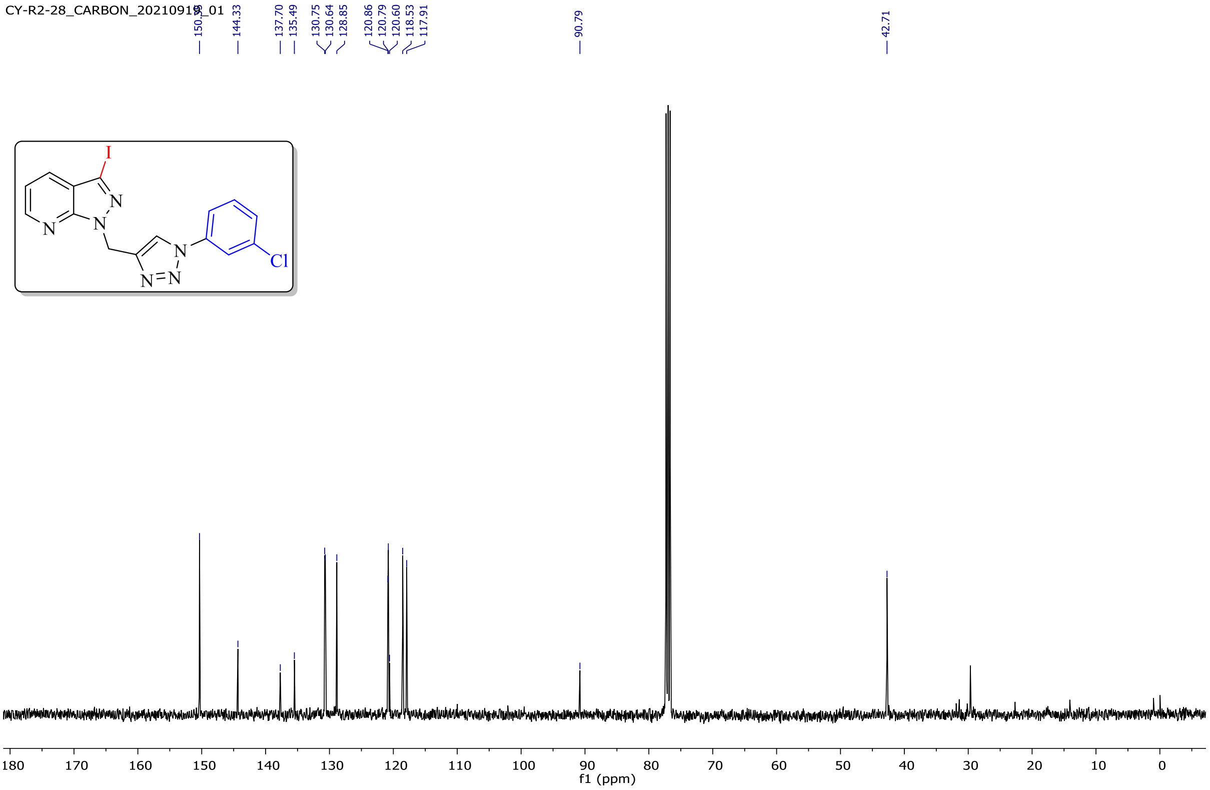

HPLC chromatogram of *1-((1-(3-Chlorophenyl)-1H-1,2,3-triazol-4-yl)methyl)-3-iodo-1H-pyrazolo[3,4-b]pyridine* (27)

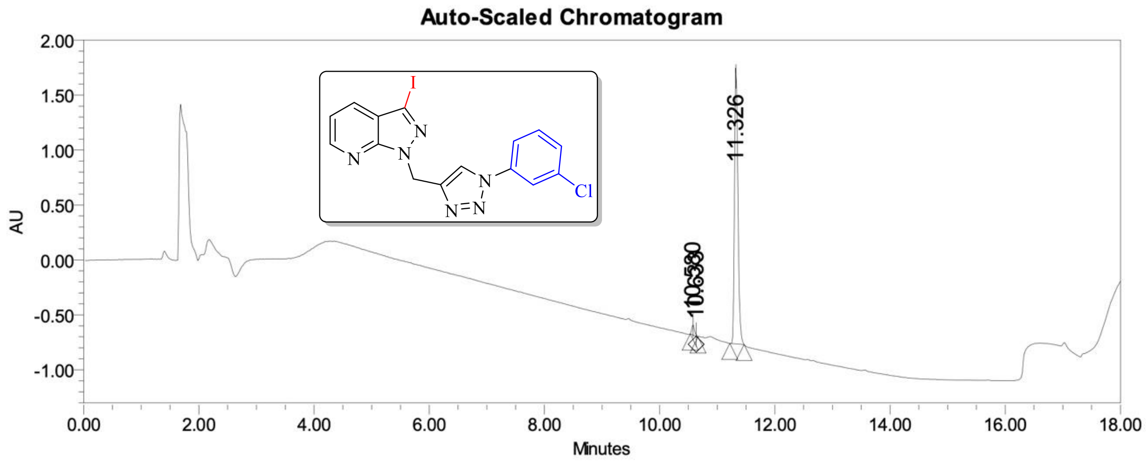

Peak Results

	Name	RT	Area	% Area
1		10.580	300138	2.84
2		10.633	16729	0.16
3		11.326	10261032	97.00

^{1}H NMR spectrum of *(1-(2-Chloro-5-methylphenyl)-1H-1,2,3-triazol-4-yl)methyl1-((1-(2-chloro-6-methylphenyl) -1H-1,2,3-triazol-4-yl)methyl)-1H-pyrazolo[3,4-b]pyridine-3-carboxylate* (28)

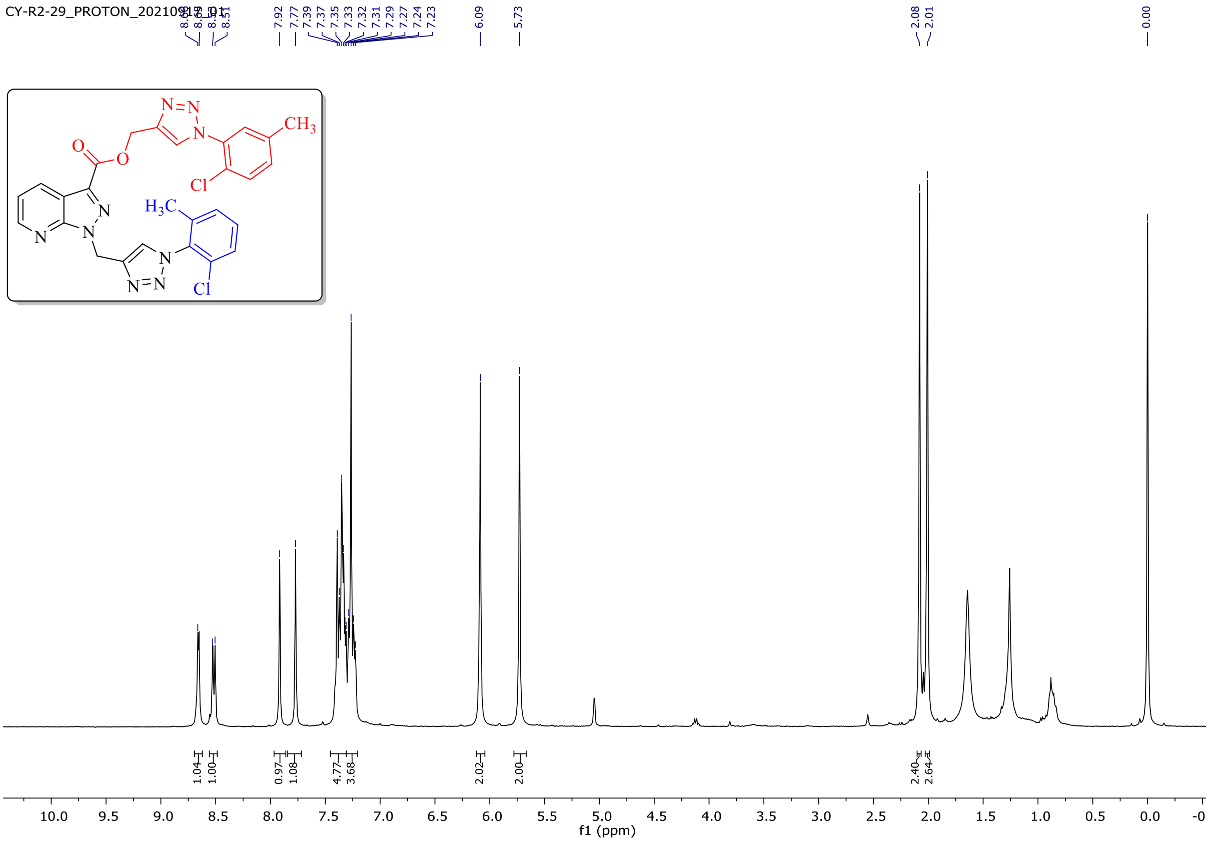

^{13}C NMR spectrum of *(1-(2-Chloro-5-methylphenyl)-1H-1,2,3-triazol-4-yl)methyl1-((1-(2-chloro-6-methylphenyl) -1H-1,2,3-triazol-4-yl)methyl)-1H-pyrazolo[3,4-b]pyridine-3-carboxylate* (28)

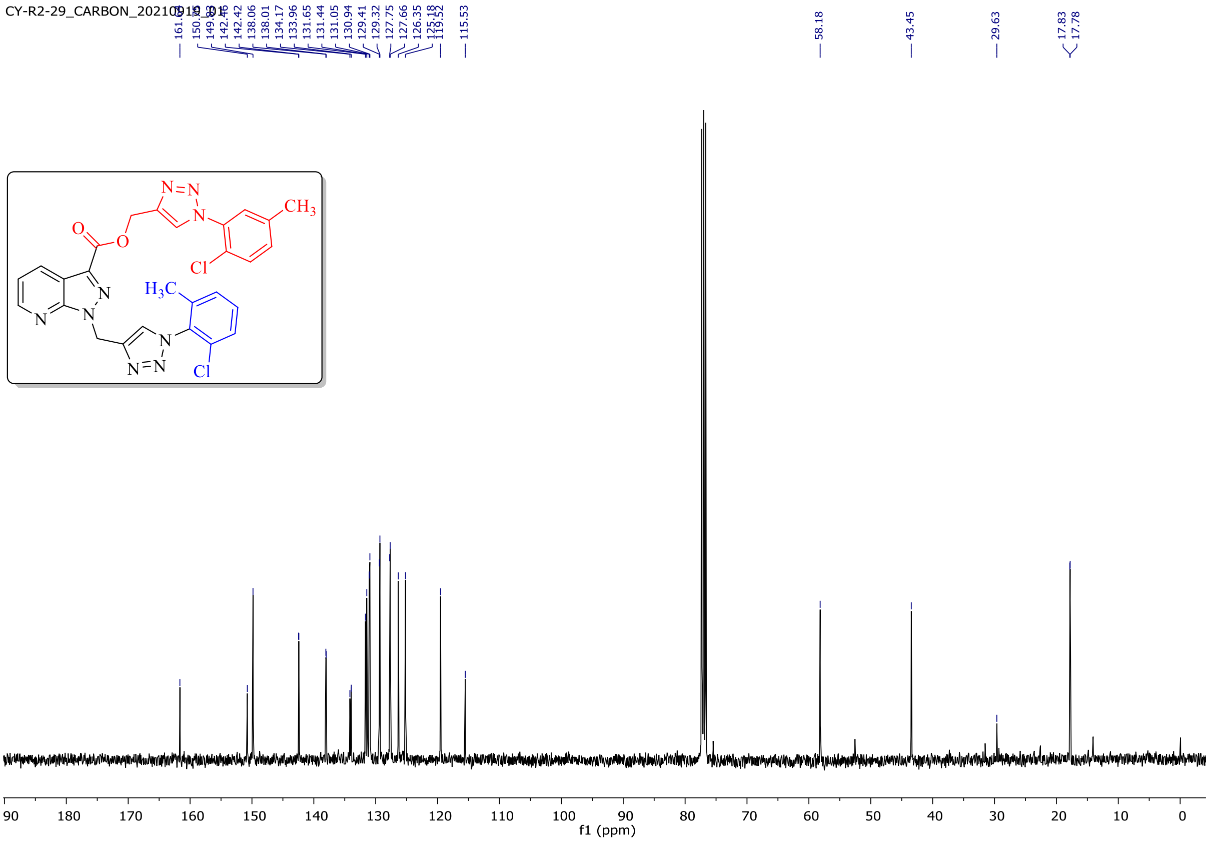

IR spectrum of *(1-(2-Chloro-5-methylphenyl)-1H-1,2,3-triazol-4-yl)methyl1-((1-(2-chloro-6-methylphenyl) -1H-1,2,3-triazol-4-yl)methyl)-1H-pyrazolo[3,4-b]pyridine-3-carboxylate* (28)

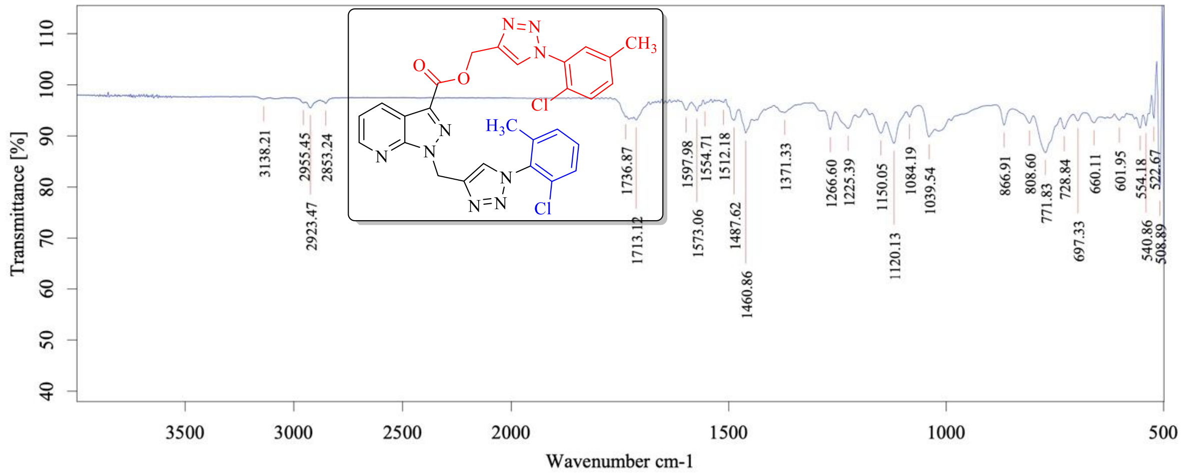

Mass spectrum of *(1-(2-Chloro-5-methylphenyl)-1H-1,2,3-triazol-4-yl)methyl1-((1-(2-chloro-6-methylphenyl) -1H-1,2,3-triazol-4-yl)methyl)-1H-pyrazolo[3,4-b]pyridine-3-carboxylate* (28)

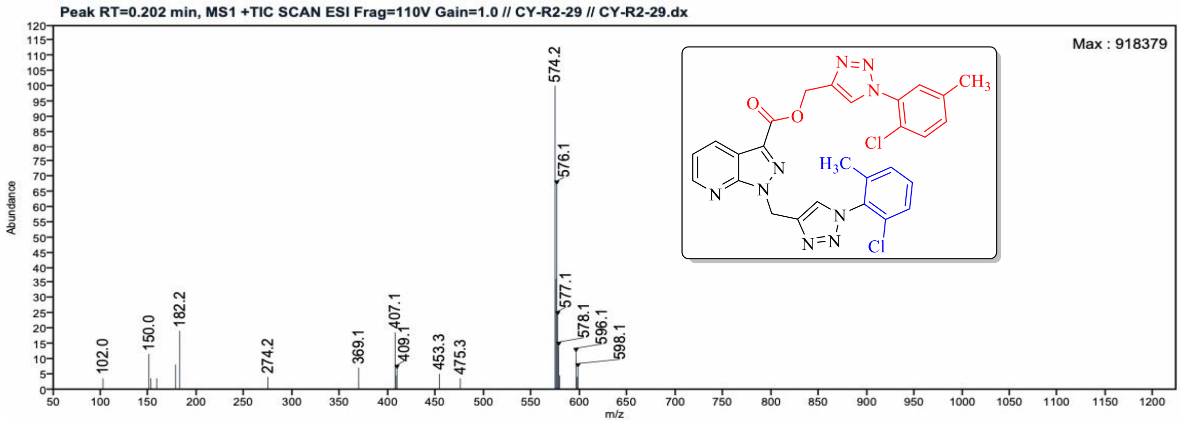

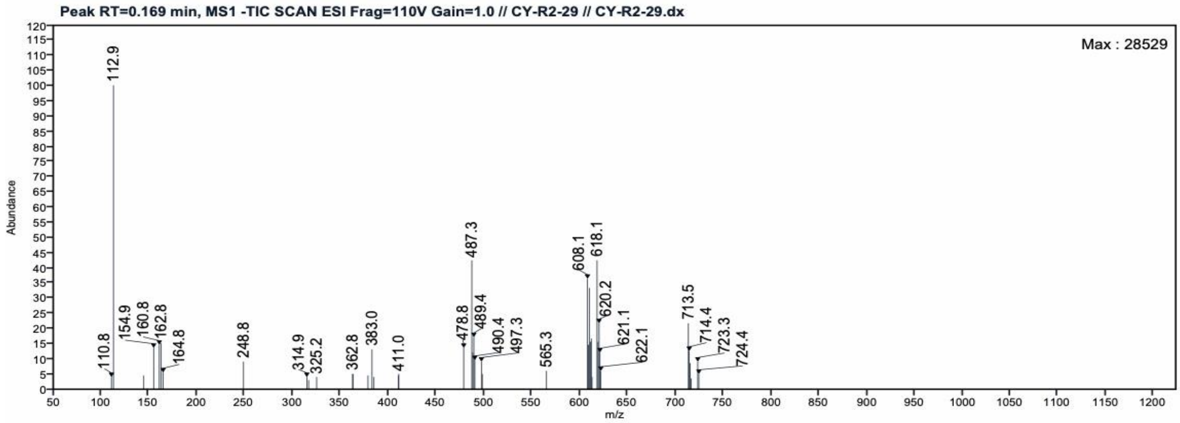

HPLC chromatogram of *(1-(2-Chloro-5-methylphenyl)-1H-1,2,3-triazol-4-yl)methyl1-((1-(2-chloro-6-methylphenyl) -1H-1,2,3-triazol-4-yl)methyl)-1H-pyrazolo[3,4-b]pyridine-3-carboxylate* (28)

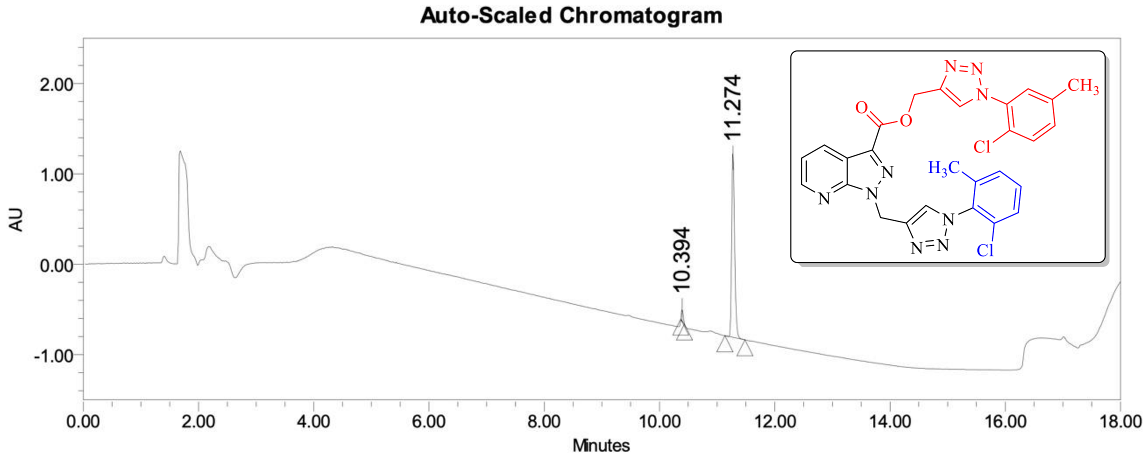

Peak Results

	Name	RT	Area	% Area
1		10.394	289952	3.86
2		11.274	7227763	96.14

^{1}H NMR spectrum of *(1-(4-Fluorophenyl)-1H-1,2,3-triazol-4-yl)methyl 1-((1-(4-fluorophenyl)-1H-1,2,3-triazol-4-yl)methyl)-1H-pyrazolo[3,4-b]pyridine-3-carboxylate* (29)

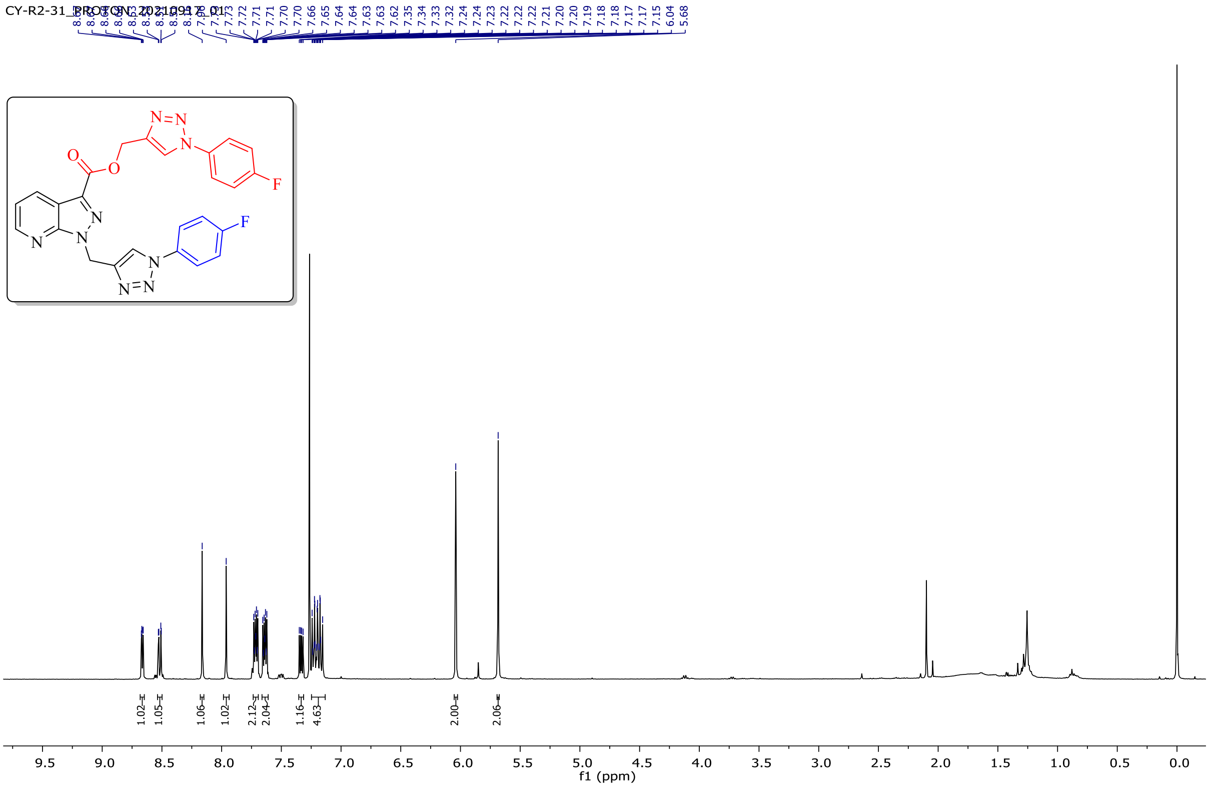

^{13}C NMR spectrum of *(1-(4-Fluorophenyl)-1H-1,2,3-triazol-4-yl)methyl 1-((1-(4-fluorophenyl)-1H-1,2,3-triazol-4-yl)methyl)-1H-pyrazolo[3,4-b]pyridine-3-carboxylate* (29)

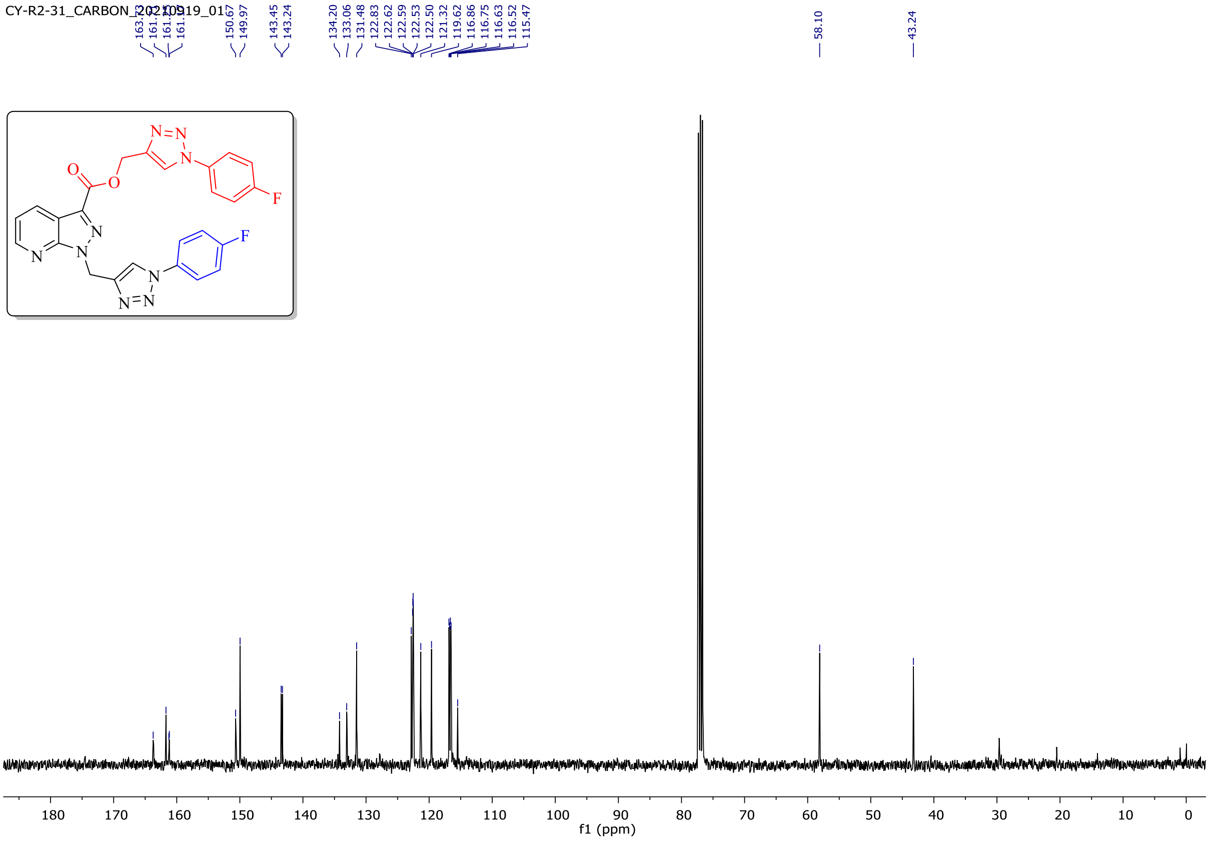

IR spectrum of *(1-(4-Fluorophenyl)-1H-1,2,3-triazol-4-yl)methyl 1-((1-(4-fluorophenyl)-1H-1,2,3-triazol-4-yl)methyl)-1H-pyrazolo[3,4-b]pyridine-3-carboxylate* (29)

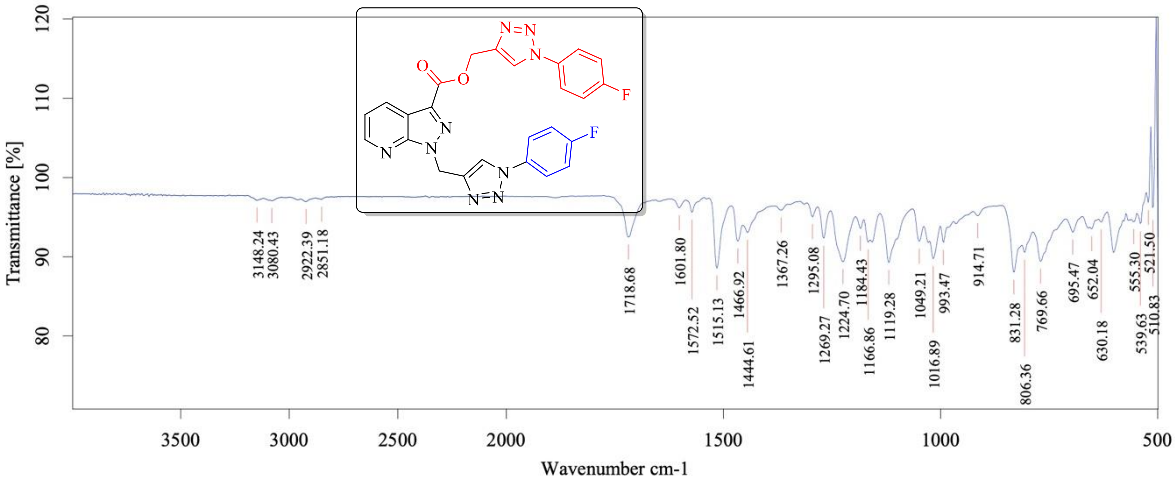

Mass spectrum of *(1-(4-Fluorophenyl)-1H-1,2,3-triazol-4-yl)methyl 1-((1-(4-fluorophenyl)-1H-1,2,3-triazol-4-yl)methyl)-1H-pyrazolo[3,4-b]pyridine-3-carboxylate* (29)

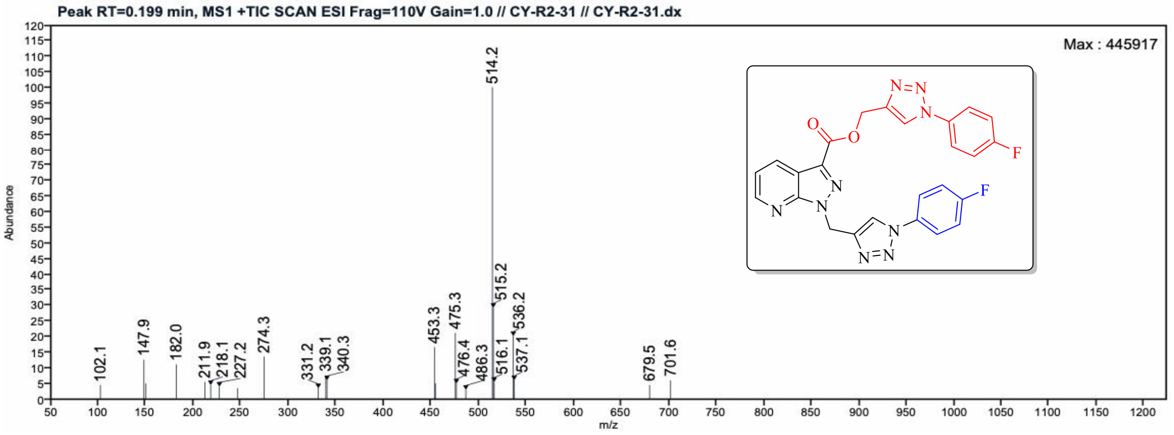

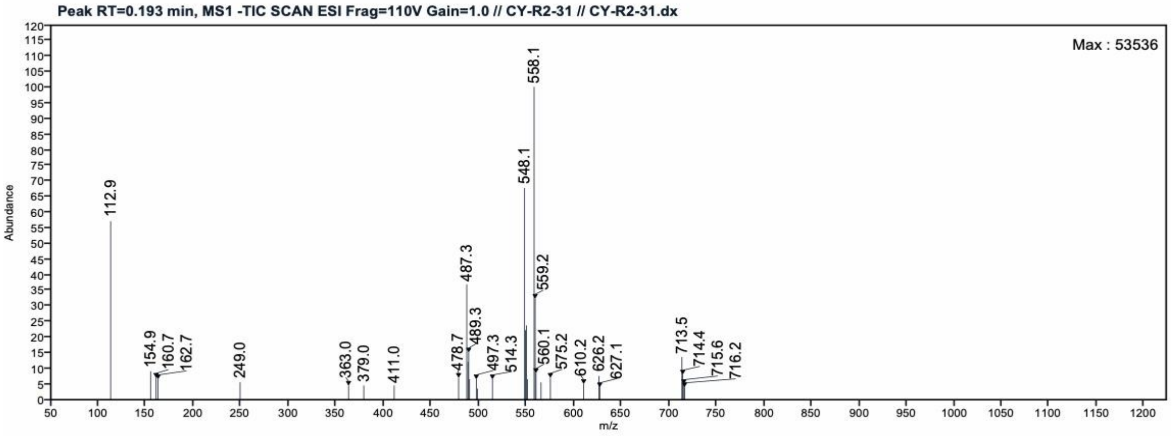

HPLC chromatogram of *(1-(4-Fluorophenyl)-1H-1,2,3-triazol-4-yl)methyl 1-((1-(4-fluorophenyl)-1H-1,2,3-triazol-4-yl)methyl)-1H-pyrazolo[3,4-b]pyridine-3-carboxylate* (29)

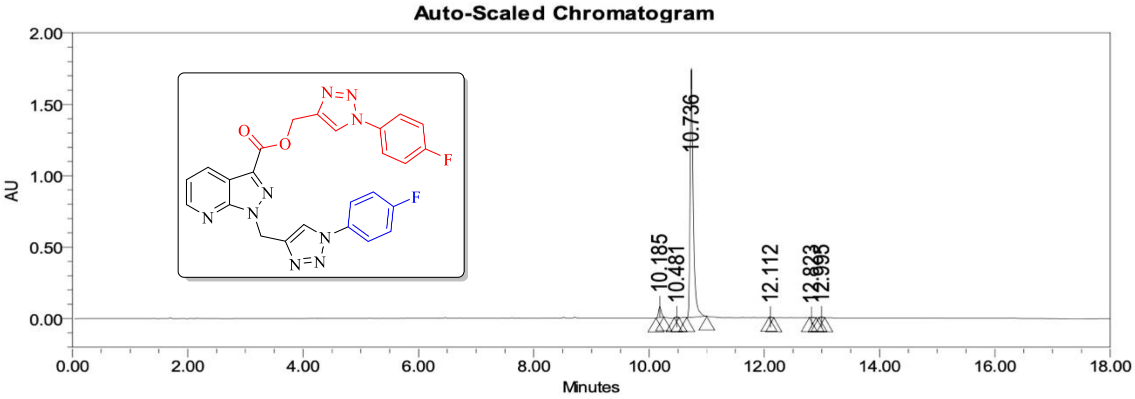

Peak Results

	Name	RT	Area	% Area
1		10.185	242899	3.66
2		10.481	20449	0.31
3		10.736	6330986	95.27
4		12.112	11445	0.17
5		12.823	15600	0.23

^{1}H NMR spectrum of *(1-(4-Chloro-2-iodophenyl)-1H-1,2,3-triazol-4-yl)methyl 1-((1-(4-chloro-2-iodophenyl)-1H-1,2,3-triazol-4-yl)methyl)-1H-pyrazolo[3,4-b]pyridine-3-carboxylate* (30)

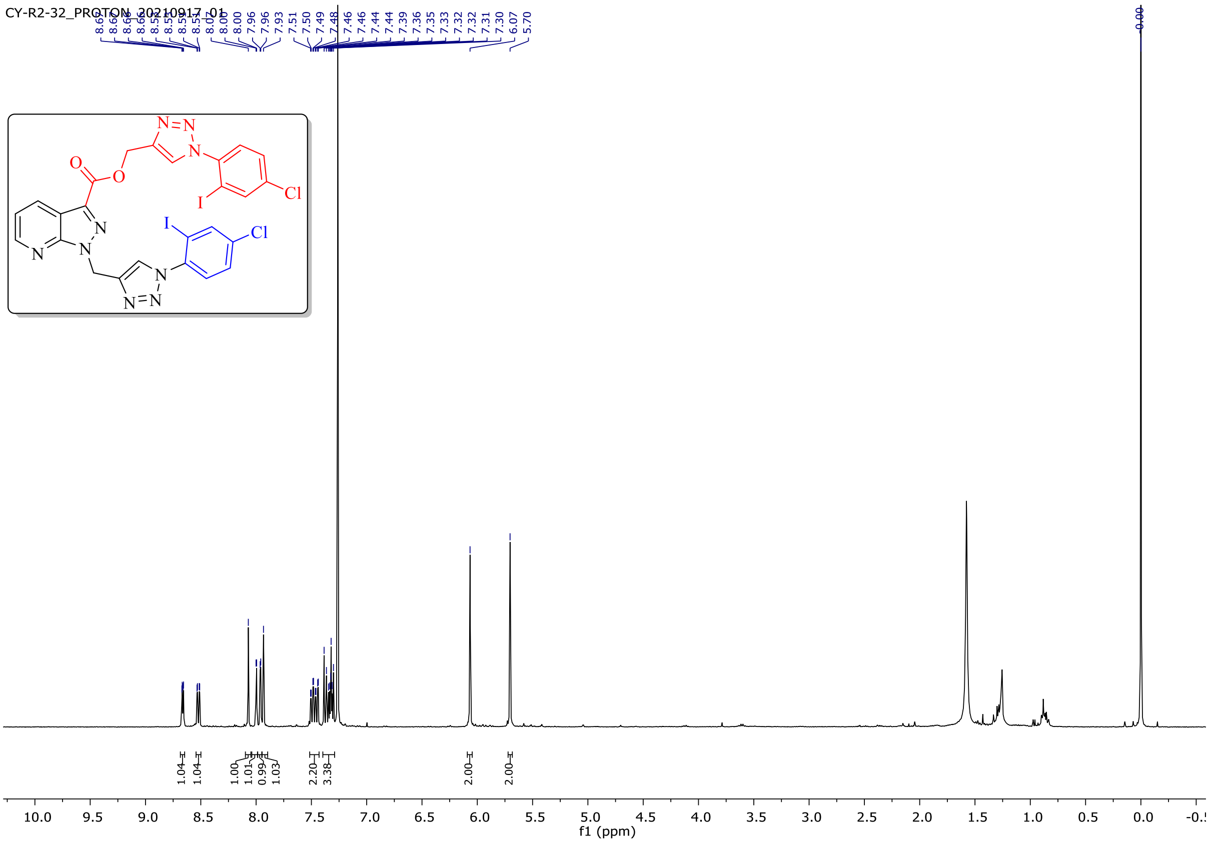

^{13}C NMR spectrum of *(1-(4-Chloro-2-iodophenyl)-1H-1,2,3-triazol-4-yl)methyl 1-((1-(4-chloro-2-iodophenyl)-1H-1,2,3-triazol-4-yl)methyl)-1H-pyrazolo[3,4-b]pyridine-3-carboxylate* (30)

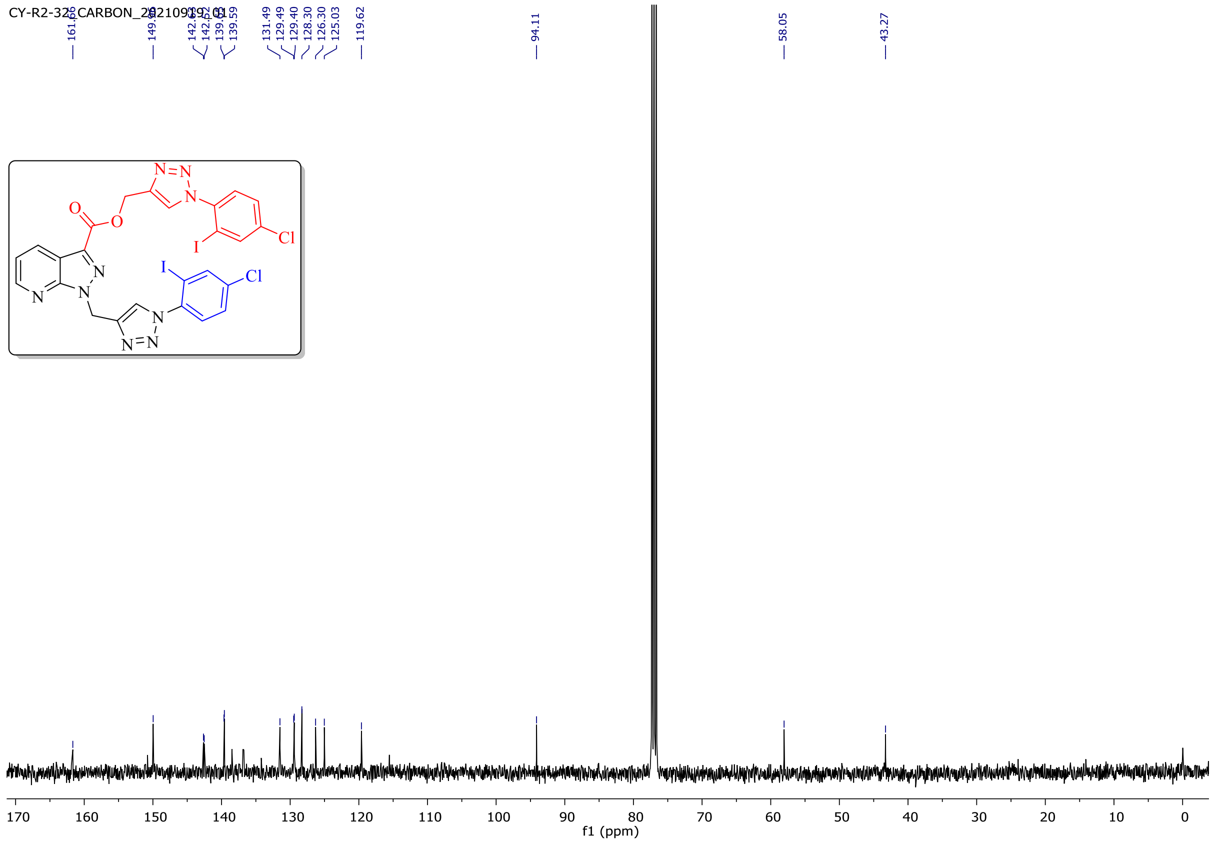

IR spectrum of *(1-(4-Chloro-2-iodophenyl)-1H-1,2,3-triazol-4-yl)methyl 1-((1-(4-chloro-2-iodophenyl)-1H-1,2,3-triazol-4-yl)methyl)-1H-pyrazolo[3,4-b]pyridine-3-carboxylate* (30)

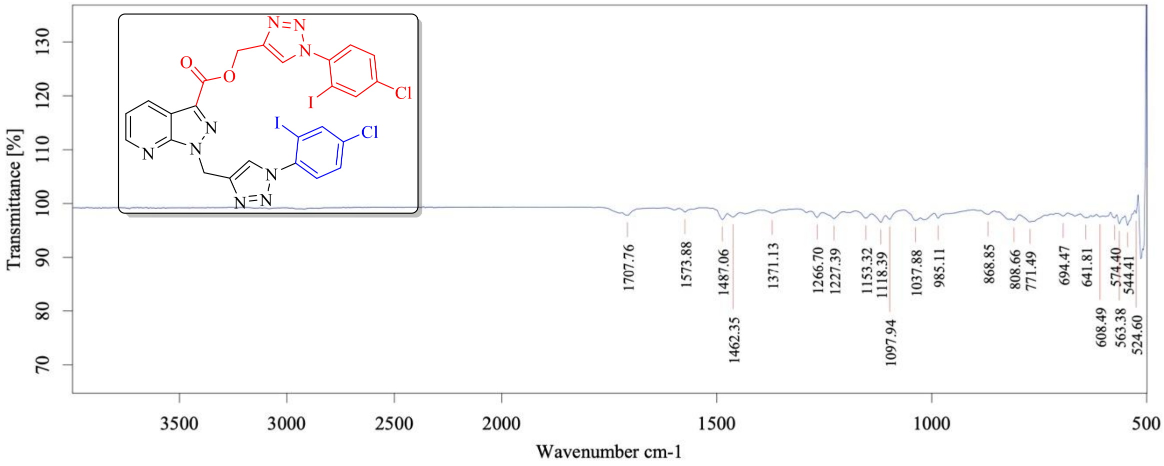

Mass spectrum of *(1-(4-Chloro-2-iodophenyl)-1H-1,2,3-triazol-4-yl)methyl 1-((1-(4-chloro-2-iodophenyl)-1H-1,2,3-triazol-4-yl)methyl)-1H-pyrazolo[3,4-b]pyridine-3-carboxylate* (30)

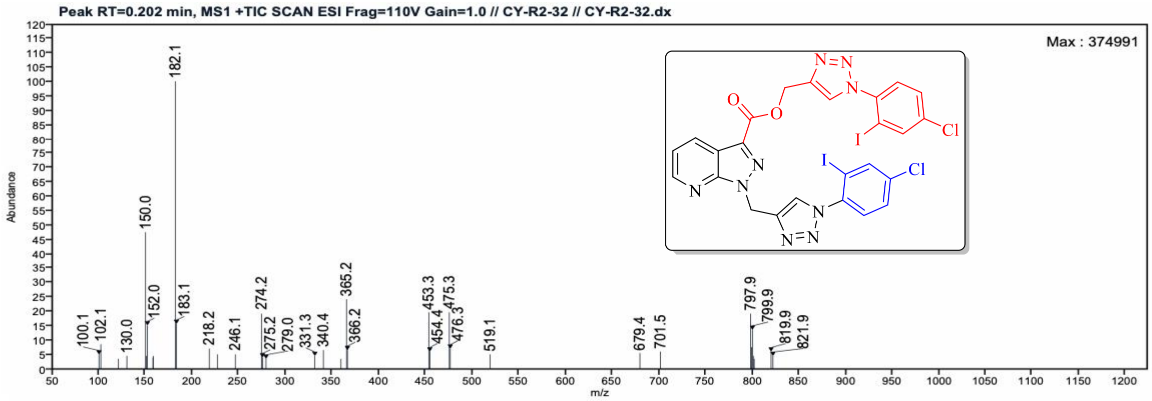

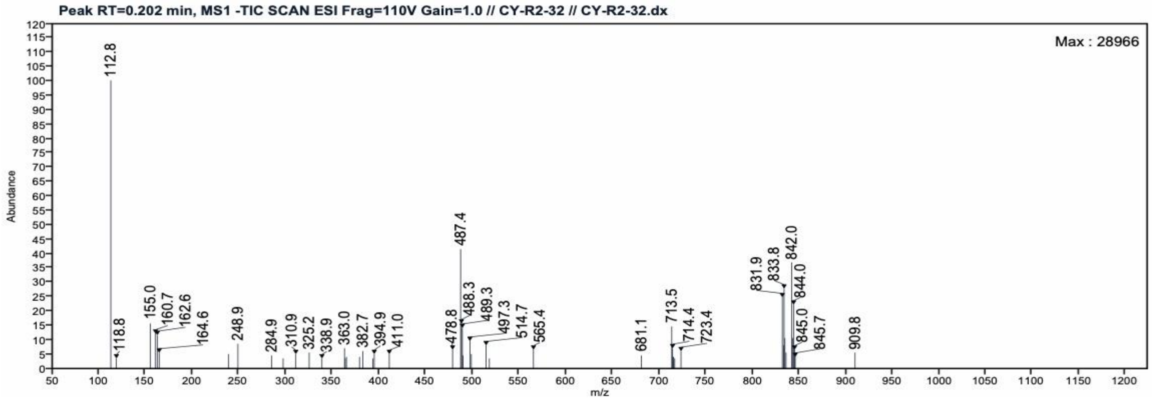

HPLC chromatogram of *(1-(4-Chloro-2-iodophenyl)-1H-1,2,3-triazol-4-yl)methyl 1-((1-(4-chloro-2-iodophenyl)-1H-1,2,3-triazol-4-yl)methyl)-1H-pyrazolo[3,4-b]pyridine-3-carboxylate* (30)

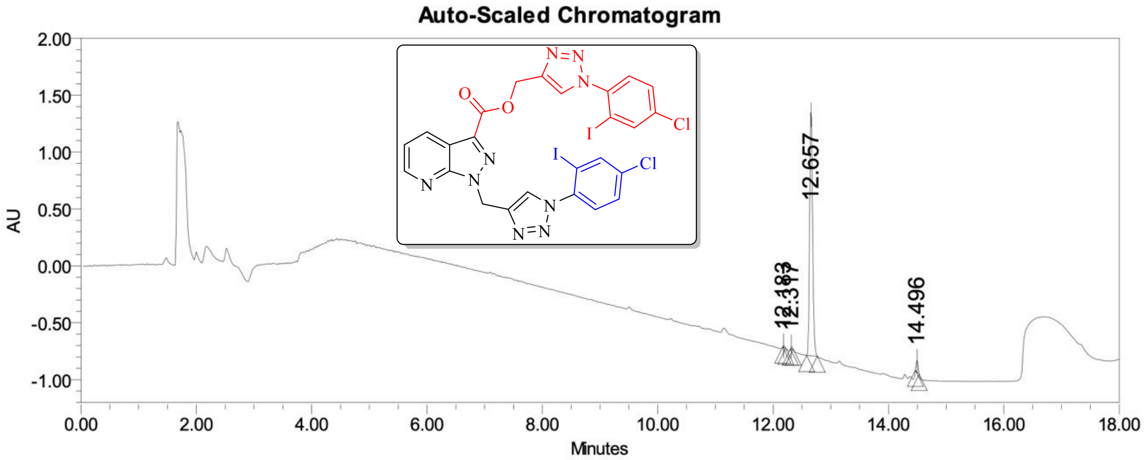

Peak Results

	Name	RT	Area	% Area
1		12.183	25667	0.32
2		12.317	40781	0.50
3		12.657	7807837	96.28
4		14.496	234849	2.90

^{1}H NMR spectrum of *(1-(3-Chlorophenyl)-1H-1,2,3-triazol-4-yl)methyl 1-((1-(3-chlorophenyl)-1H-1,2,3-triazol-4-yl)methyl)-1H-pyrazolo[3,4-b]pyridine-3-carboxylate* (31)

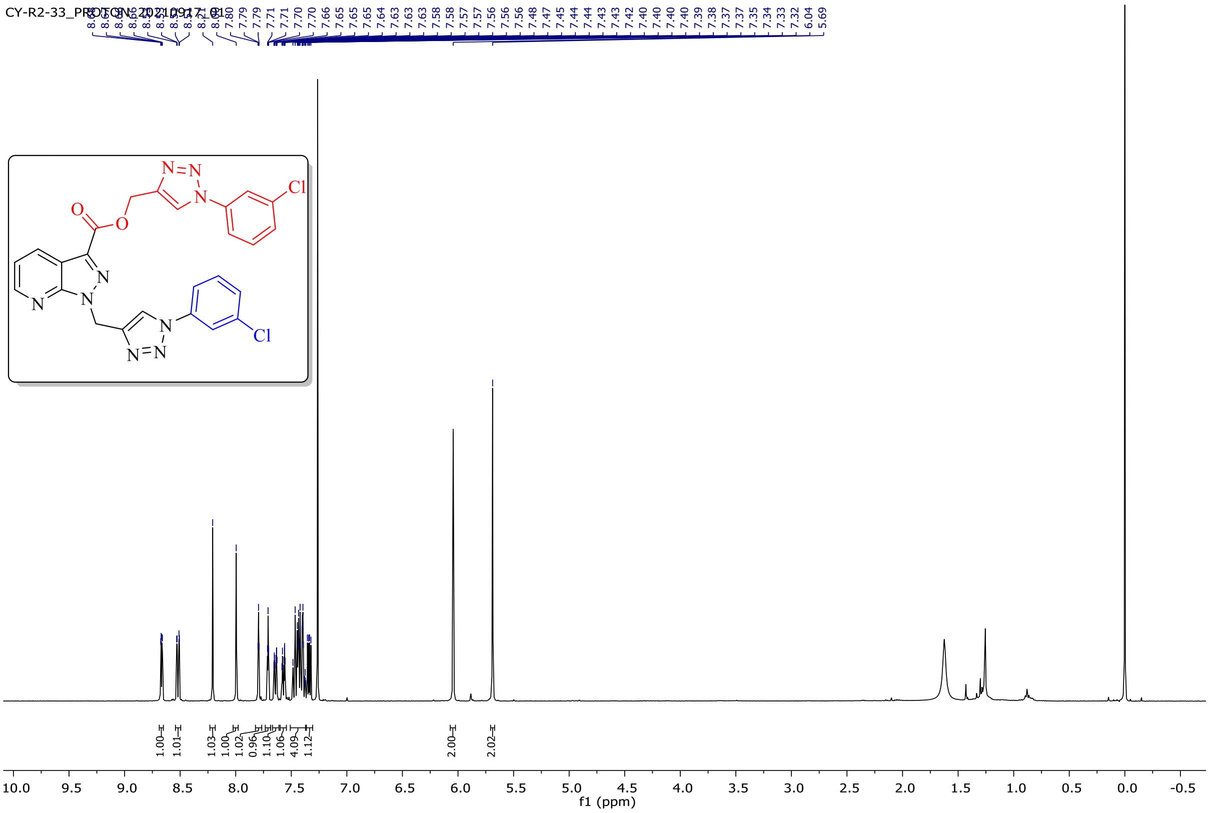

^{13}C NMR spectrum of *(1-(3-Chlorophenyl)-1H-1,2,3-triazol-4-yl)methyl 1-((1-(3-chlorophenyl)-1H-1,2,3-triazol-4-yl)methyl)-1H-pyrazolo[3,4-b]pyridine-3-carboxylate* (31)

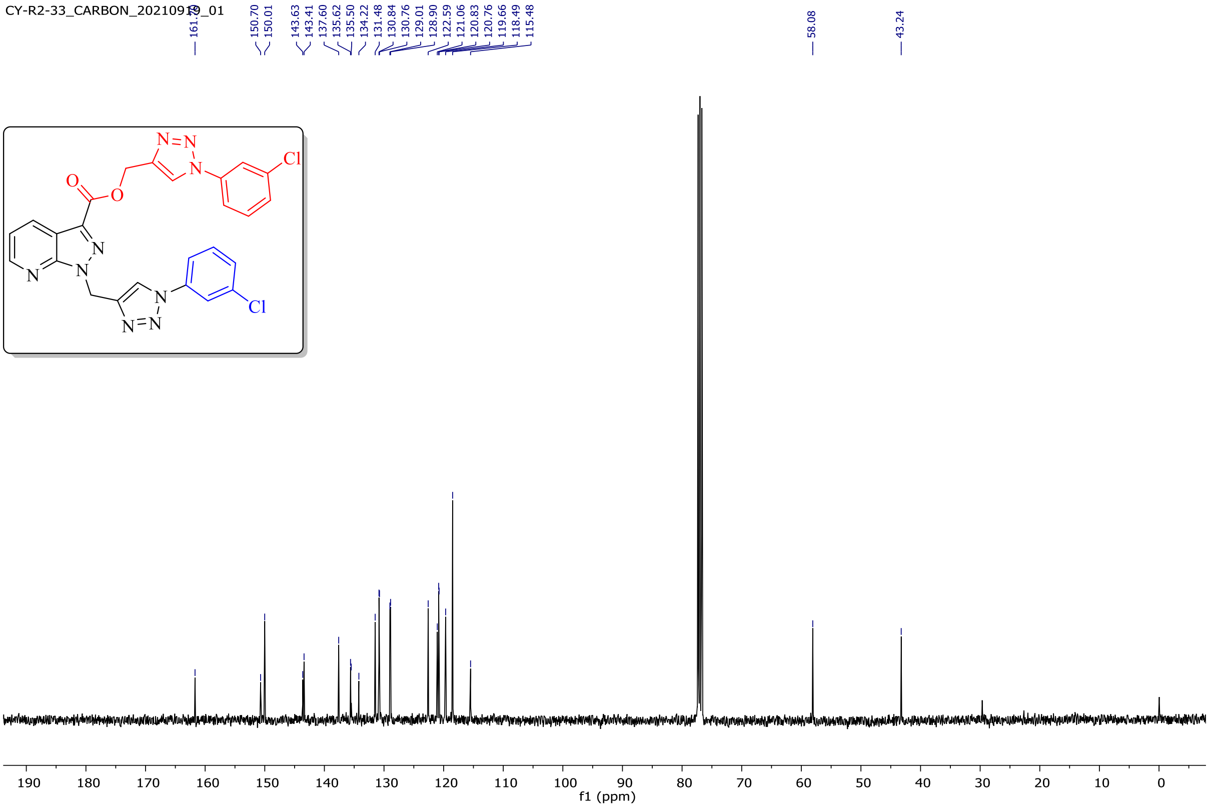

IR spectrum of *(1-(3-Chlorophenyl)-1H-1,2,3-triazol-4-yl)methyl 1-((1-(3-chlorophenyl)-1H-1,2,3-triazol-4-yl)methyl)-1H-pyrazolo[3,4-b]pyridine-3-carboxylate* (31)

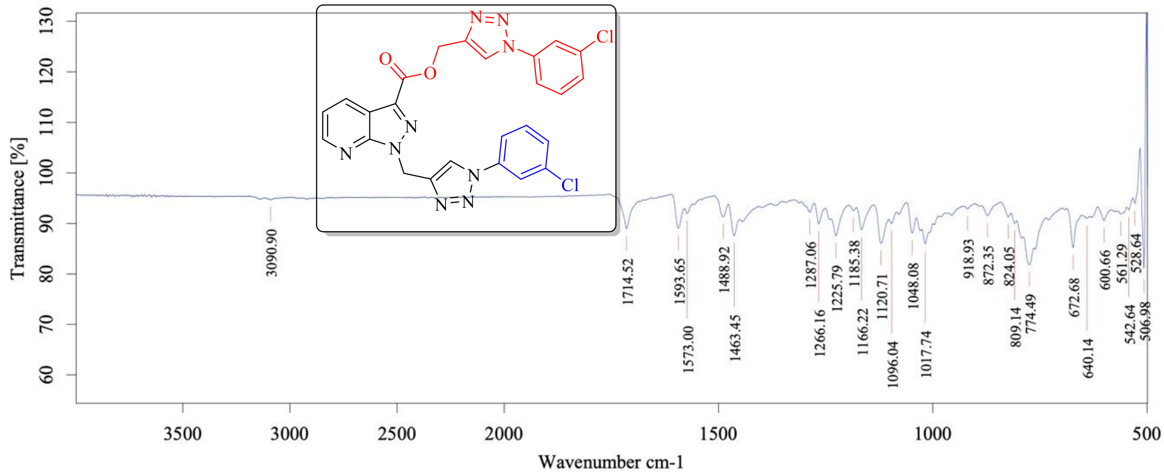

Mass spectrum of *(1-(3-Chlorophenyl)-1H-1,2,3-triazol-4-yl)methyl 1-((1-(3-chlorophenyl)-1H-1,2,3-triazol-4-yl)methyl)-1H-pyrazolo[3,4-b]pyridine-3-carboxylate* (31)

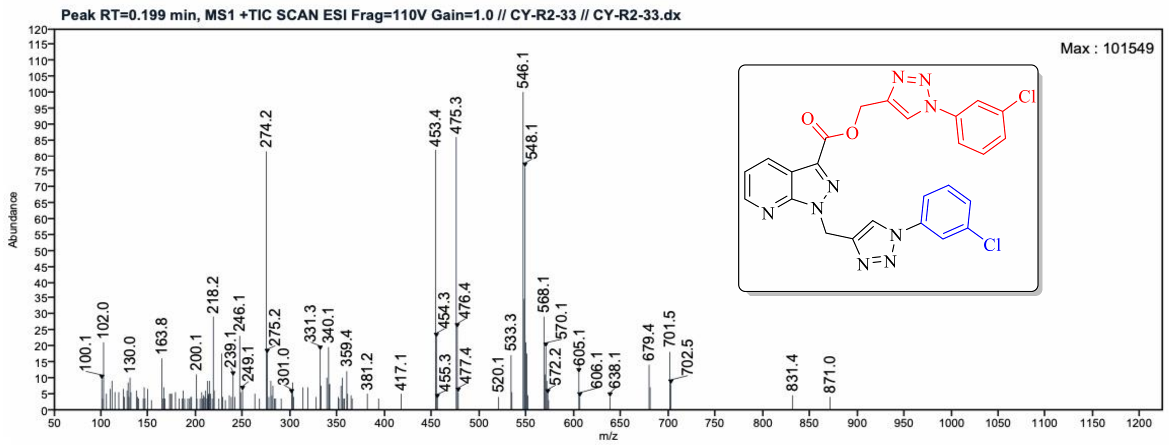

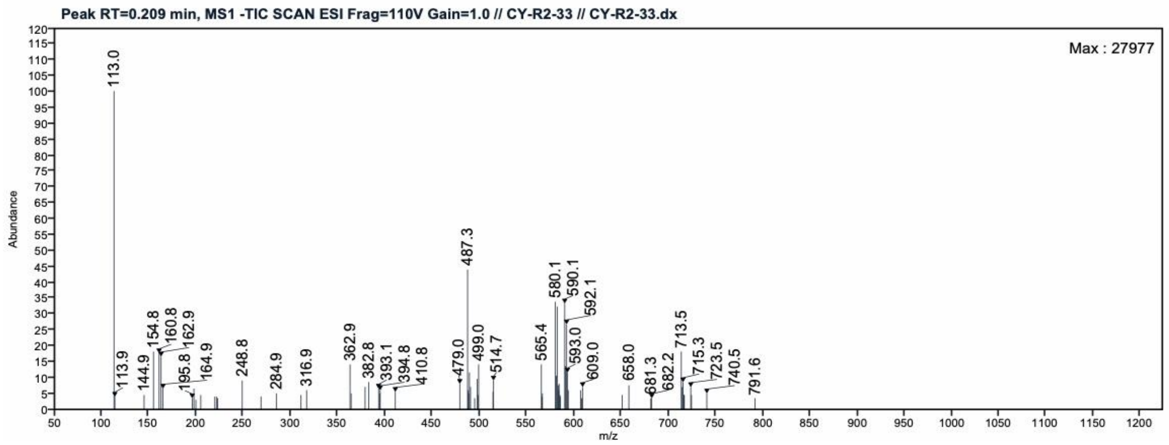

HPLC chromatogram of *(1-(3-Chlorophenyl)-1H-1,2,3-triazol-4-yl)methyl 1-((1-(3-chlorophenyl)-1H-1,2,3-triazol-4-yl)methyl)-1H-pyrazolo[3,4-b]pyridine-3-carboxylate* (31)

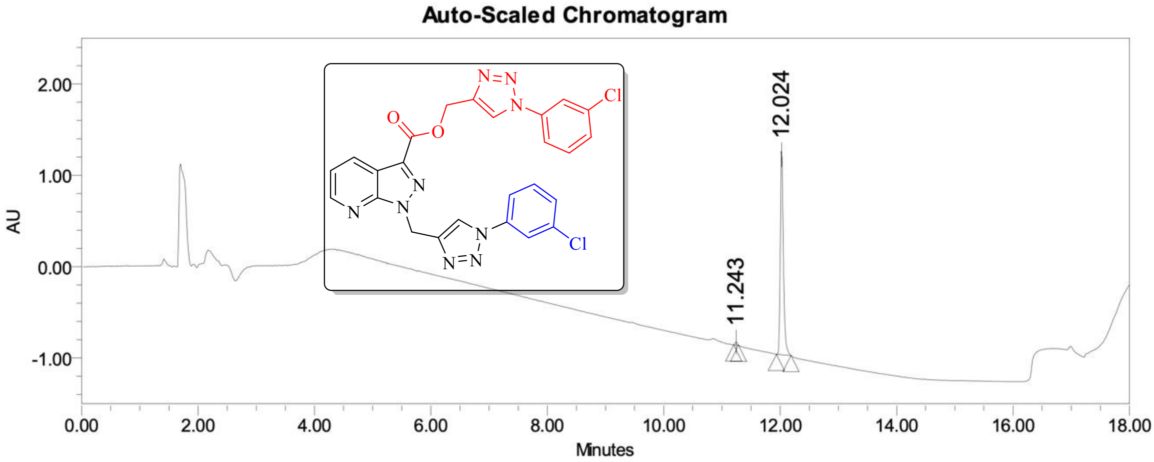

Peak Results

	Name	RT	Area	% Area
1		11.243	94473	1.13
2		12.024	8264804	98.87
